# Supplementary material for: Nanoscale 3D spatial addressing and valence control of quantum dots using wireframe DNA origami
Source: Nat Commun. 2022 Aug 23;13:4935. doi: 10.1038/s41467-022-32662-w (PMC9399249; doi:10.1038/s41467-022-32662-w)
Supplement: Supplementary file 1 — Supplementary Information [file 41467_2022_32662_MOESM1_ESM.pdf]

Supplementary Information for

Nanoscale 3D Spatial Addressing and Valence Control of Quantum Dots using Wireframe DNA Origami

Chi Chen[1], Xingfei Wei[2], Molly F. Parsons[1], Jiajia Guo[3,†], James L. Banal[1,‡], Yinong Zhao[4], Madelyn N. Scott[3], Gabriela S. Schlau-Cohen[3], Rigoberto Hernandez[2,4,5], Mark Bathe[1*]

[1]Department of Biological Engineering, Massachusetts Institute of Technology, Cambridge, MA, 02139, USA
[2]Department of Chemistry, Johns Hopkins University, Baltimore, MD, 21218, USA
[3]Department of Chemistry, Massachusetts Institute of Technology, Cambridge, MA, 02139, USA
[4]Department of Chemical & Biomolecular Engineering, Johns Hopkins University, Baltimore, MD, 21218, USA
[5]Department of Materials Science and Engineering, Johns Hopkins University, Baltimore, MD, 21218, USA
[†] Present address: Bionic Sensing and Intelligence Center, Institute of Biomedical and Health Engineering, Shenzhen Institutes of Advanced Technology, Chinese Academy of Sciences, Shenzhen, 518055, China
[‡] Present address: Cache DNA, Inc., 200 Lincoln Centre Drive, Foster City, California 94404 USA

* Correspondence should be addressed to: mark.bathe@mit.edu

Contents

Supplementary Methods 5

Sample preparation for QD-dye FRET constructs 5

Sample preparation for constructs used to investigate the effect of ps tract length 5

Sample preparation for QD dimers 6

Sequence design for QD-wireframe DNA origami constructs 6

Sample preparation for QD-DNA complex constructs 8

Molecular dynamics (MD) simulation 9

Supplementary Figures 11

Overview of QD surface modification for ssDNA wrapping 11

TEM images of commercially-available QD 12

TEM images of QD after surface modification 13

Photoluminescence (PL) spectra and FRET efficiency of QD-dye FRET systems 14

Absorption spectra of QD-dye FRET systems 15

Time-resolved decay curves of QD-dye FRET systems 16

QD wrapped by ssDNA with different ps-backbone length and the fixed po-backbone length 17

QD-dye FRET in a distal configuration 18

QD600 wrapped by ssDNA with various ps-backbone length and the fixed total length 19

The A* tract can effectively prevent nonspecific DNA adsorption 20

MD simulation model for A* tract length effect 21

MD simulation model for ssDNA wrapped QD 22

TEM images of QD dimer 23

QD600 wrapped by ssDNA with various ps-backbone length and the fixed total length 24

MD simulation model for the effect of QD size 25

TEM images of commercially-available streptavidin QD. 26

Streptavidin QD-dye FRET system 27

AGE images of Pep wireframe with biotin domain and ps-backbone wrapping domain 28

AGE images of conjugation of streptavidin QD655 and Pep wireframe DNA origami objects with biotin domain 29

TEM images of Pep-30 nt A*-QD630 assemblies using ps-backbone based wrapping domain at the inner center 30

TEM images of Pep-30 nt A*-QD630 assemblies using ps-backbone based wrapping domain at the outer edge 31

TEM images of Pep-biotin-streptavidin QD655 assemblies using biotin domain at the inner center. 32

TEM images of Pep-biotin-streptavidin QD655 assemblies using biotin domain at the outer edge. 33

The yield of Pep-QD assemblies using ssDNA wrapping and biotin-streptavidin conjugation 34

Schematic for wireframe DNA origami objects folding and preparation of valence-geocoded QD 35

AGE image of Tet wireframe DNA origami objects folding 36
AGE image of Pep wireframe DNA origami objects folding 37
TEM images of Tet wireframe DNA origami objects 38
TEM images of Tet-QD600 assemblies 39
TEM images of Pep wireframe DNA origami objects 40
TEM images of Pep-QD660 assemblies 41
AGE images of Tet-QD600 assemblies 42
AGE images of Pep-QD660 assemblies 43
D-space measuring for Tet-QD600 assemblies 44
D-space measuring for Pep-QD660 assemblies 45
Incubation molar ratio effect 46
Schematic of DNA complex-QD-based FRET network and colloidal molecules 47
Formation of chimeric DNA complex 48
Formation of DNA complex-QD constructs 49
Spectroscopic characterization of Tet-QD-dye and DNA complex-QD-dye-based concentric FRET network 50
Spatial addressable dye labeled Pep wireframe DNA origami objects 51
Distance between QD and dye on Pep wireframe objects 52
AGE images of Pep wireframe DNA origami objects-based FRET network 53
Pep wireframe DNA origami objects-based AF647-AF750 FRET 54
Spectroscopic characterization of Pep-30 nt A*-QD600-AF647-AF750 and Pep-biotin-streptavidin QD605-AF647-AF750-based multi-step FRET networks 55
TEM images of QD trimer type-A 56
TEM images of QD trimer type-B 57
TEM images of QD tetramer 58
TEM images of QD pentamer 59
TEM images of QD hexamer 60
Statistical distributions of different QD-based colloidal molecules 61
MD simulation model with atom labels and partial charges 62
Supplementary Tables 63
Supplementary Table 1. Comparison of various DNA-based strategies to engineer valences on QDs .. 63
Supplementary Table 2. Photophysical Properties of the FRET Compounds within the DNA wrapping experiment[a] 64
Supplementary Table 3. P-values, t statistic, and degree of freedom for Fig. 5d. 65
Supplementary Table 4. DNA sequences for QD wrapping and QD-dye FRET pairs. 66
Supplementary Table 5. DNA sequences for ps tract length effect. 67
Supplementary Table 6. Scaffold sequences for wireframe DNA origami objects. 68

Supplementary Table 7. Staple sequences for the tetrahedron of 52-bp edge length and the same tetrahedron with overhangs. 69
Supplementary Table 8. Staple sequences for the pentagonal pyramid of 63-bp edge length and the same pentagonal pyramid with DNA wrapping domain. 70
Supplementary Table 9. DNA sequences for magnetic capture and toehold mediated strand displacement. 71
Supplementary Table 10. DNA sequences for QD-DNA complex constructs. 72
Supplementary Table 11. Atomic mass and general LJ force field parameters. 73
Supplementary Table 12. LJ force field parameters for interfacial interactions. 74
Supplementary Table 13. Harmonic bond force field. 75
Supplementary Table 14. Harmonic angle force field. 76
Supplementary Table 15. Harmonic dihedral force field. 77
Supplementary Table 16. CVFF improper force field. 78
Supplementary References 79

Supplementary Methods

Sample preparation for QD-dye FRET constructs

QDs with neutral surface charge were incubated with ps-backbone-modified ssDNA or 3'-thiolated-ssDNA overnight for DNA complexation. Briefly, ten 2-μL portions (20 μL total) of chimeric ssDNA (0.1 mM) with different ps tract length (po1-(TT)-5A*, po1-(TT)-10A*, and po1-(TT)-30A* for QD600; po1-(TT)-5A*, po1-(TT)-10A*, and po1-(TT)-30A* for QD630; po1-(TT)-5A*, po1-(TT)-30A*, and po1-(TT)-50A* for QD660) and fixed po tract length (23 nt) or 3'-thiolated-ssDNA (po1-(TTTTT)-SH) were added to 500 μL of 400 nM QD with neutral-surface-charge in 10 mM Tris.

To fabricate QD600-AF647 and QD630-AF647 FRET pairs, QD600-5 nt A*, QD600-10 nt A*, QD600-30 nt A*, QD600-thiol, QD630-5 nt A*, QD630-10 nt A*, QD630-30 nt A*, and QD630-thiol were incubated with AF647-po(c1), respectively. To fabricate QD660-AF750 FRET pairs, QD660-5 nt A*, QD660-30 nt A*, QD660-50 nt A* and QD660-thiol were incubated with AF750-po(c1), respectively. All DNA sequences are summarized in Supplementary Table 4.

Sample preparation for streptavidin QD-dye FRET constructs

Streptavidin QD-dye FRET constructs were prepared as described previously with minor modifications[1]. To fabricate biotin and AF647 modified DNA duplex, 5'-AF647 labeled ssDNA (AF647-po(c1)) and 3'-biotin-labeled complementary ssDNA (po1-(TT)-biotin) were diluted to 2 μM in 1 × PBS buffer, and then incubated using the following protocol: 95 °C for 5 min, 90 °C for 10 min, then directly cooled down to 4 °C in refrigerator. To fabricate 100 μL streptavidin QD-dye FRET constructs (20 nM), 2 μL of streptavidin QD605 (1 μM) were incubated with 50 μL biotin and AF647 modified DNA duplex (2 μM) in 1× PBS buffer at 37 °C for 30 min to form the QD-streptavidin-biotin-AF647 constructs. All DNA sequences are summarized in Supplementary Table 4.

Sample preparation for constructs used to investigate the effect of ps tract length

For QD600 samples, 5-μL of 30A-random (po), 5A*-random(po), 10A*-random(po), 20A*-random(po), 30A*-random(po), 40A*-random(po), or SH-random(po) were added (divided into

ten additions) to 100 μL of QD600 with neutral surface charge (100 nM in 10 mM Tris). The concentrations of all DNA strands were normalized to 100 μM.

For QD660 samples, 5-μL portions of 5A*-random(po), 10A*-random(po), 20A*-random(po), 30A*-random(po), 40A*-random(po) was added (divided into ten additions) to QD660 (10 nM in 10 mM Tris). The concentrations of all DNA strands were normalized to 10 μM. DNA sequences are summarized in Supplementary Table 5.

Sample preparation for QD dimers

For QD dimers, the ssDNA (po1-(TT)-30A*)-wrapped QD600 was incubated with complementary ssDNA (po(c1)-(TT)-30A*)-wrapped QD600 (molar ratio was 1:1, in 25 mM NaCl, 10 mM Tris). After overnight reaction, the mixture without purification was drop-casted on 400 mesh carbon film square grids (fisher scientific, catalog number: 5024891) for TEM imaging.

Sequence design for QD-wireframe DNA origami constructs

Tet-QD600. Tetrahedron (Tet) wireframe DNA origami objects with six-helix bundle edge were folded in a solution of 50 nM scaffold (phPB84, sequence in Supplementary Table 6), 500 nM staples, 1× TAE, and 12 mM $MgCl_2$ and annealed over the course of 13 h. For Tet-QD600 constructs, staple 38 was replaced with staple 38' (Supplementary Table 7).

Tet-QD600-based FRET network. Tet wireframe DNA origami objects with 1–4 overhangs were incubated with AF647-labelled complementary ssDNA (molar ratio was 1:10, in 1× TAE with 20 mM $MgCl_2$) at room temperature. For 1-AF647-labeled Tet wireframe DNA origami objects design, staples 12 and 38 were replaced with staples 12' and 38', respectively. For 2-AF647-labeled Tet wireframe DNA origami objects design, staples 12, 15 and 38 were replaced with staples 12', 15' and 38', respectively. For 3-AF647-labeled Tet wireframe DNA origami objects, staples 12, 15, 25, and 38 were replaced with staples 12', 15', 25' and 38', respectively. For 4-AF647-labeled Tet wireframe DNA origami objects design, staples 12, 15, 25, 32, and 38 were replaced with staples 12', 15', 25', 32' and 38', respectively. All staple sequences are listed in Supplementary Table 7.

Pep-QD. Pentagonal pyramid (Pep) wireframe DNA origami objects with two-helix bundle edges were folded in a solution of 30 nM scaffold (pF1A, sequence in Supplementary Table 6), 300 nM staples, 1× TAE, and 12 mM $MgCl_2$ and annealed over the course of 2 h. For Pep-QD660 assemblies design, the staple 17 was replaced with staple 17'. For Pep-30nt A*-QD630 at the outer edge assemblies design, the staple 33 was replaced with staple 33'. For Pep-30nt A*-QD630 at the inner center assemblies design, the staple 9 was replaced with staple 9'. For Pep-biotin-streptavidin-QD655 at the inner center assemblies design, the staples 9 were replaced with staples 9-biotin. For Pep-biotin-streptavidin-QD655 at the outer edge assemblies design, the staple 33 was replaced with staple biotin-33 (Supplementary Table 8).

Pep-QD-AF647-AF750-based concentric multi-step FRET networks. Pep wireframe DNA origami objects (with or without AF647) with spatial addressable overhangs were incubated with AF750-labelled complementary ssDNA (molar ratio was 1:2, in 1× TAE with 12 mM $MgCl_2$) at room temperature. For Pep-30 nt A*-QD600-AF647-AF750 Type A, staples 8, 9 and 10 were replaced with staples AF647-8, 9', and 10' respectively. For Pep-30 nt A*-QD600-AF647-AF750 Type B, staples 7, 8 and 9 were replaced with staples 7', AF647-8 and 9', respectively. For Pep-30 nt A*-QD600-AF647-AF750 Type C, staples 8, 9, and 17 were replaced with staples AF647-8, 9' and 17', respectively. For Pep-30 nt A*-QD600-AF750 Type A, staples 9 and 10 were replaced with staples 9' and 10' respectively. For Pep-30 nt A*-QD600-AF750 Type B, staples 7 and 9 were replaced with staples 7' and 9' respectively. For Pep-30 nt A*-QD600-AF750 Type C, staples 9 and 17 were replaced with staples 9' and 17' respectively. For Pep-biotin-streptavidin-QD605-AF647-AF750 Type A, staples 8, 9 and 10 were replaced with staples AF647-8, 9'-biotin, and 10' respectively. For Pep-biotin-streptavidin-QD605-AF647-AF750 Type B, staples 7, 8 and 9 were replaced with staples 7', AF647-8 and 9-biotin, respectively. For Pep-biotin-streptavidin-QD605-AF647-AF750 Type C, staples 8, 9, and 17 were replaced with staples AF647-8, 9-biotin and 17', respectively. For Pep-biotin-streptavidin-QD605-AF750 Type A, staples 9 and 10 were replaced with staples 9-biotin and 10' respectively. For Pep-biotin-streptavidin-QD605-AF750 Type B, staples 7 and 9 were replaced with staples 7' and 9-biotin, respectively. For Pep-biotin-streptavidin-QD605-AF750 Type C, staples 9 and 17 were replaced with staples 9-biotin and 17' respectively. All staple sequences are listed in Supplementary Table 8.

Pep-QD600-based colloidal molecules. Pep wireframe DNA origami objects with ps-backbone overhangs on geocoded points were incubated with excess QD600 (molar ratio was 1:5). For trimer Type A design, staples 3, 17, 27 and 33 were replaced with staples 3', 17', 27' and 33', respectively. For trimer Type B design, staples 3, 4, 17 and 20 were replaced with staples 3', 4', 17' and 20', respectively. For tetramer design, staples 3, 14, 17, 27 and 33 were replaced with staples 3', 14', 17', 27' and 33', respectively. For pentamer design, staples 3, 4, 17, 20, 27 and 33 were replaced with staples 3', 4', 17', 20', 27' and 33', respectively. For hexamer design, staples 3, 4, 14, 17, 20, 27 and 33 were replaced with staples 3', 4', 14', 17', 20', 27' and 33', respectively. All staple sequences are summarized in Supplementary Table 8. The mixtures were purified using toehold-mediated strand displacement based magnetic separation. Briefly, 1 μL of 3'-biotin-labeled bead strand sequence (10 μM; Supplementary Table 9) was added to 50 μL of unpurified colloidal molecules (100 nM). After overnight reaction, the mixture was incubated with 5 μL of Dynabeads™ M-270 Streptavidin (10 mg/mL, ≥ 950 pmoles free biotin/mg beads) (Invitrogen™, catalog number: 65306). After overnight reaction and shaking, the colloidal molecules and magnetic bead assemblies were captured by DynaMag™-PCR Magnet (Invitrogen™, catalog number: 492025) and washed using 1× TAE and 12 mM $MgCl_2$ until no fluorescence can be observed in supernatant. A volume of 50 μL of bead invader sequence (2 μM in 1× TAE and 12 mM $MgCl_2$, in Supplementary Table 9) was added. Molar ratio of colloidal molecules:bead strand:bead invader was 1:2:20.

Sample preparation for QD-DNA complex constructs

QD-DNA complex constructs were prepared as described previously with minor modifications[2]. Equimolar concentrations of the indicated strands for each construct were mixed together in 1× PBS buffer with a final volume of 50 μL:

Valence (I) DNA complex. po1-DNA1, DNA2, DNA3, and DNA4

Valence (II) (Type C) DNA complex. po1-DNA1, po1-DNA2, DNA3, and DNA4

Valence (II) (Type D) DNA complex. po1-DNA1, DNA2, po1-DNA3, and DNA4

Valence (III) DNA complex. po1-DNA1, po1-DNA2, po1-DNA3, and DNA4

Valence (IV) DNA complex. po1-DNA1, po1-DNA2, po1-DNA3, and po1-DNA4

All DNA sequences are listed in Supplementary Table 10. The resulting DNA complex mixtures were annealed using the following protocol: 95 °C for 5 min, 90 °C for 10 min, then directly cooled down to 4 °C in refrigerator. 15 μL of unpurified mixtures were combined with 3 μL of TriTrack DNA loading dye (Thermo Scientific™, catalog number: R1161) and loaded to a 3% agarose gel with 1× TBE and 1× SYBR Safe (ThermoFisher, Waltham, MA).

To prepare QD-DNA complex constructs with valence (I)–(IV), the prepared DNA complex with valence (I)–(IV) were incubated with QD (molar ratio was 1.2:1 in 1× PBS) overnight. To fabricate DNA complex-QD-dye FRET pairs, DNA complex-QD with valence (I)–(IV) were incubated with complementary DNA labeled with AF647 in 1× PBS buffer. All sequences are listed in Supplementary Table 4. After 2-hour incubation, the fluorescence emission spectra of complex-QD alone and in the presence of AF647 were recorded.

To fabricate the DNA complex-based QD trimers, DNA complex-QD with valence (II) (Type C and Type D) were incubated with complementary DNA labeled monovalent QD in 1× PBS buffer. All sequences are listed in Supplementary Table 10. After overnight reaction, the unpurified mixtures were drop casted on glow-discharged 400 mesh carbon film square grids for TEM characterization.

Molecular dynamics (MD) simulation

The poly adenine (polyA) chains with 28–53 repeating units were built using the BIOVIA Materials Studio (MS) software[3]. The consistent-valence force field was used and the parameters were directly adopted from the MS software with slight modification. The simulation model with atom labels and partial charges are shown in Supplementary Fig. 52. Supplementary Tables 11–16 list the force parameters and the labels are in Supplementary Fig. 52. All MD simulations were carried out using the Large-scale Atomic Molecular Massively Parallel Simulator (LAMMPS) package[4]. Lennard-Jones (LJ) energy is calculated by Supplementary Eq. (S1). Bonding energy is calculated by Supplementary Eq. (S2), Angle energy is calculated by Supplementary Eq. (S3), Dihedral energy is calculated by Supplementary Eq. (S4). Improper energy is calculated by Supplementary Eq. (S5).

$$E_{\mathrm{LJ}} = 4\varepsilon\left[(\frac{\sigma}{r})^{12} - \frac{\sigma^6}{r}\right],\ \ r < r_{\mathrm{c}} \quad \text{(S1)}$$

where r_{c} is 10 Å.

$$E_{\mathrm{b}} = K_{\mathrm{b}}(r - r_0)^2 \quad \text{(S2)}$$

$$E_{\mathrm{a}} = K_{\mathrm{a}}(\theta - \theta_0)^2(\frac{\pi}{180})^2 \quad \text{(S3)}$$

$$E_{\mathrm{d}} = K_{\mathrm{d}}[1 + d * \cos(n\phi)] \quad \text{(S4)}$$

$$E_{\mathrm{i}} = K_{\mathrm{i}}[1 + d * \cos(n\phi)] \quad \text{(S5)}$$

All the simulations were carried under *NVT* ensemble with the Langevin thermostat. At first, we used 10 ns simulation time at 3000 K to relax the ssDNA chain and to make the counterions (Na^+) evenly distributed in the box. The rest simulations were conducted at 300 K. Next step, a steering force of 0.1 kcal/mol/Å was applied on the ssDNA for 5 ns to drag it near the ZnS nanoparticle (NP). The cutoff of the steering force was 15 Å above the ZnS NP surface. However, at this step the ssDNA was normally collapsed on the ZnS NP surface. By setting the LJ interaction energy between S atoms of ssDNA and Zn atoms of ZnS NP (S-Zn) to zero, the ssDNA chain turned into an open conformation near the ZnS NP surface. The chain conformation was further opened by applying a -0.1 kcal/mol/Å dragging force (a pushing force) on the ssDNA for 0.5 ns. Then the steering force was set at 0.1 kcal/mol/Å to drag the ssDNA again. Meanwhile, the S-Zn LJ interaction energy was set back to 10 kcal/mol to make the ssDNA binding to the ZnS NP. To further relax the attached ssDNA, then we set the S-Zn LJ interaction energy to 3 kcal/mol to allow the ssDNA chain able to migrate on the ZnS NP, but not leaving the surface. The last dragging step was applying a steering force for 1 ns on the S atoms of the ssDNA at 0.5 kcal/mol/Å towards the ZnS NP center, with a cutoff 10 Å above the ZnS NP surface, and then it was followed by a 5 ns relaxing. Data was collected by a continuous simulation of 10 ns at 300 K under *NVT* ensemble for four times with different random seeds and also by dragging the ssDNA on to the ZnS NP multiple times to generate a completely different initial structure. In Supplementary Fig. 12, for example, the 6 nm QD simulation runs at ~2.5 ns/day using 24 CPU cores on one CPU node. Additional QDs with diameters up to 14 nm are reported in Figure 15. Simulations of 14 nm diameter QDs run at ~0.95 ns/day using 1 GPU card on one GPU node.

Supplementary Figures

Overview of QD surface modification for ssDNA wrapping

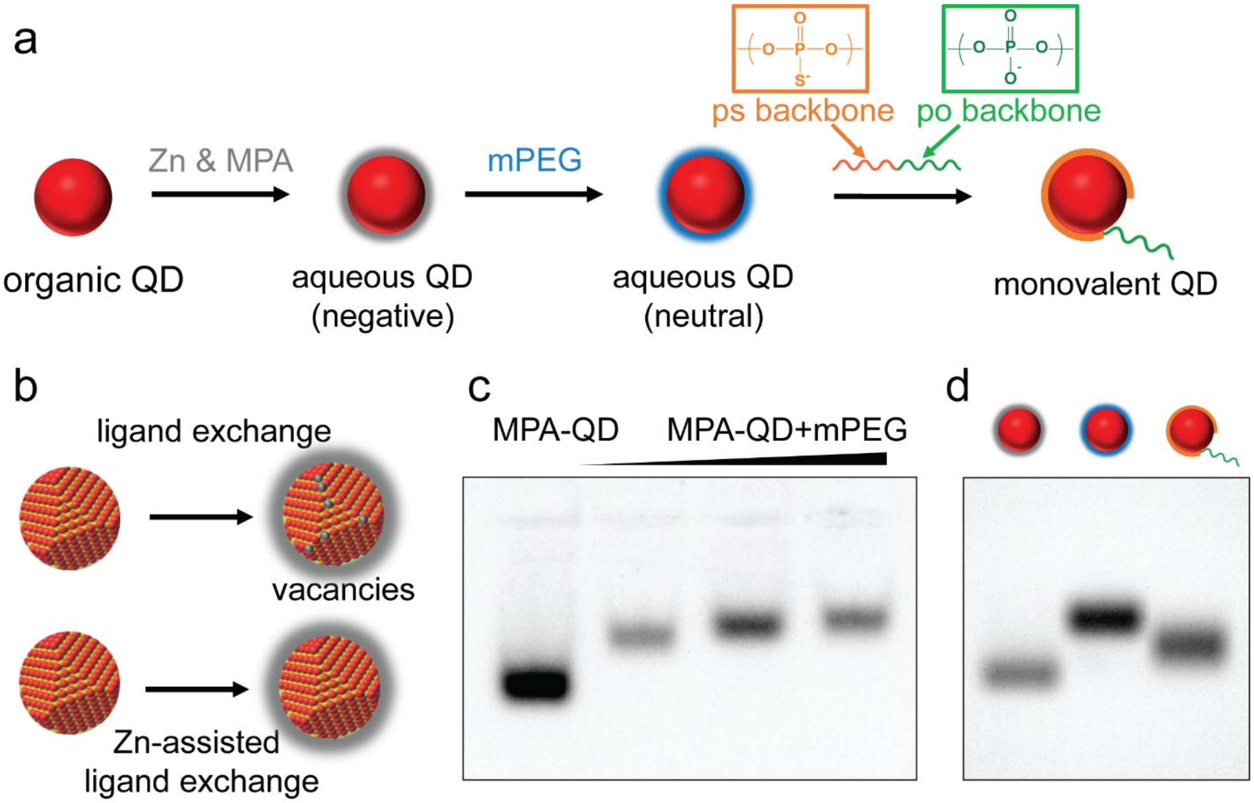

Supplementary Fig. 1. a) Schematic for the strategy of QD surface modification for ssDNA wrapping. b) Schematic of the Zn-assisted ligand exchange. c) Agarose gel electrophoresis (AGE) (0.8%) image shows the tunable surface charge of QD capped with MPA/mPEG with different ratio. d) AGE (0.8%) image of QD capped with MPA alone (left), QD before (middle) and after ssDNA wrapping (right). Source data are provided as Source Data file.

TEM images of commercially-available QD

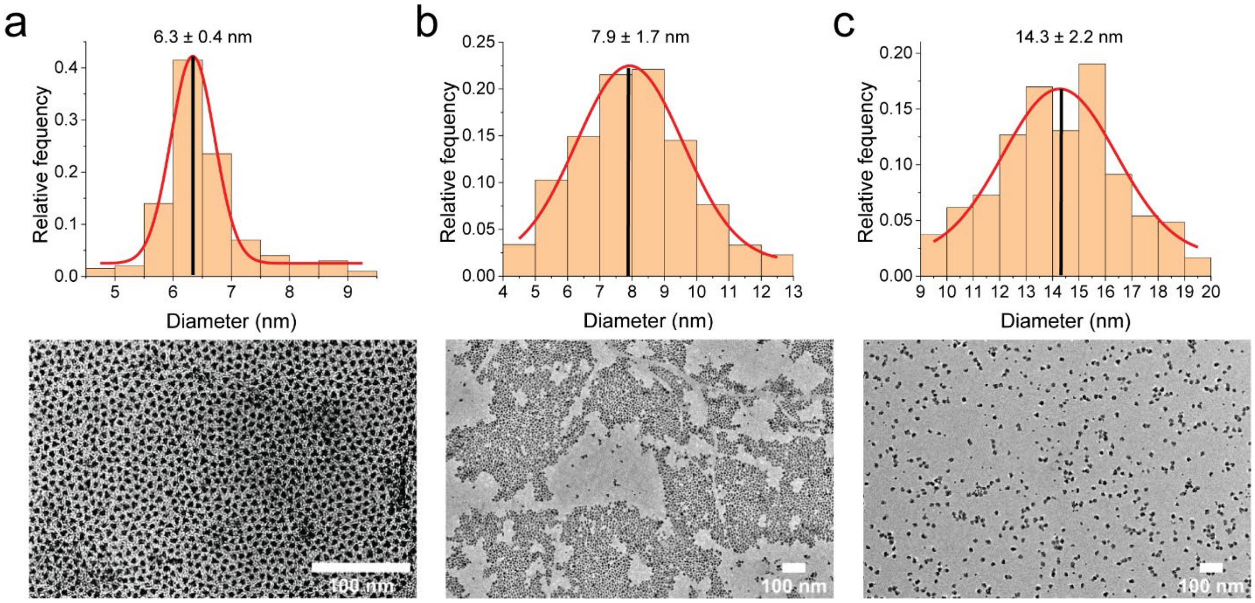

Supplementary Fig. 2. TEM images (bottom) and size distribution analysis (top) for commercial a) QD600, b) QD630, and c) QD 660. Particle diameters were calculated from 501 NPs (a), 1923 NPs (b), and 536 NPs (c), respectively. Red curves are Gaussian fit to the measured particle diameter distribution. Indicated particle diameters for each histogram are expressed as mean ± standard deviation. Source data are provided as Source Data file.

TEM images of QD after surface modification

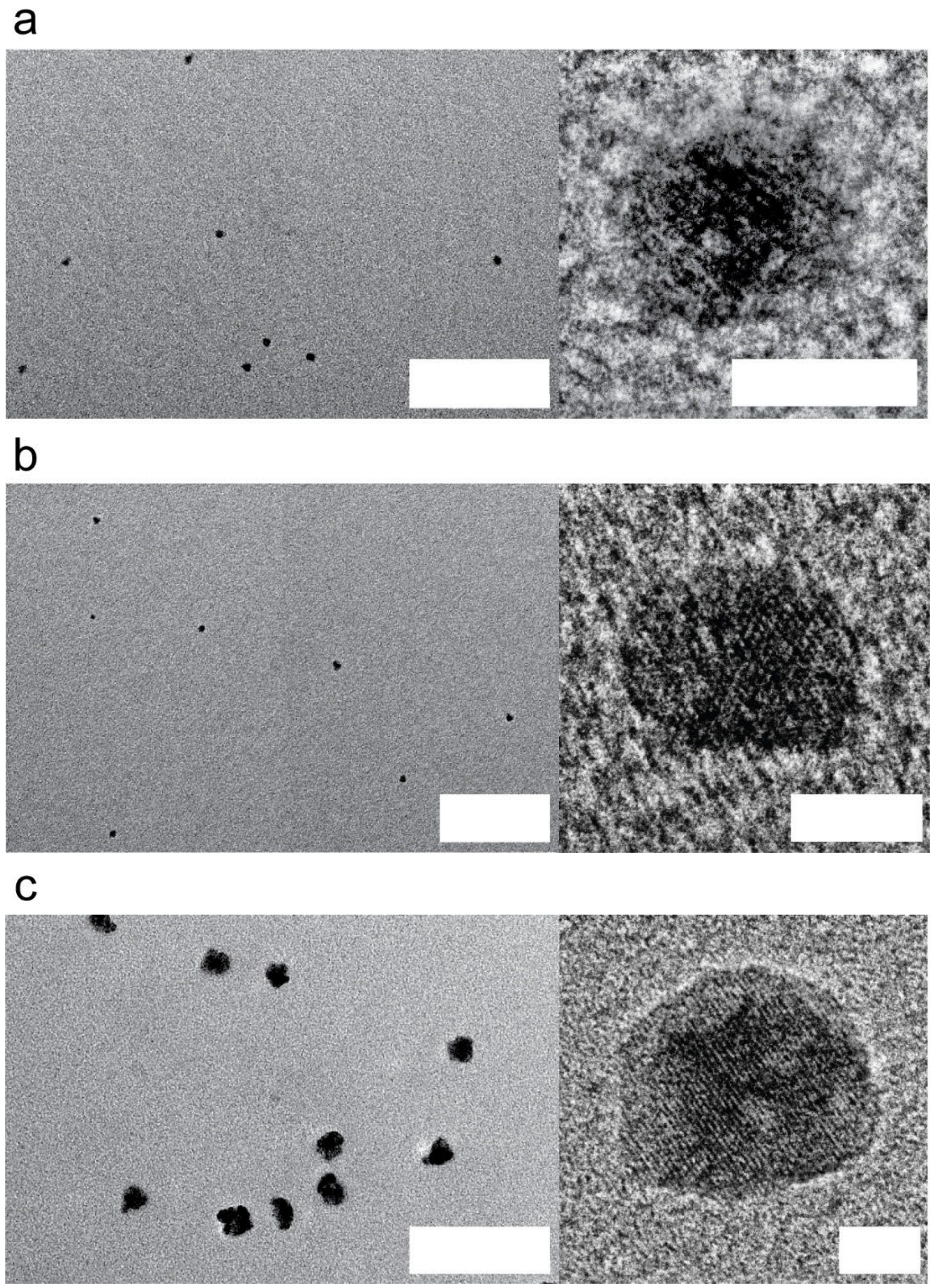

Supplementary Fig. 3. TEM (left) and HRTEM (right) images of a) QD600, b) QD630, and c) QD660 after surface modification. (Scale bars: 100 nm (left) and 5 nm (right))

Photoluminescence (PL) spectra and FRET efficiency of QD-dye FRET systems

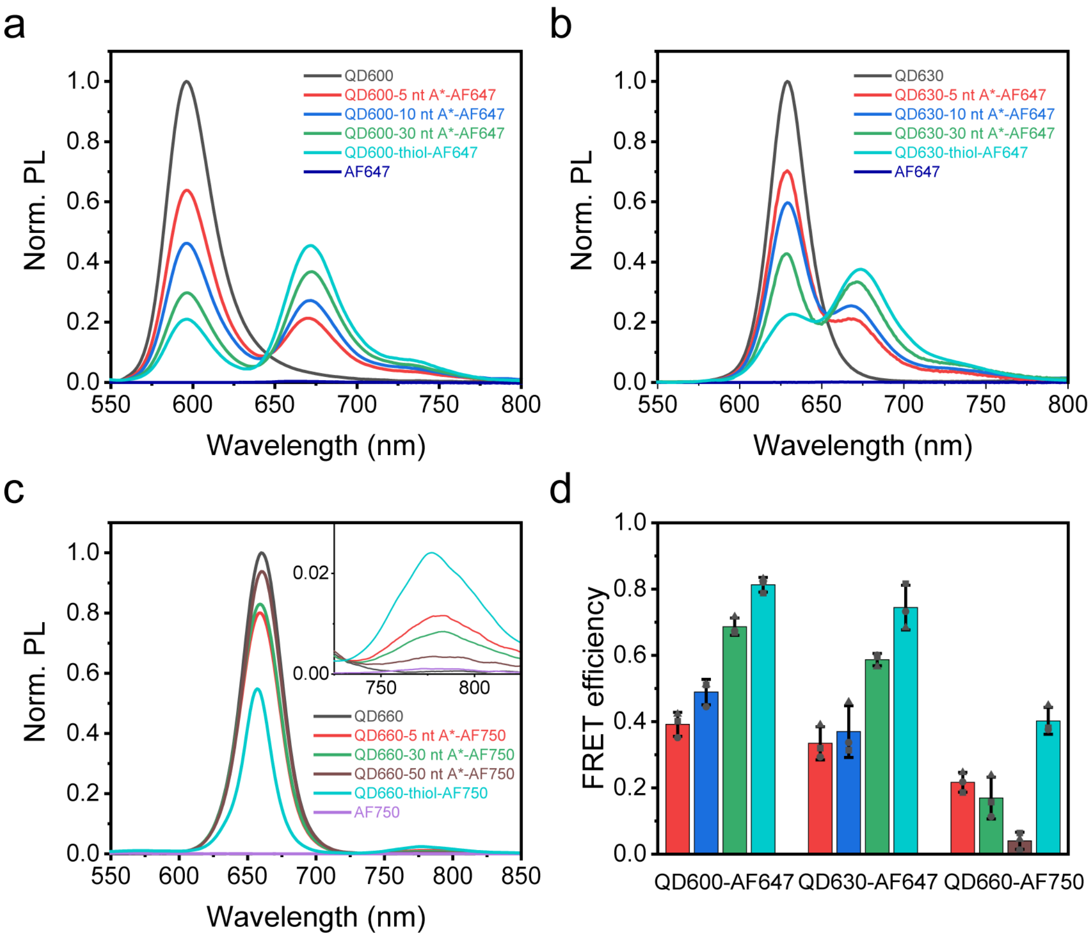

Supplementary Fig. 4. Photoluminescence (PL) spectra of a) QD600-AF647, b) QD630-AF647, and c) QD660-AF750 FRET pairs. d) FRET efficiency calculated from each FRET system using various ps backbone length and 3'-thiolated ssDNA (red: 5A*, blue: 10A*, green: 30A*, brown: 50A*, and cyan: 3'-thiolated-ssDNA). Error bars represent standard deviation of the mean (n = 3 replicates per group). Source data are provided as Source Data file.

Absorption spectra of QD-dye FRET systems

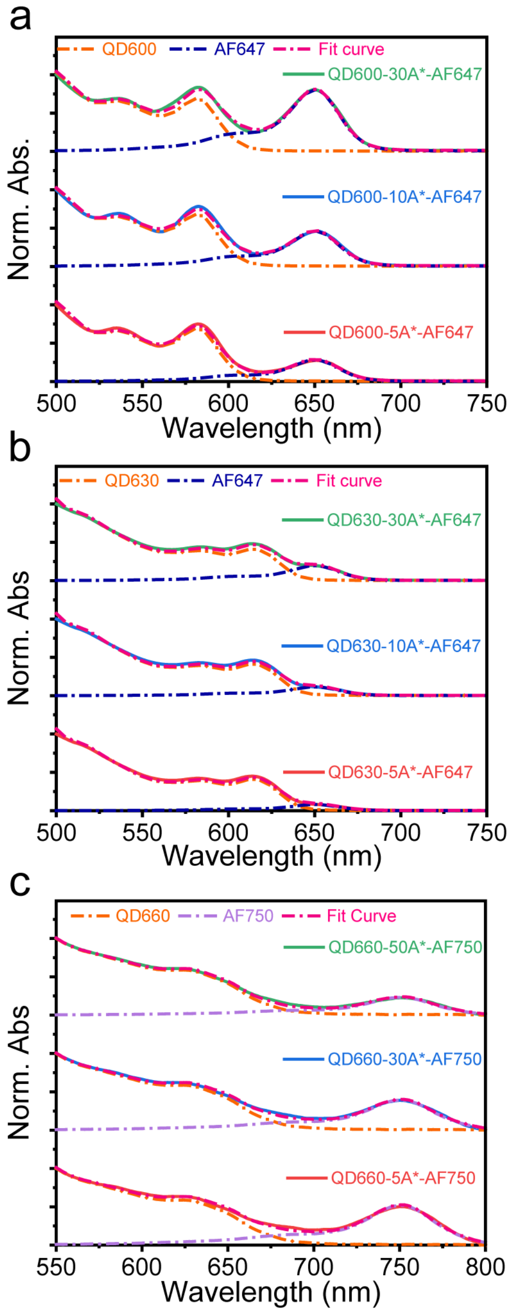

Supplementary Fig. 5. Normalized absorption spectra (solid line) and decomposed spectra (dash-dotted line) of a) QD600-AF647, b) QD630-AF647, and c) QD660-AF750 FRET pairs.

Time-resolved decay curves of QD-dye FRET systems

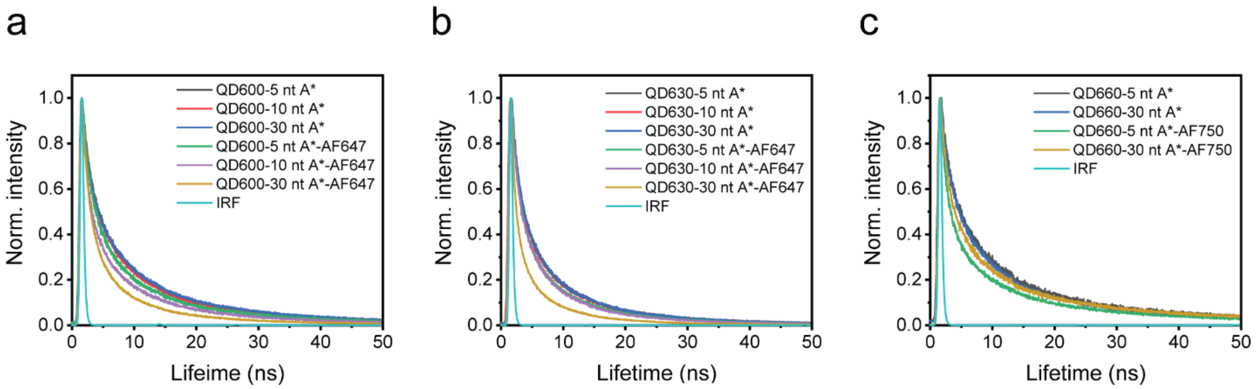

Supplementary Fig. 6. Normalized time-resolved decay curves of a) QD600-AF647, b) QD630-AF647, and c) QD660-AF750 FRET pairs and instrument response function (IRF).

QD wrapped by ssDNA with different ps-backbone length and the fixed po-backbone length

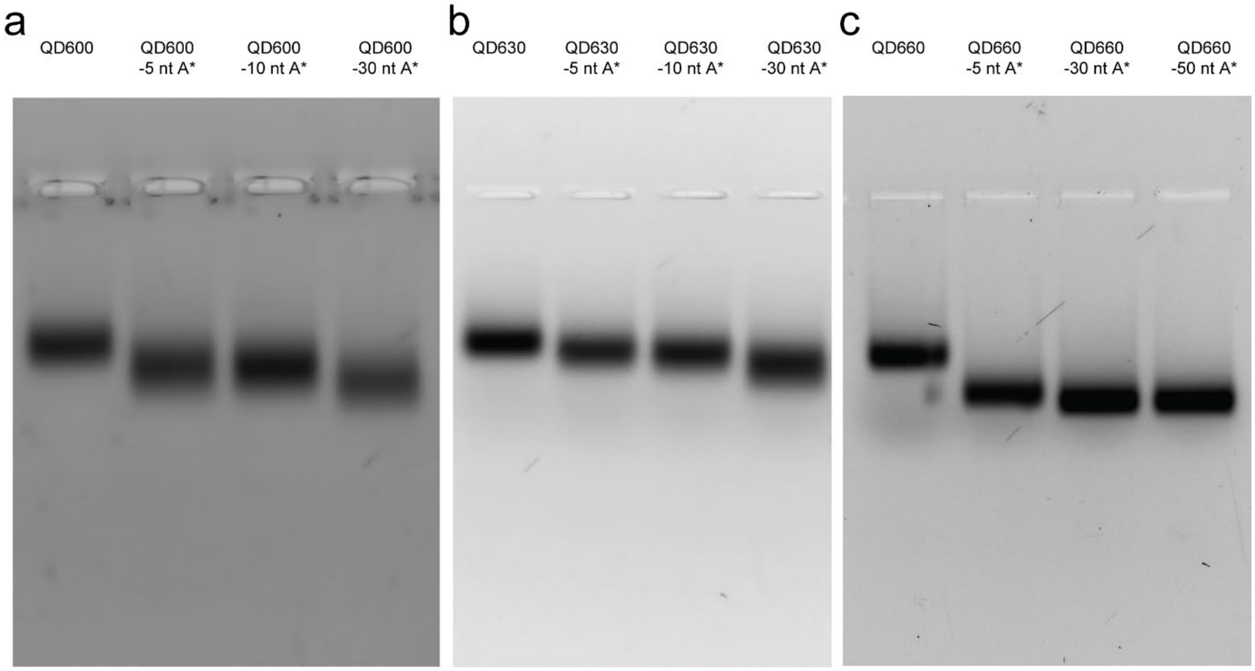

Supplementary Fig. 7. QD wrapped by ssDNA with different ps-backbone length (5, 10, and 30 nt A* for QD600 and QD660, 5, 30, and 50 nt A* for QD660) and the fixed po-backbone length (23 nt). AGE (0.8%) images of a) QD600, b) QD630, and c) QD660.

QD-dye FRET in a distal configuration

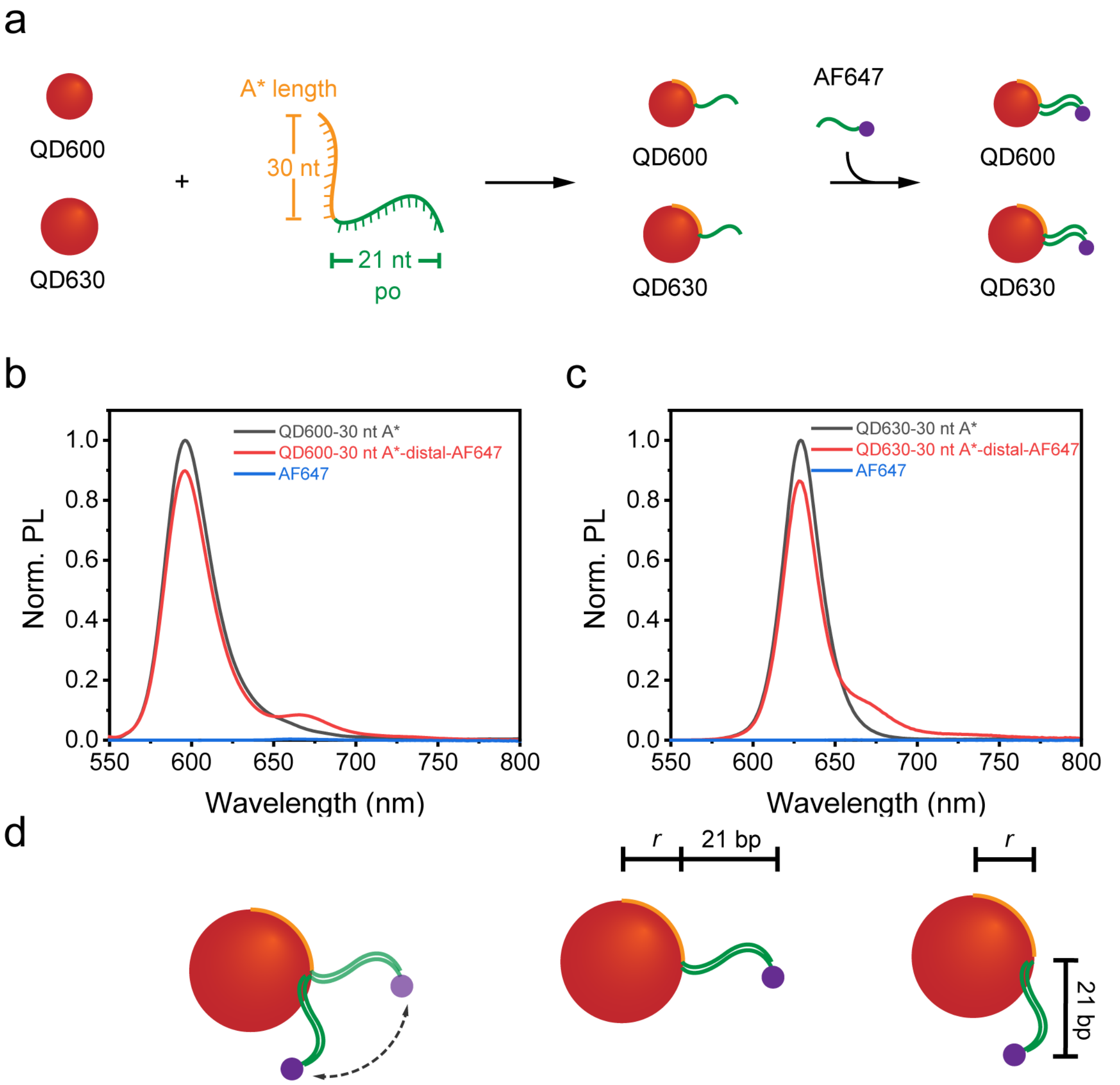

Supplementary Fig. 8. a) Schematic of ssDNA containing 30 nt A* and fixed po domain (21 nt) wrapping on QD600 and QD630, and hybridization with AF647-labelled complementary strand. Photoluminescence (PL) spectra of b) QD600-distal-AF647 and c) QD630-distal-AF647 FRET pairs. FRET efficiencies were 10 ± 2 % and 13 ± 2 %, and donor-acceptor distances increased to 8.3 ± 0.3 and 8.8 ± 0.2 nm (mean ± standard deviation; n = 3) for QD600-distal-AF647 and QD630-distal AF647 FRET pairs, respectively. d) Possible structures of DNA duplex wrapping on the QD and corresponding donor-acceptor distances (7.8-10.4 nm and 8.2-11.2 nm for QD600-distal-AF647 and QD630-distal-AF647, respectively).

QD600 wrapped by ssDNA with various ps-backbone length and the fixed total length

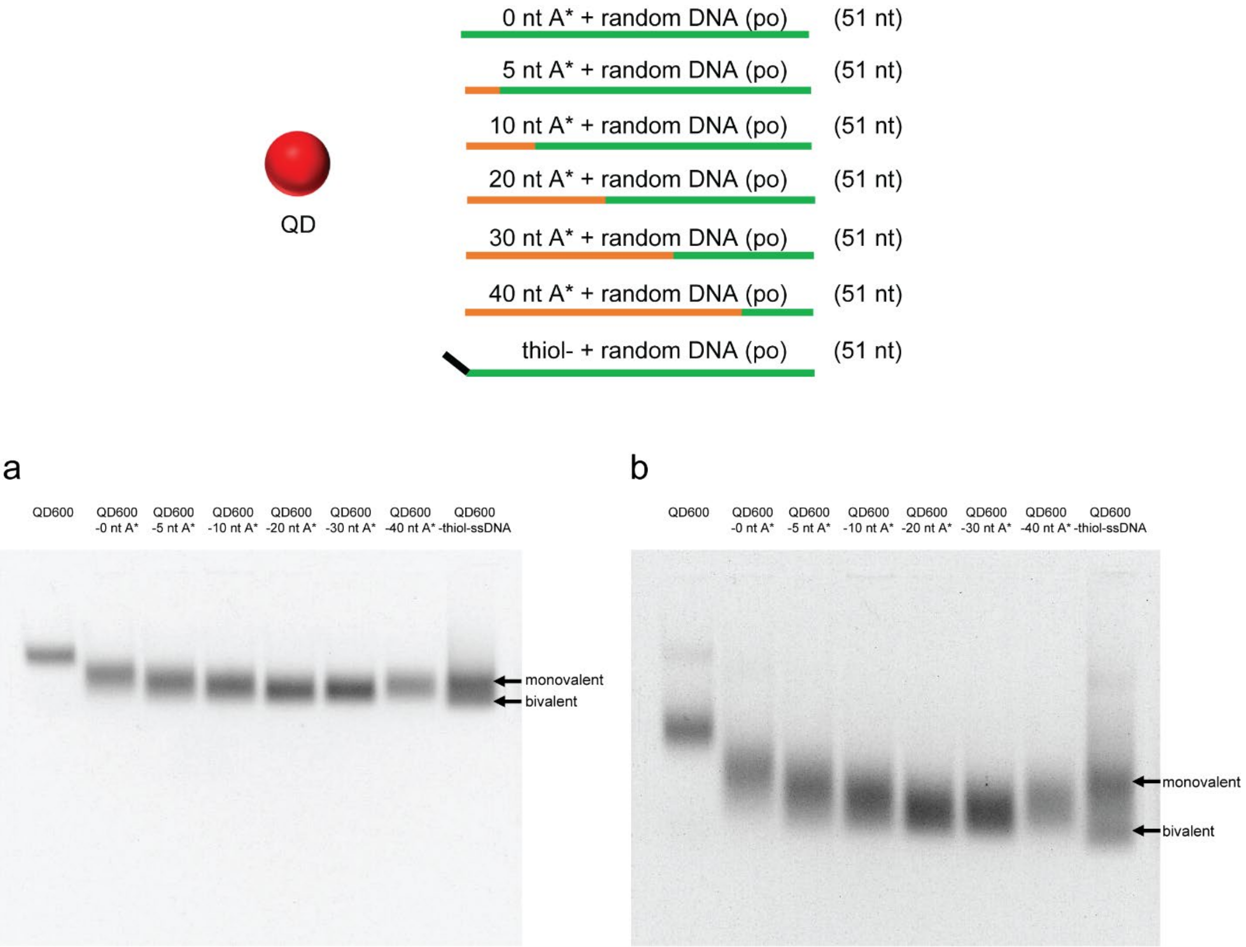

Supplementary Fig. 9. QD wrapped by ssDNA with various ps-backbone length (5, 10, 20, 30 and 40 nt A*) and the fixed total length (51 nt). AGE (0.8%) images of QD600 wrapped by ssDNA in a) 40 min and b) 80 min run. The different mobility of monovalent band of each sample was caused by the different conformations of ssDNA wrapped QD (Supplementary Fig. 11). Source data are provided as Source Data file.

The A* tract can effectively prevent nonspecific DNA adsorption

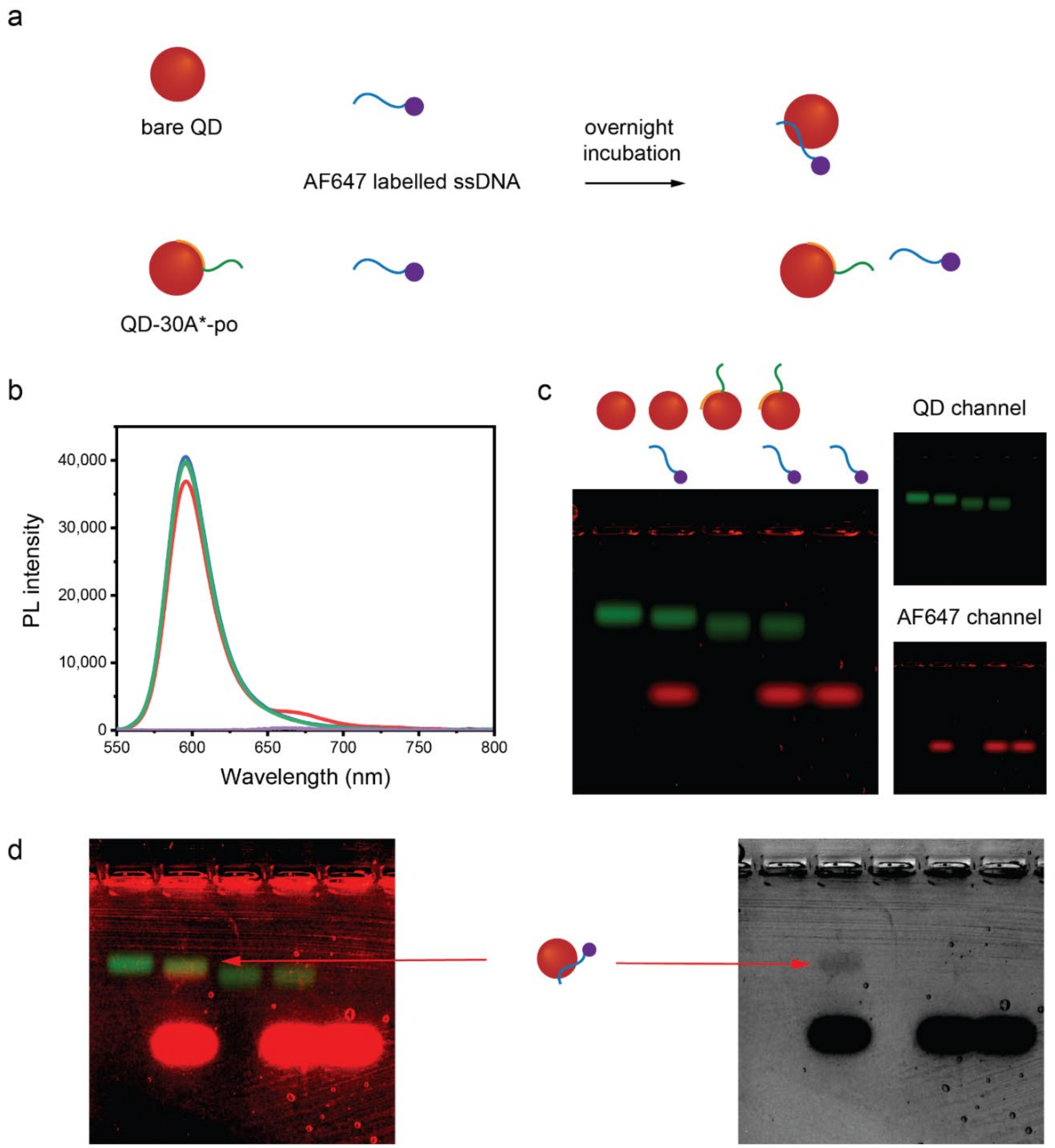

Supplementary Fig. 10. a) Schematic of nonspecific DNA adsorption. b) Steady-state PL spectra and c) AGE (0.8%) images of QD600 alone (black), QD 600 and AF647-21 nt po mixture (red), QD600-30 nt A* alone (blue), QD600-30 nt A* (non-complementary po domain) and AF647-21 nt po mixture (green), and AF647-21 nt po alone (violet) (from left to right). d) Contrast adjustment of AGE images in AF647 channel indicate the tiny amount of DNA nonspecific adsorption on bare QD. Source data are provided as Source Data file.

MD simulation model for A* tract length effect

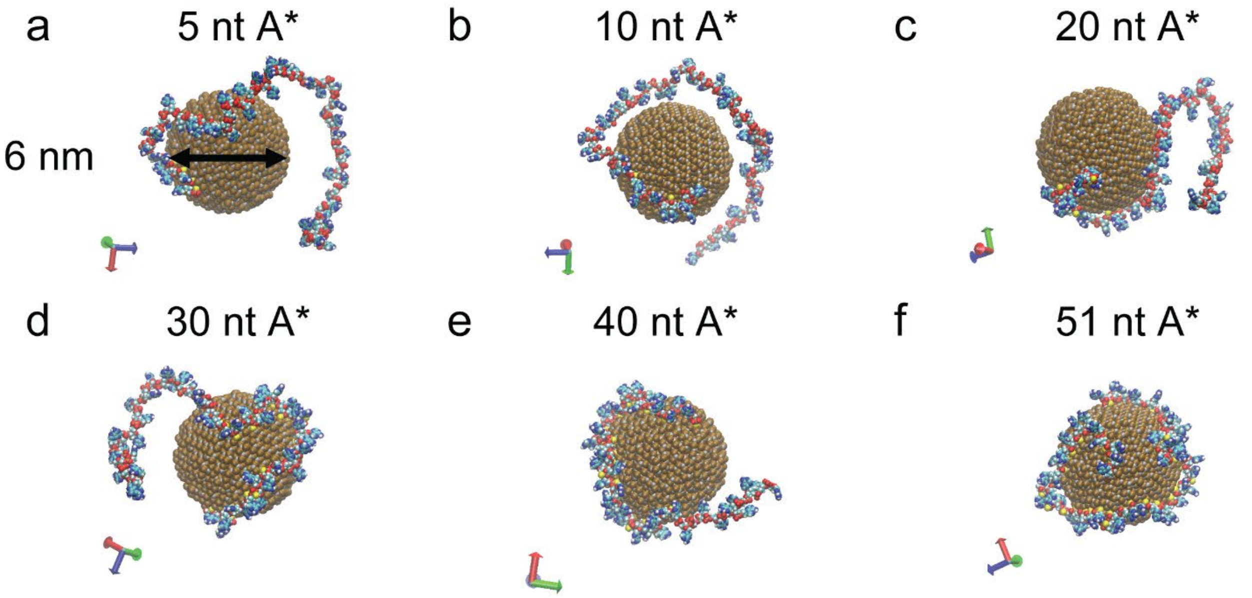

Supplementary Fig. 11. Conformations of ssDNA wrapped ZnS QDs with 6 nm diameter. The ssDNAs have the fixed total length (51 nt) with various ps tract length (5–51 nt A*).

MD simulation model for ssDNA wrapped QD

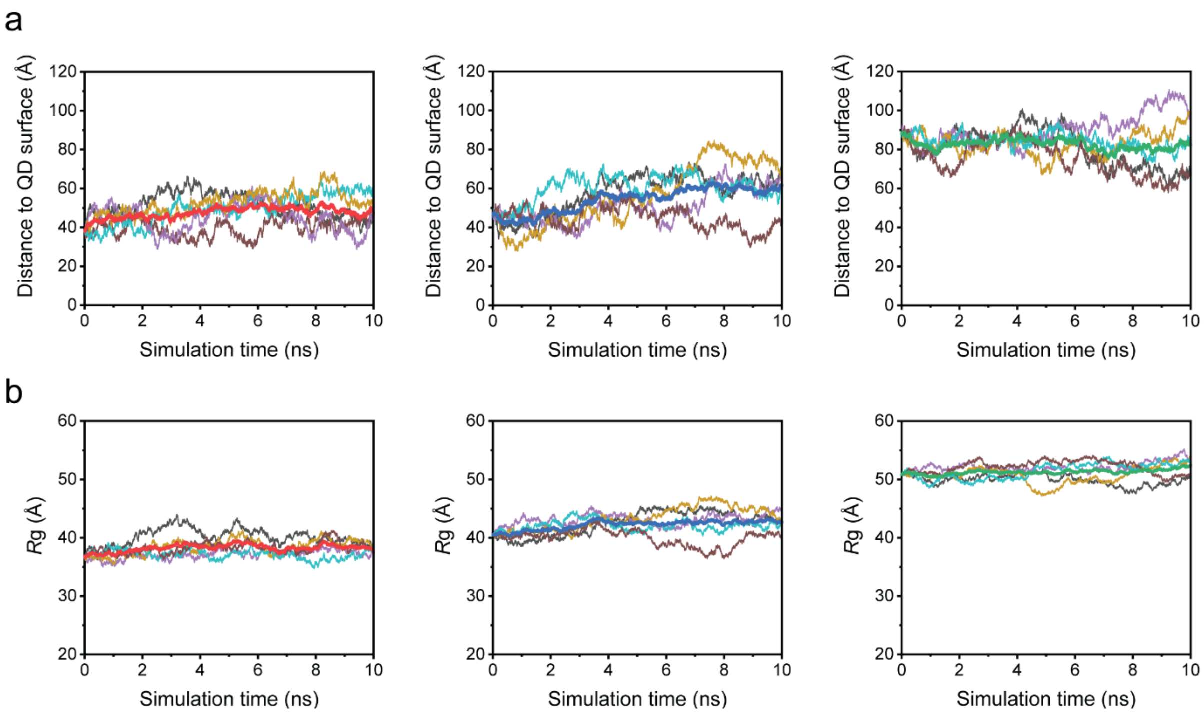

Supplementary Fig. 12. a) The distance of the 5'-end of the po-backbone of (5 nt A*(left), 10 nt A*(middle), and 30 nt A*(right)) + po (23 nt) to the QD surface observed in five 10 ns simulated trajectories. b) Radius of gyration (*R*g) analysis of the po domain along the same trajectories. (Five independent trajectories (thin lines) were propagated from an equilibrated structure using 5 different random seeds for the stochastic solvent response, and the time-dependent average (thick line) is also shown here). Source data are provided as Source Data file.

TEM images of QD dimer

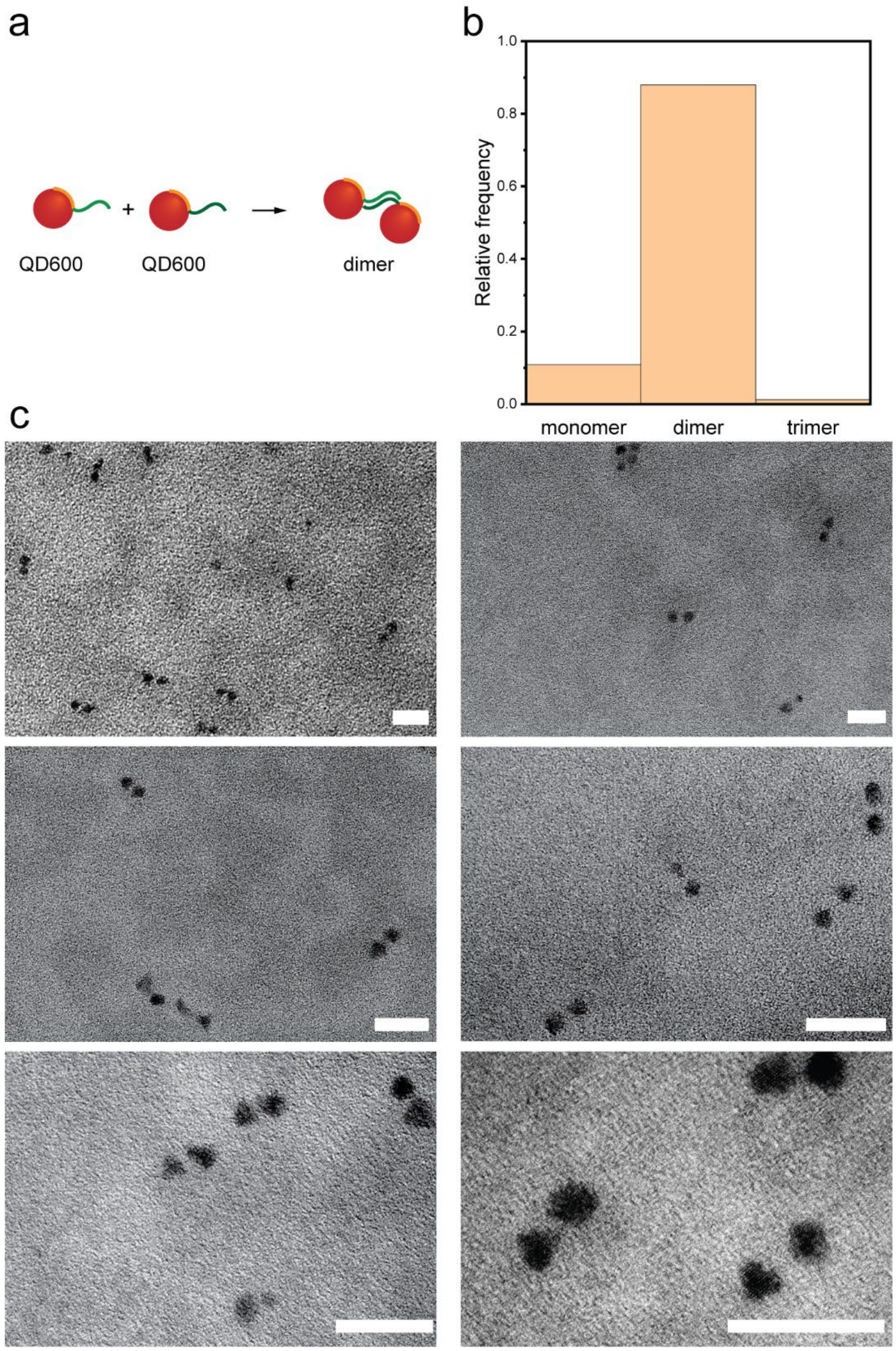

Supplementary Fig. 13. a) Schematic of preparation for QD dimers using monovalent QDs. b) The yield of QD600 dimers calculated from TEM images (166 NPs). c) TEM images of QD dimer at several magnifications (Scale bar: 20 nm). Source data are provided as Source Data file.

QD600 wrapped by ssDNA with various ps-backbone length and the fixed total length

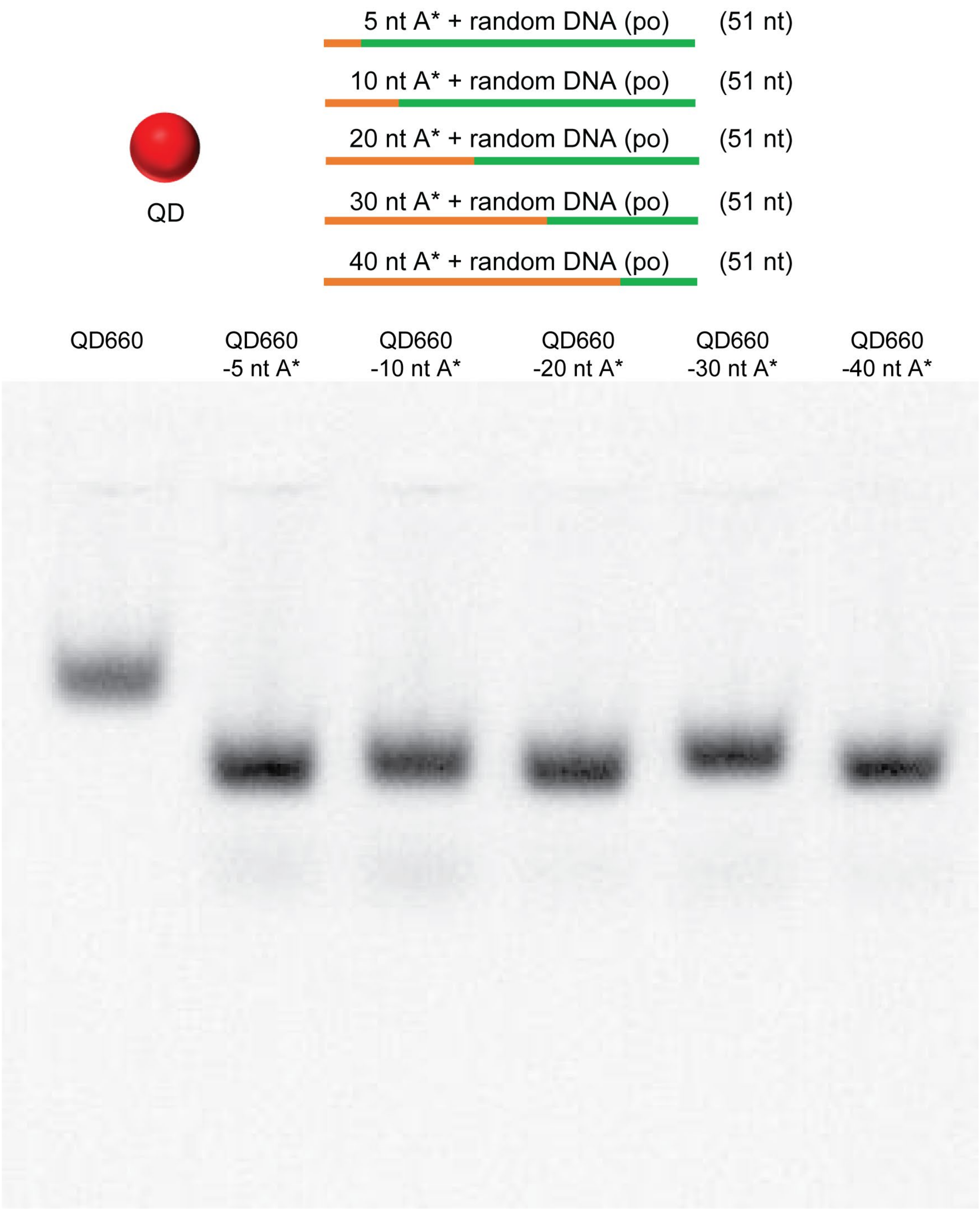

Supplementary Fig. 14. QD660 wrapped by ssDNA with various A* tract length (5, 10, 20, 30 and 40 nt A*) and the fixed total length (51 nt). AGE (0.8%) images of QD660 wrapped by ssDNA.

MD simulation model for the effect of QD size

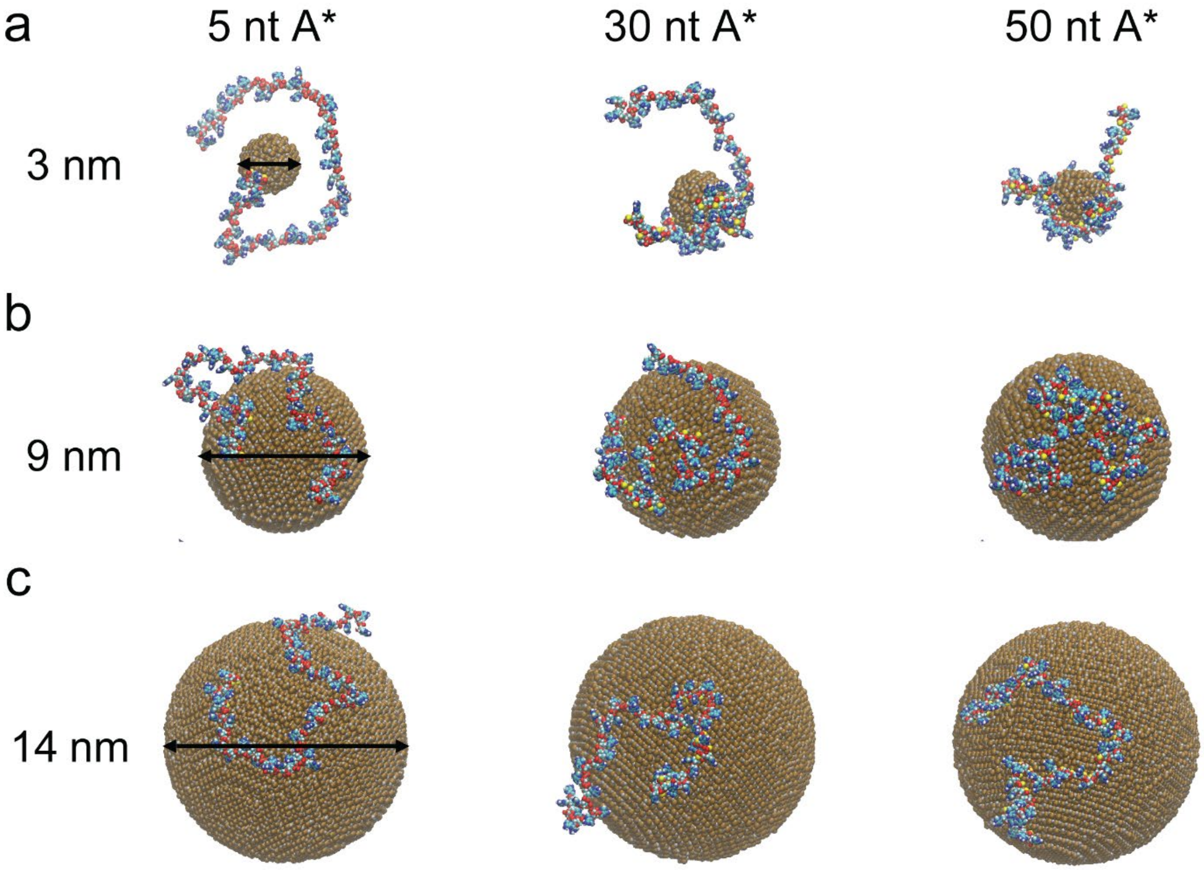

Supplementary Fig. 15. Representative conformations of ssDNA wrapped ZnS QDs, as seen in the simulated trajectories, with a) 3 nm, b) 9 nm, and c) 14 nm diameter. The fixed total length (51 nt) with various ps-backbone length (5, 30, and 50 nt A*). Larger size QDs can increase the probability of attaching more than one ssDNA.

TEM images of commercially-available streptavidin QD.

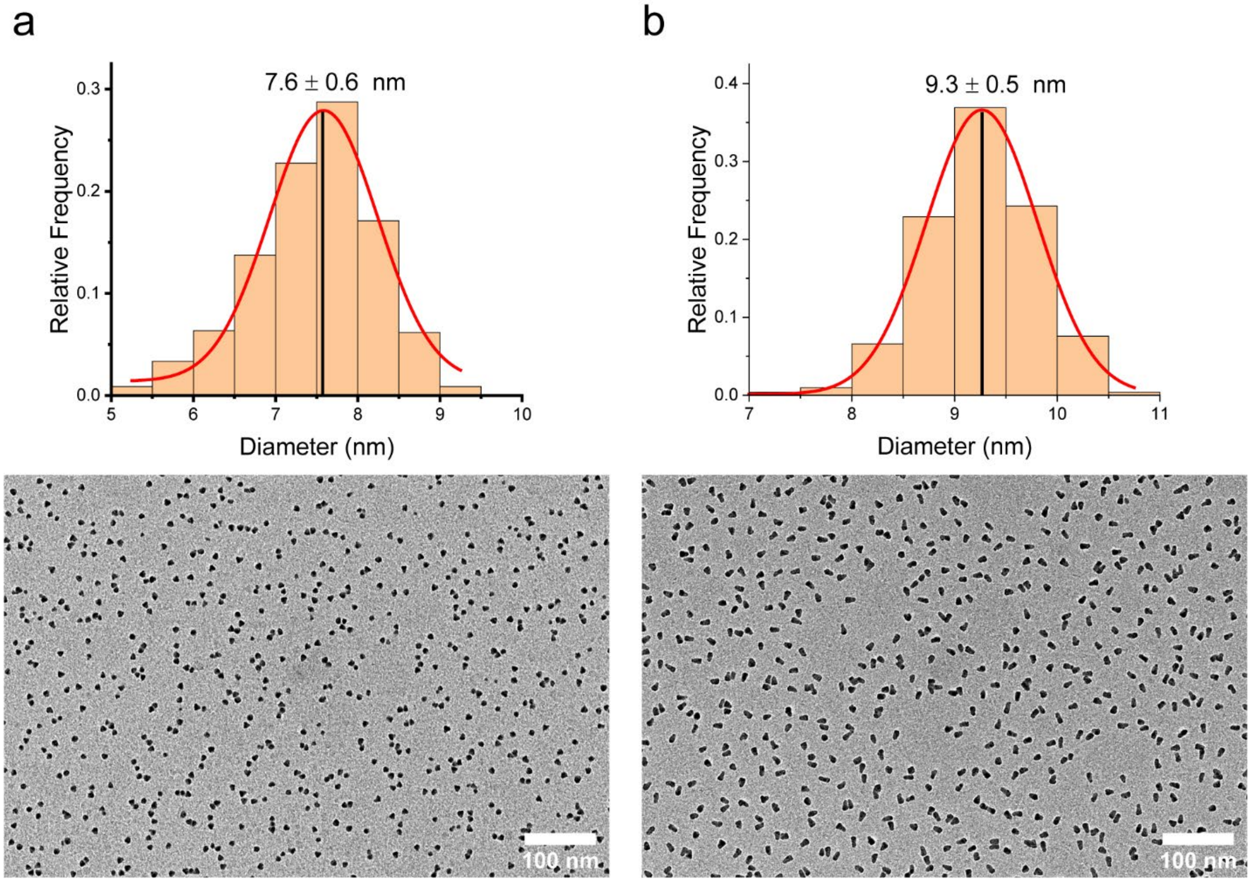

Supplementary Fig. 16. TEM images (bottom) and size distribution analysis (top) for commercial a) streptavidin QD605 and b) streptavidin QD655. Particle diameters were calculated from 567 NPs (a) and 515 NPs (b), respectively. Red curves are Gaussian fit to the measured particle diameter distribution. Indicated particle diameters for each histogram are expressed as mean ± standard deviation. Source data are provided as Source Data file.

Streptavidin QD-dye FRET system

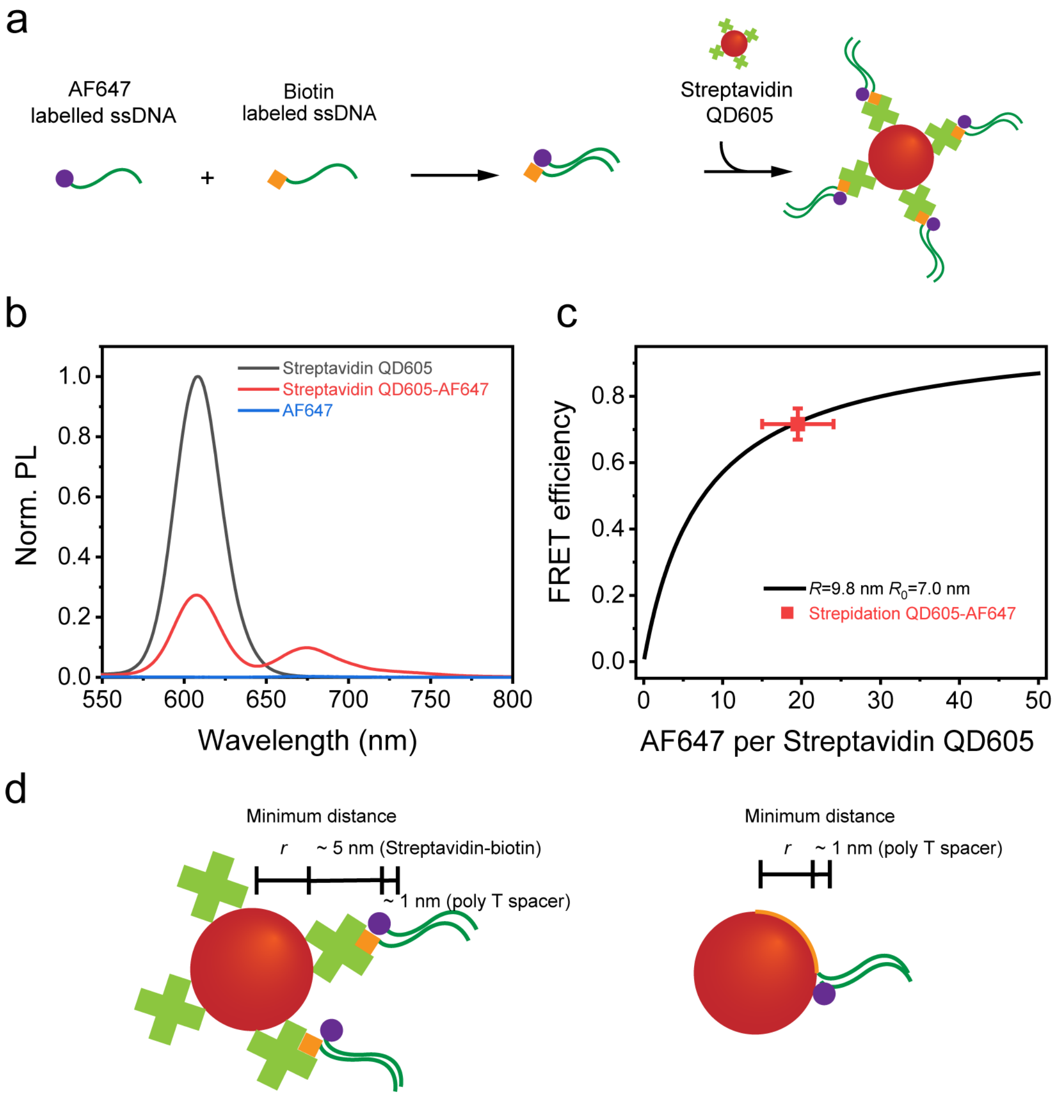

Supplementary Fig. 17. a) Schematic of streptavidin QD605 binding with biotin-modified AF647- labelled DNA duplex. b) PL spectra of streptavidin QD605 -AF 647 FRET pairs. c) FRET efficiencies as a function of acceptors calculated theoretically (black curves, Eq. (5)), and from QD emission intensities (Supplementary Fig. 17b). The donor-acceptor distances of 9.8 nm were determined by radius of streptavidin QD605 (~3.8 nm, Supplementary Fig. 16a), streptavidin-biotin conjugation (~5 nm), and polyT spacer (~1 nm). Error bars represent standard deviation of the mean (n = 3 replicates per group). d) Schematic for minimum distance calculation of streptavidin QD-dye FRET system (left) and monovalent wrapping QD-dye FRET system (right). Source data are provided as Source Data file.

AGE images of Pep wireframe with biotin domain and ps-backbone wrapping domain

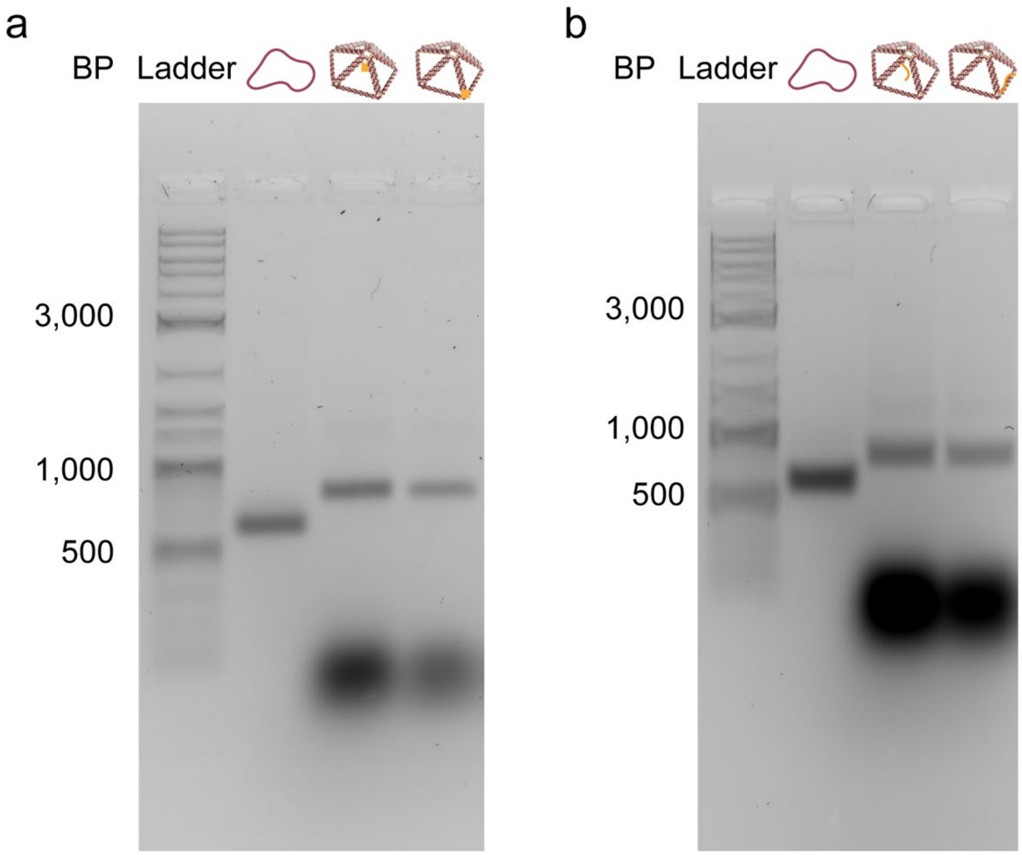

Supplementary Fig. 18. AGE (1.5%) images of a) DNA ladder, scaffold, Pep wireframe origami objects with biotin domain at the inner center and outer edge, and b) DNA ladder, scaffold, Pep wireframe origami objects with 30 nt A* ssDNA wrapping domain at the inner center and outer edge (from left to right). The very bright bands visible below 500 bp are the excess of staple strands visible before purification.

AGE images of conjugation of streptavidin QD655 and Pep wireframe DNA origami objects with biotin domain

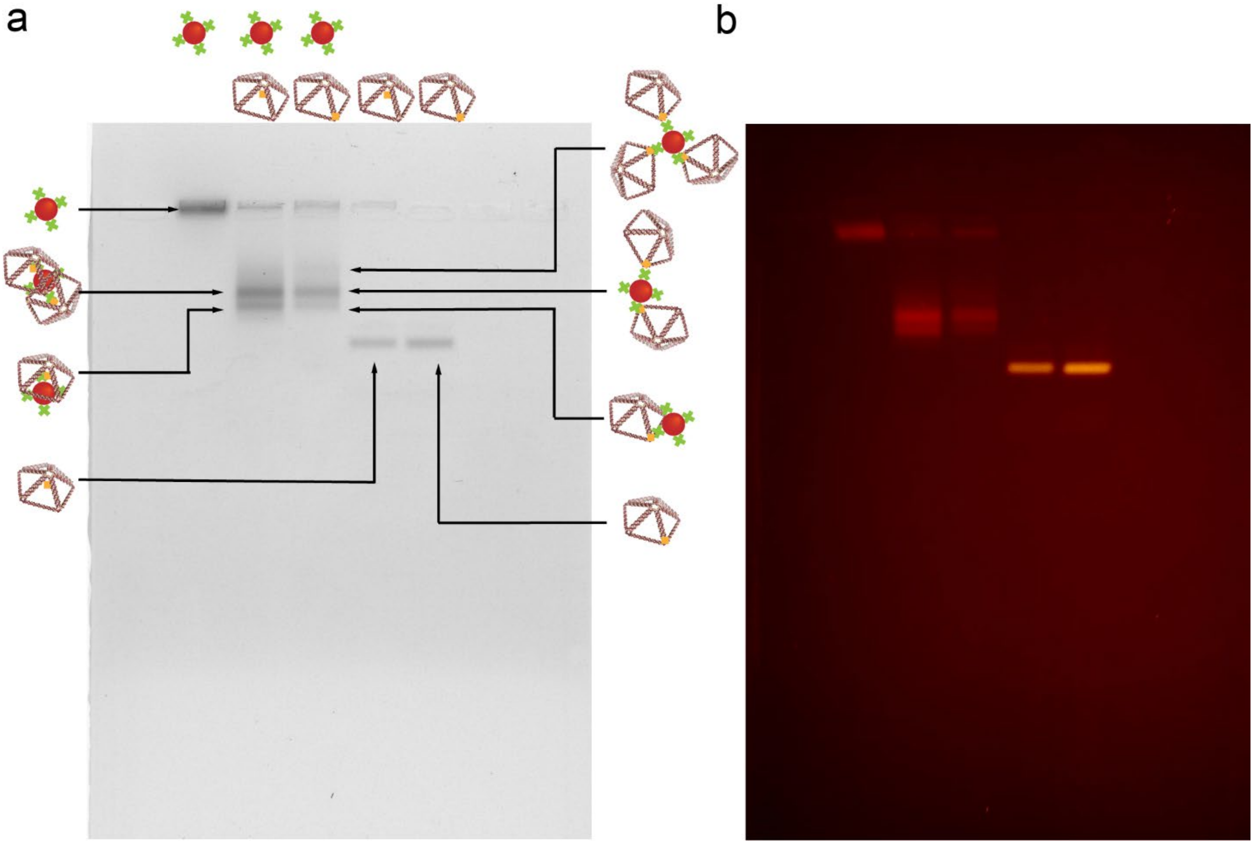

Supplementary Fig. 19. AGE (0.8 %) images of streptavidin QD655 lone, Pep-biotin-streptavidin QD655 assemblies with the biotin at the inner center, Pep-biotin-streptavidin QD655 assemblies with the biotin at the outer edge, Pep-biotin (inner center), and Pep-biotin (outer edge) (from left to right) in 2 h run. Fluorescence images taken by a) gel imaging system and b) digital camera under blue light excitation with ten times Pep alone control.

TEM images of Pep-30 nt A*-QD630 assemblies using ps-backbone based wrapping domain at the inner center.

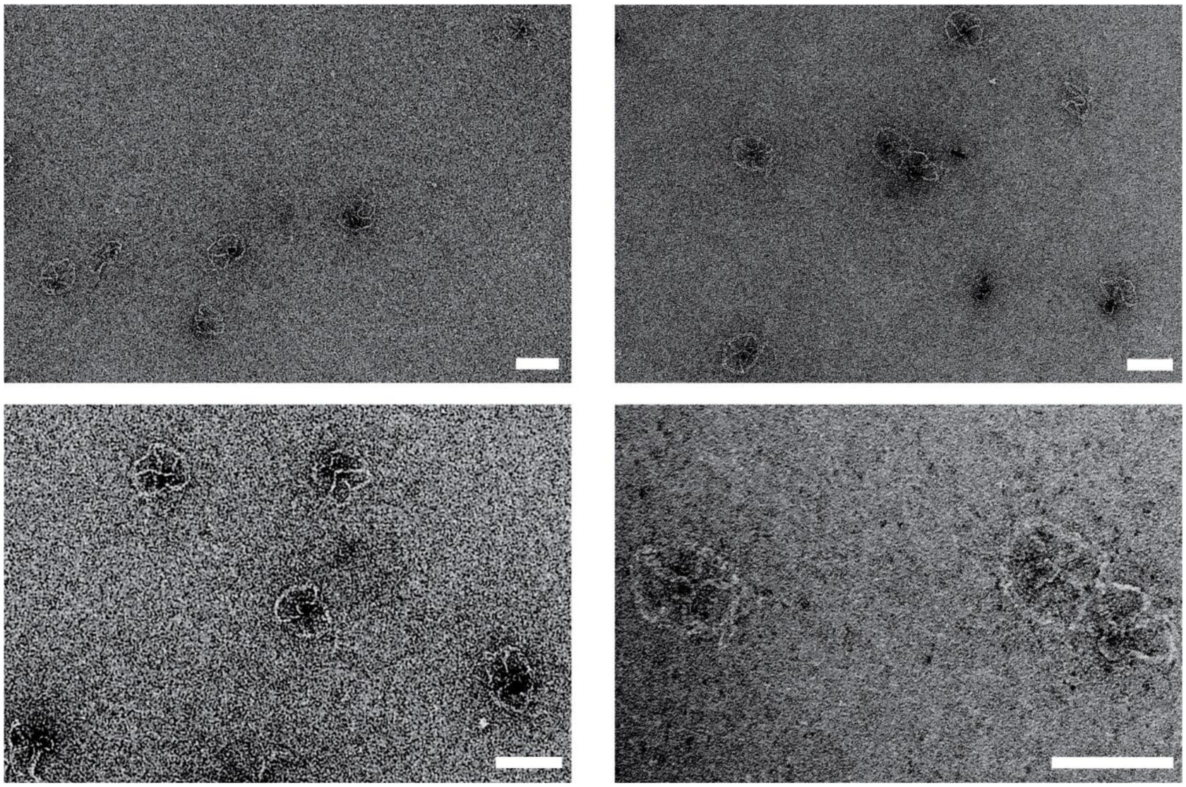

Supplementary Fig. 20. TEM images of Pep-30 nt A*-QD630 assemblies using ps-backbone based wrapping domain at the inner center at several magnifications. (Scale bars: 50 nm)

TEM images of Pep-30 nt A*-QD630 assemblies using ps-backbone based wrapping domain at the outer edge.

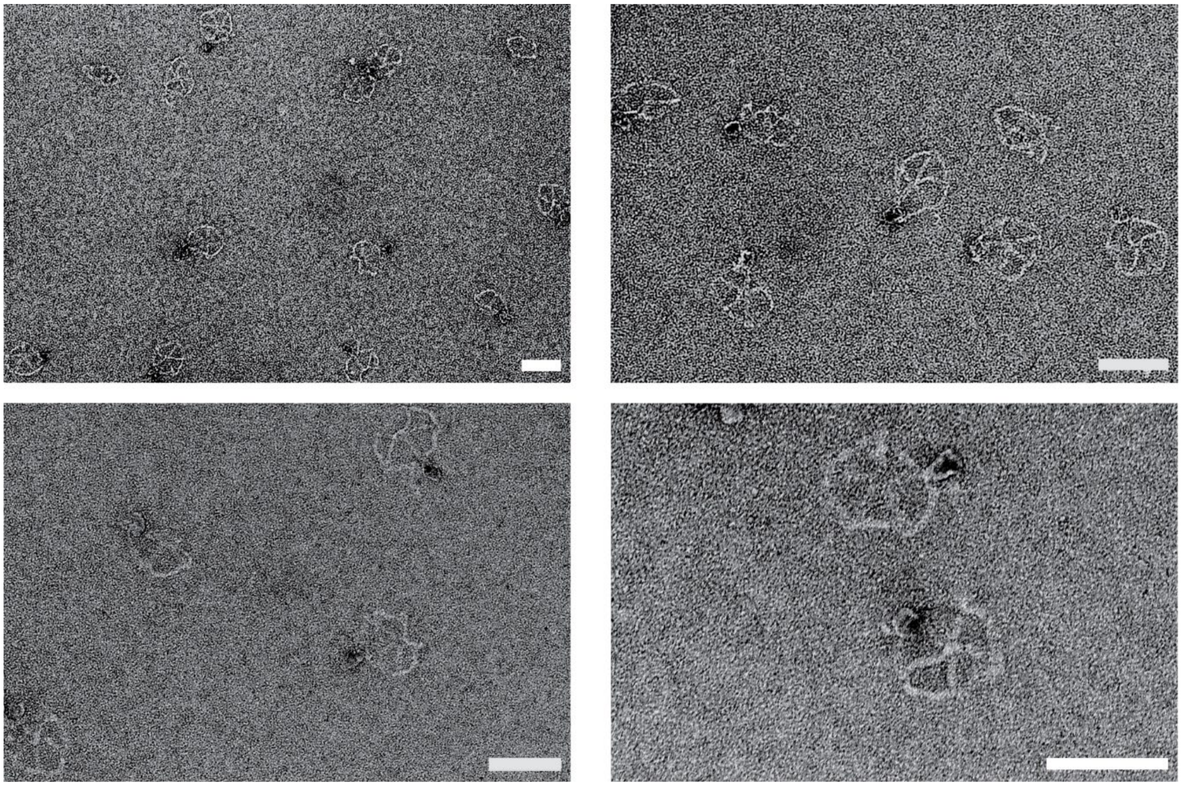

Supplementary Fig. 21. TEM images of Pep-30 nt A*-QD630 assemblies using ps-backbone based wrapping domain at the outer edge at several magnifications. (Scale bars: 50 nm)

TEM images of Pep-biotin-streptavidin QD655 assemblies using biotin domain at the inner center.

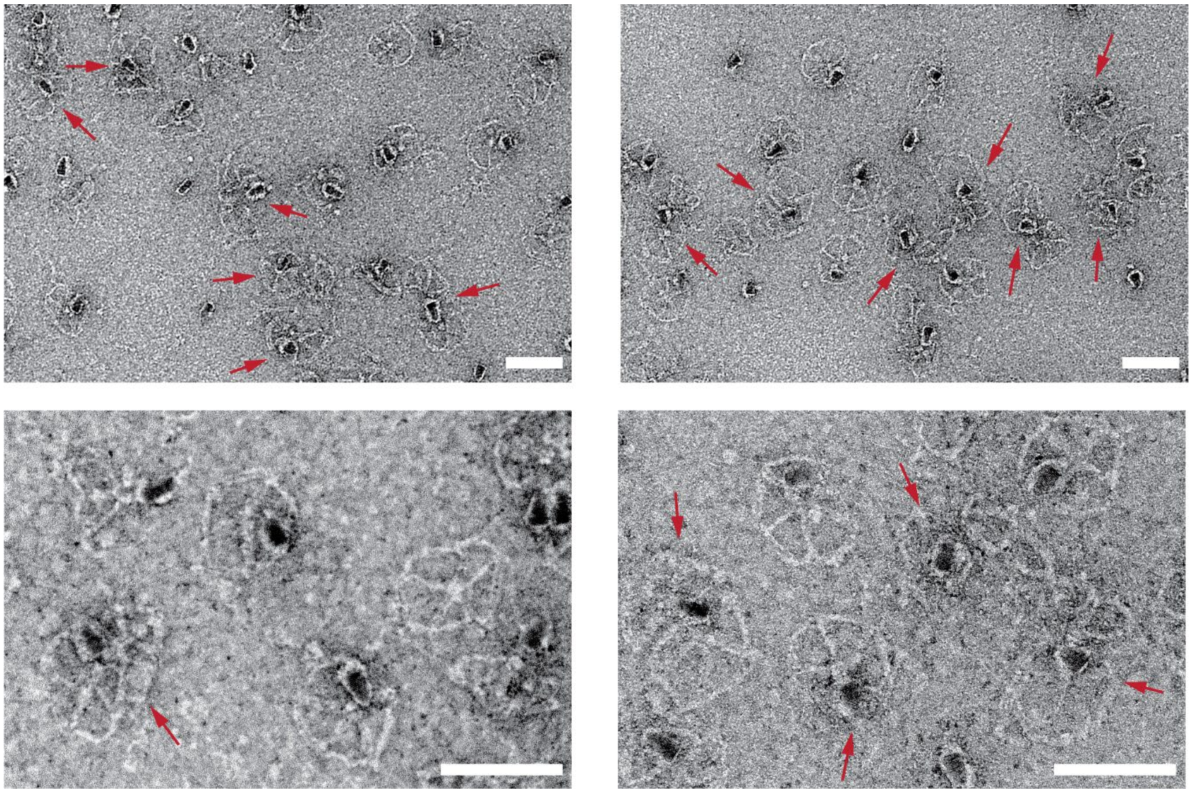

Supplementary Fig. 22. TEM images of Pep-biotin-streptavidin QD655 assemblies using biotin domain at the inner center at several magnifications. (Red arrow: divalent assemblies. Scale bars: 50 nm)

TEM images of Pep-biotin-streptavidin QD655 assemblies using biotin domain at the outer edge.

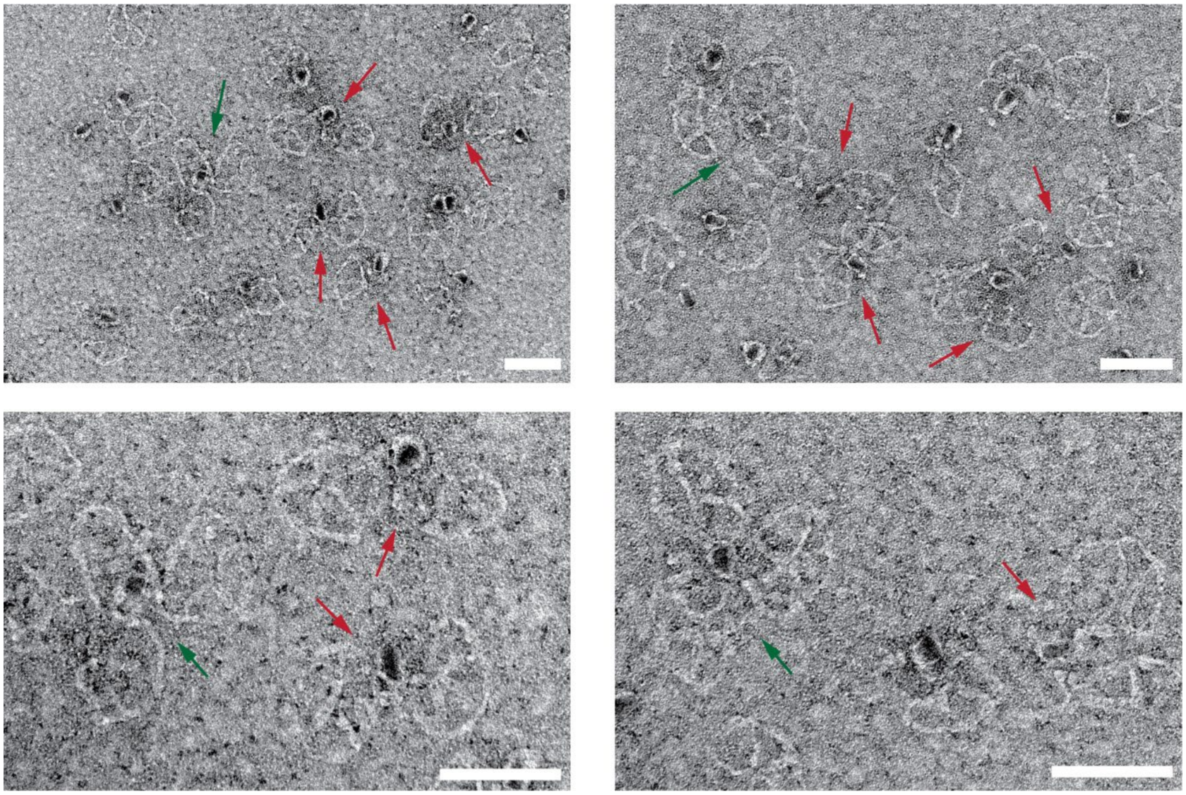

Supplementary Fig. 23. TEM images of Pep-biotin-streptavidin QD655 assemblies using biotin domain at the outer edge at several magnifications. (Red arrow: divalent assemblies, green arrow: trivalent assemblies. Scale bars: 50 nm)

The yield of Pep-QD assemblies using ssDNA wrapping and biotin-streptavidin conjugation

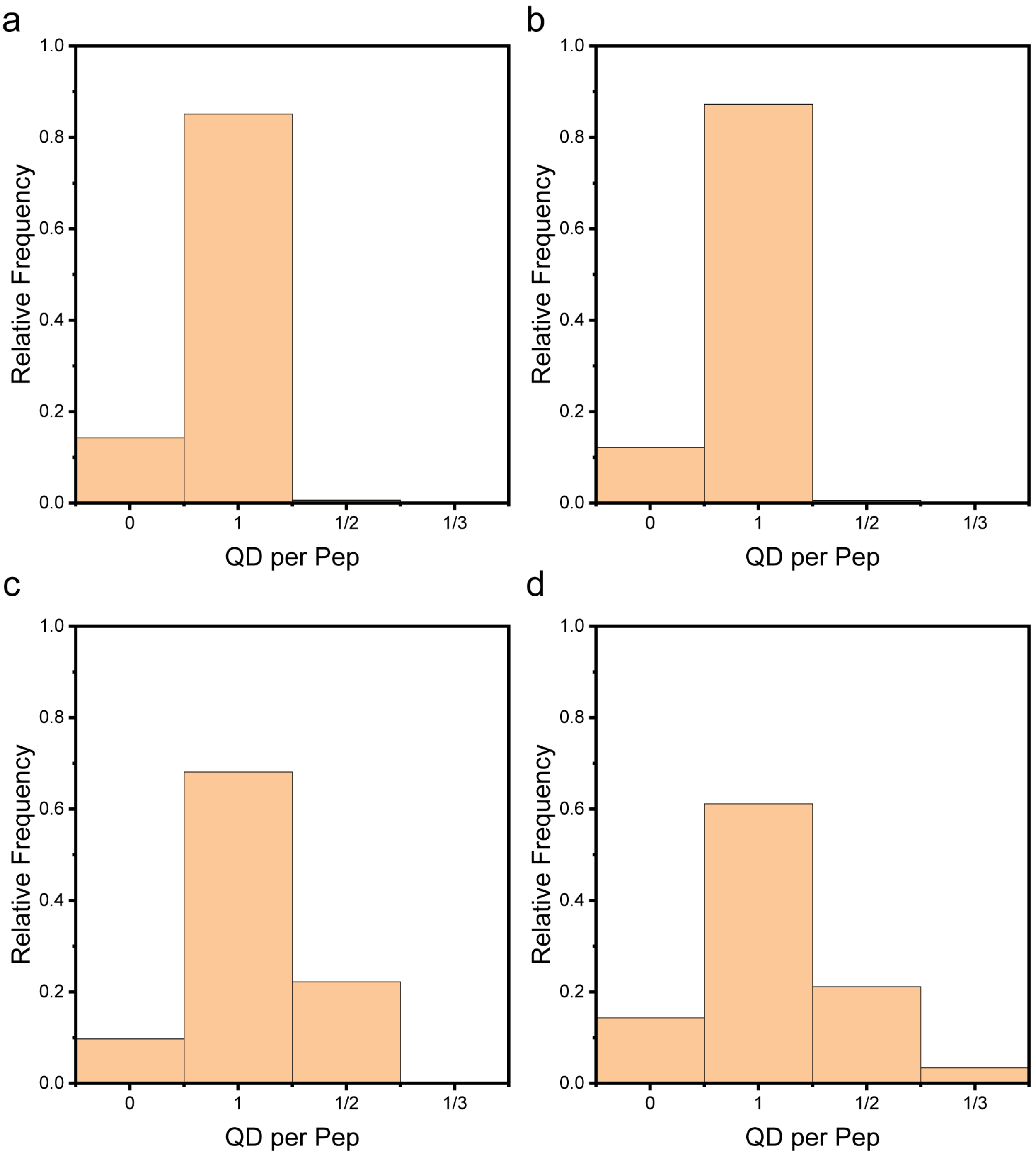

Supplementary Fig. 24. The yield of Pep-30 nt A*-QD630 assemblies using ps-backbone wrapping domain at the a) inner center and b) outer edge. The yield of Pep-biotin-streptavidin QD655 assemblies using biotin domain at the c) inner center and d) outer edge. Yields were calculated from TEM images (308 assemblies for a, 337 assemblies for b 257 assemblies for c), and 265 assemblies for d)). Source data are provided as Source Data file.

Schematic for wireframe DNA origami objects folding and preparation of valence-geocoded QD

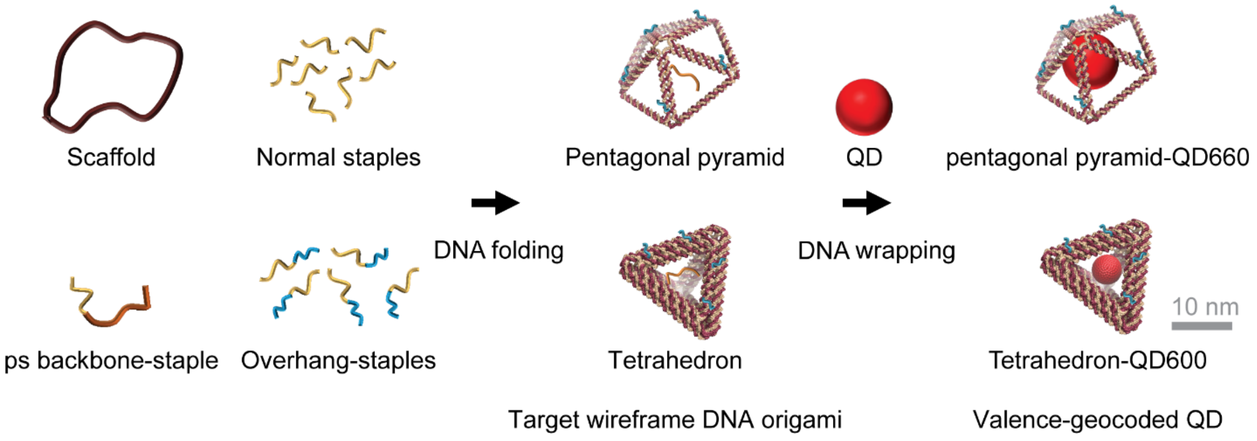

Supplementary Fig. 25. Scaffold is combined with normal staples, ps-backbone-modified staples, and overhang-staples to self-assemble the target wireframe structure using thermal annealing. QDs with various size are added to purified wireframe DNA origami objects with suitable internal cavity dimensions to fabricate valence-geocoded QDs.

AGE image of Tet wireframe DNA origami objects folding

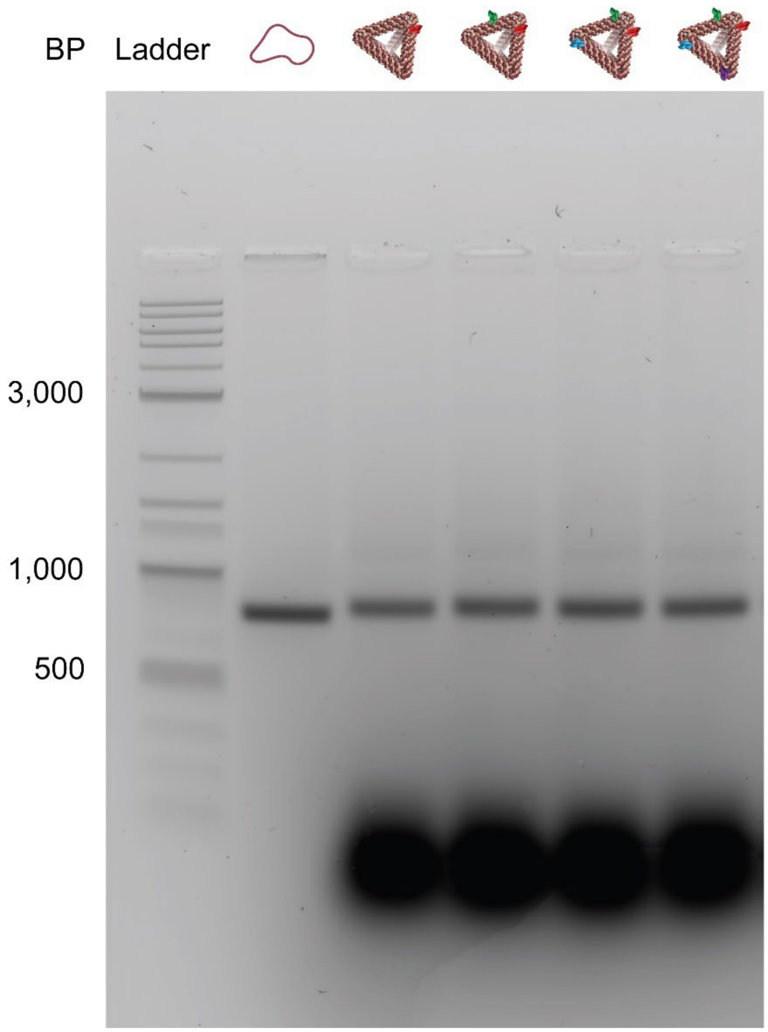

Supplementary Fig. 26. AGE (1.5%) images of DNA ladder, scaffold, Tet wireframe origami objects with 1-4 overhangs. The very bright bands visible below 500 bp are the excess of staple strands visible before purification.

AGE image of Pep wireframe DNA origami objects folding

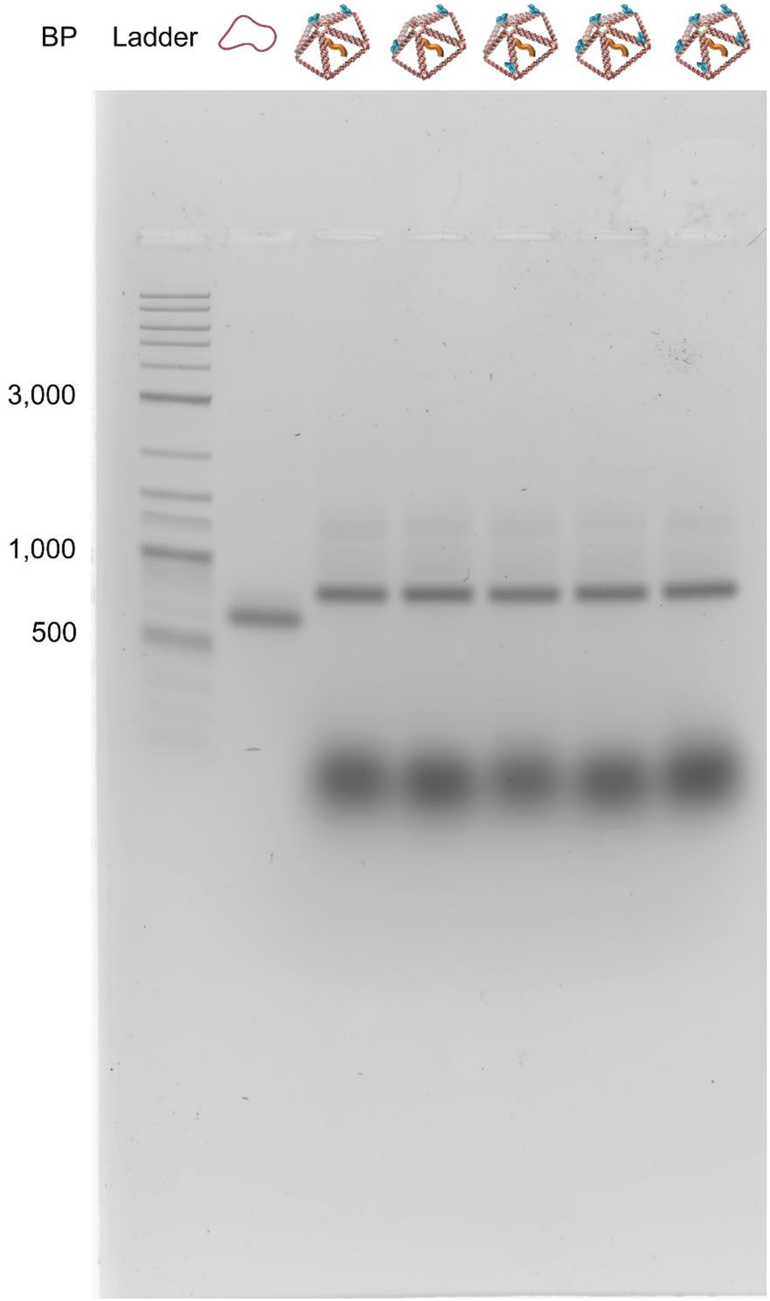

Supplementary Fig. 27. AGE (1.5%) images of DNA ladder, scaffold, Pep trimer Type A wireframe DNA origami objects, Pep trimer Type B wireframe DNA origami objects, Pep tetramer wireframe DNA origami objects, Pep pentamer wireframe DNA origami objects, and Pep hexamer wireframe DNA origami objects. The very bright bands visible below 500 bp are the excess of staple strands visible before purification.

TEM images of Tet wireframe DNA origami objects

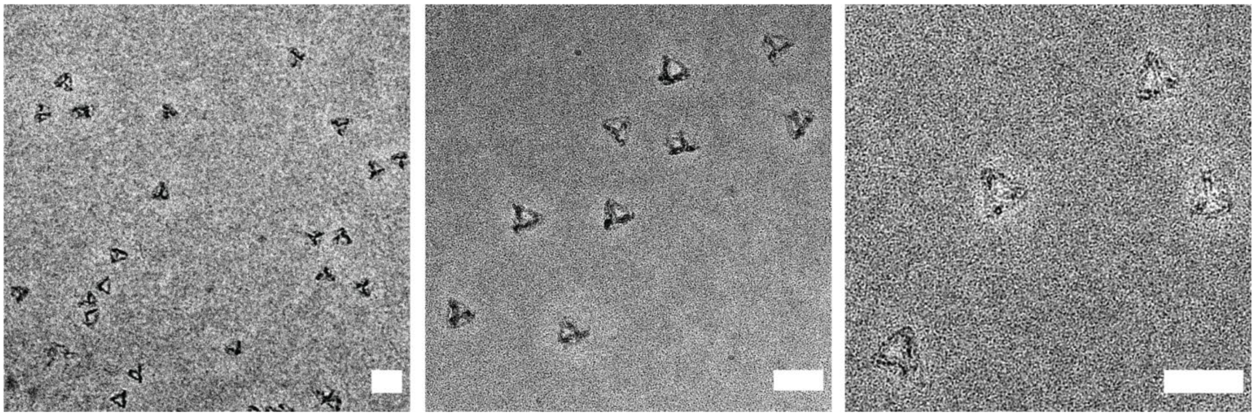

Supplementary Fig. 28. TEM images of Tet wireframe DNA origami objects at several magnifications. The color of Tet wireframe DNA origami objects were inverted for clarity (scale bar: 50 nm).

TEM images of Tet-QD600 assemblies

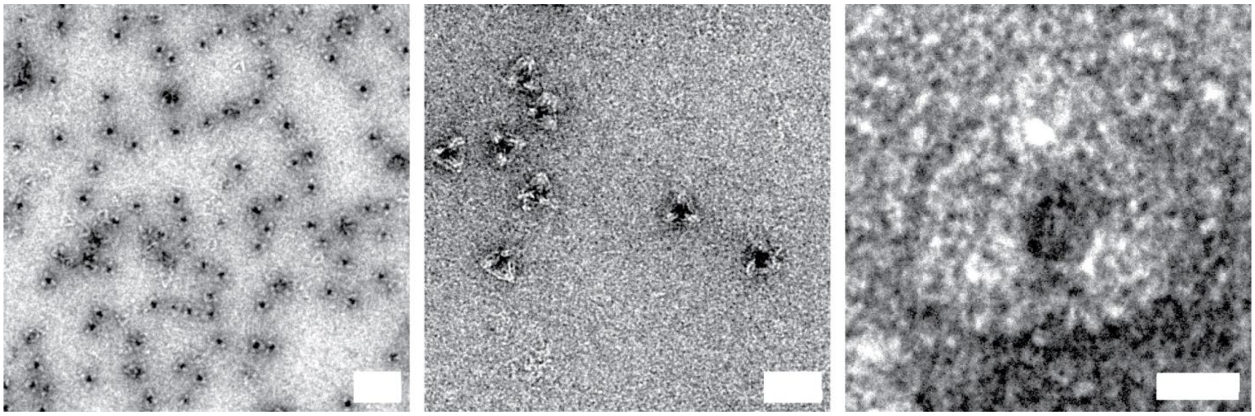

Supplementary Fig. 29. TEM images of Tet-QD600 assemblies at several magnifications. (Scale bars from left to right: 100 nm, 50 nm, and 10 nm)

TEM images of Pep wireframe DNA origami objects

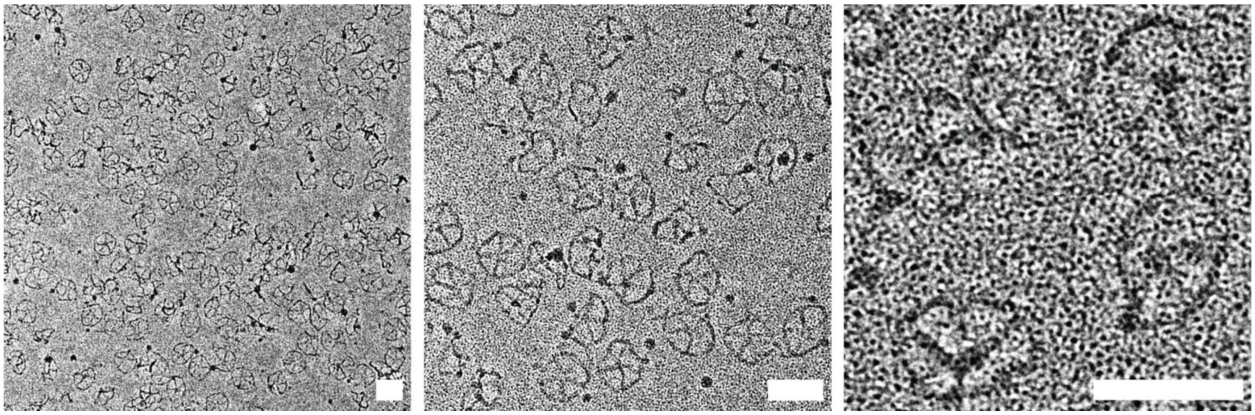

Supplementary Fig. 30. TEM images of Pep wireframe DNA origami objects at several magnifications. The color of Pep wireframe DNA origami objects were inverted for clarity (scale bar: 50 nm).

TEM images of Pep-QD660 assemblies

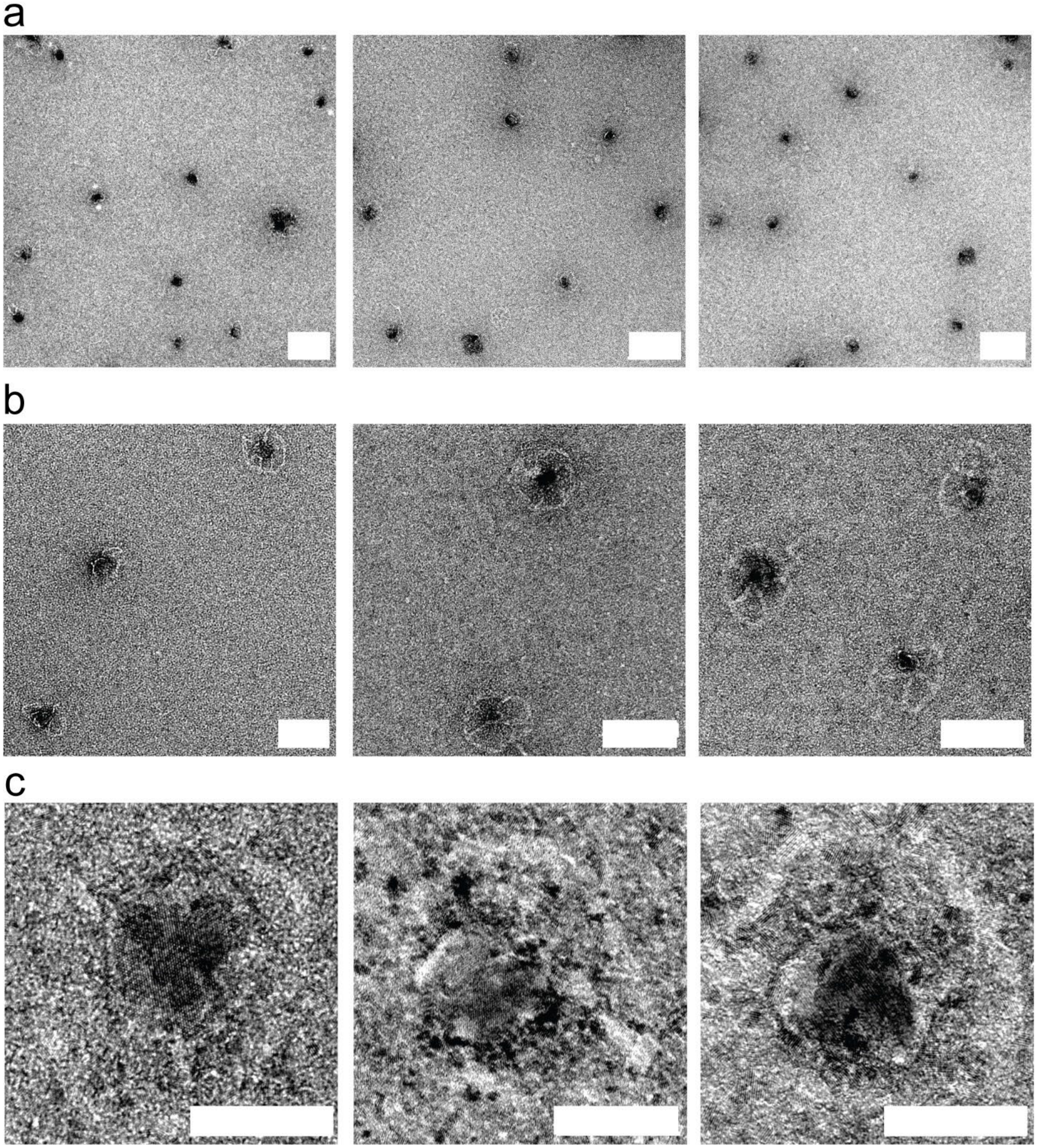

Supplementary Fig. 31. TEM images of Pep-QD660 assemblies at several magnifications. Scale bars: a) 100 nm, b) 50 nm, c) 20 nm.

AGE images of Tet-QD600 assemblies

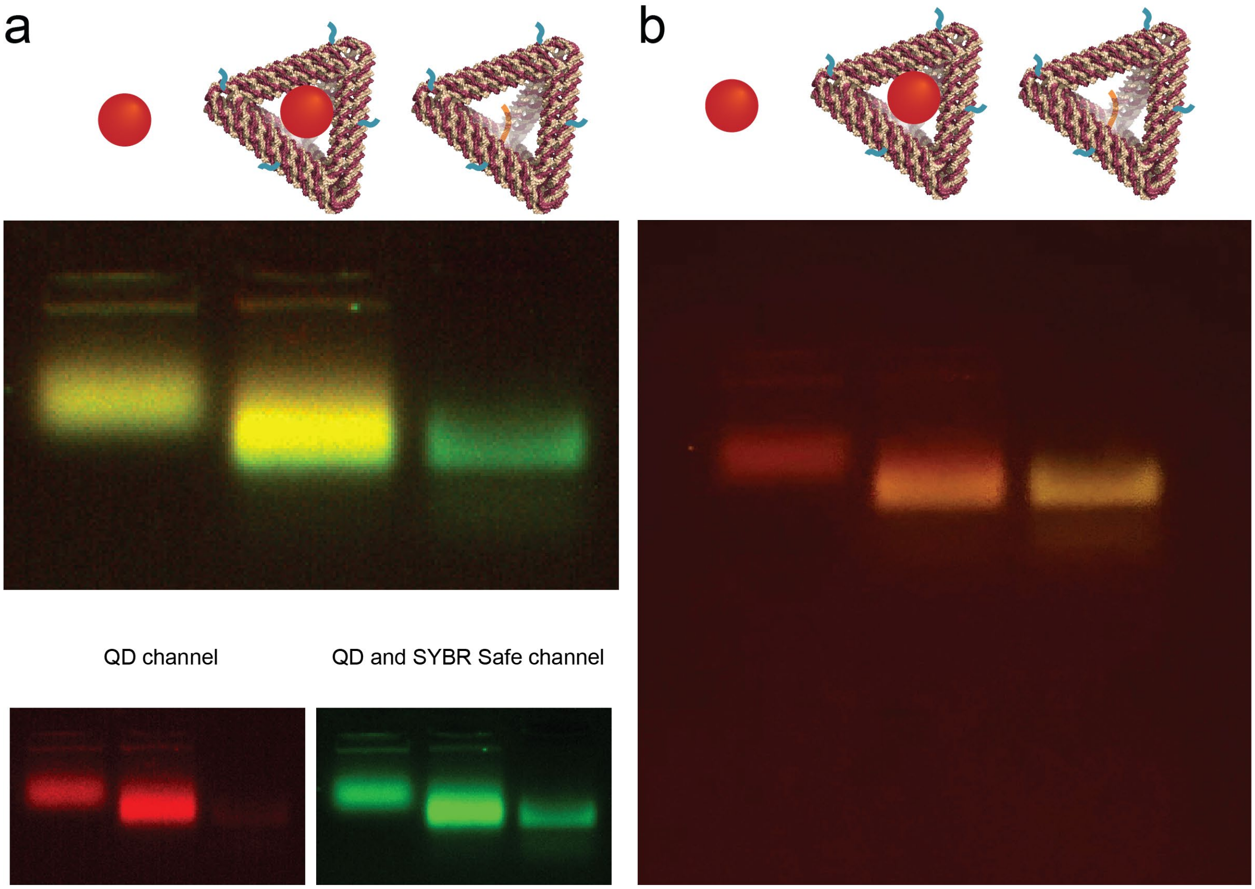

Supplementary Fig. 32. AGE (0.8 %) image of QD600 alone, Tet-QD600 assemblies and Tet wireframe DNA origami objects alone (from left to right). a) Digital images taken by a gel imaging system under UV (QD channel) and blue light (QD and SYBR Safe channel) excitation. b) Image of a blue-light illuminated gel taken using a digital camera. Although the QD600 and SYBR Safe (wireframe DNA origami objects) have similar emission regions, we can distinguish them using the digital camera (iPhone X). Source data are provided as Source Data file.

AGE images of Pep-QD660 assemblies

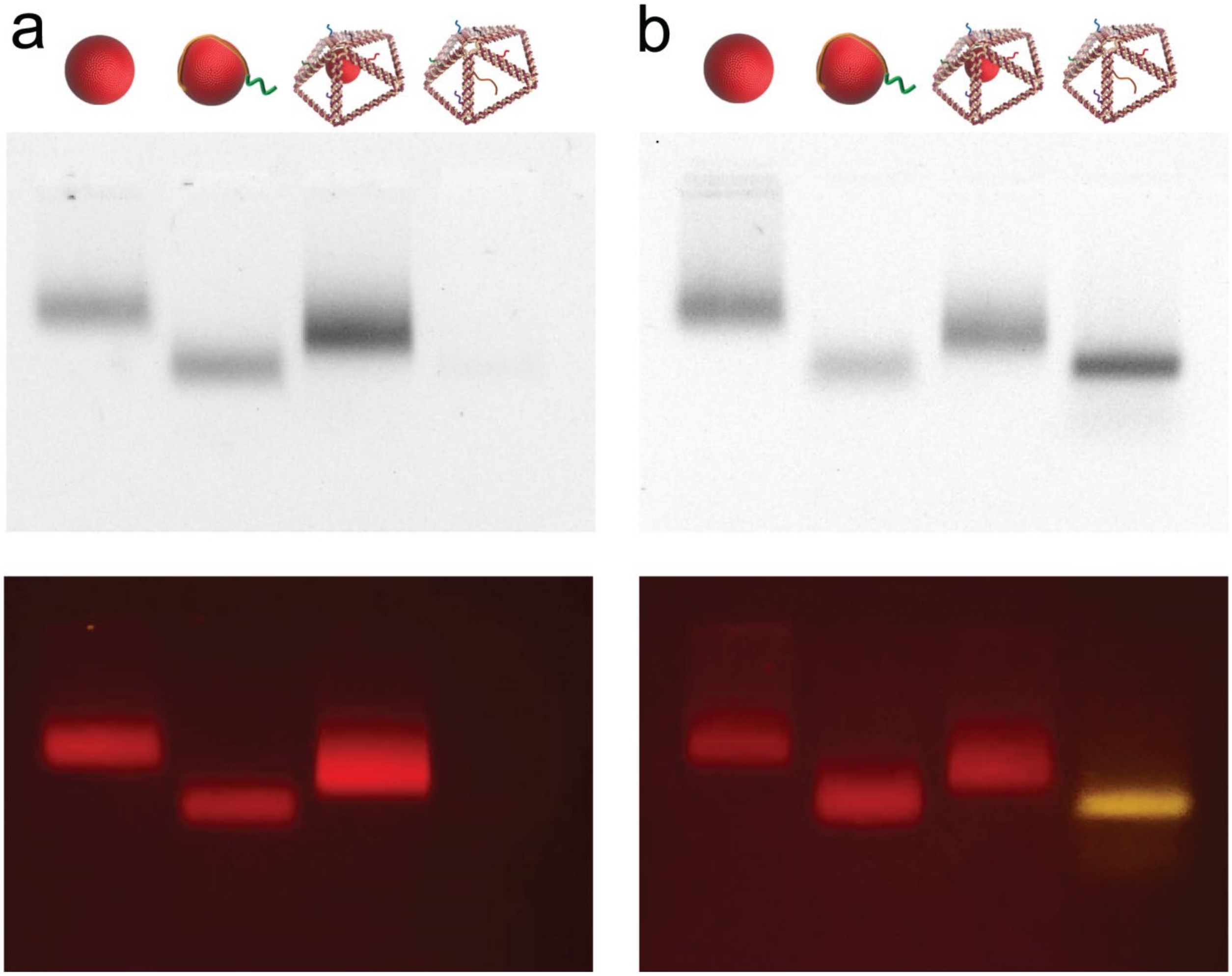

Supplementary Fig. 33. AEG (0.8 %) image of QD660 alone, QD660-50A*-staple, Pep-QD660 assemblies and Pep wireframe DNA origami objects alone (from left to right). Fluorescence images taken by gel imaging system (top) and digital camera (bottom) under blue light excitation with a) equal amount of Pep alone control and b) ten times Pep alone control. The QD660 was significantly brighter than the SYBR Safe under blue light excitation. Source data are provided as Source Data file.

D-space measuring for Tet-QD600 assemblies

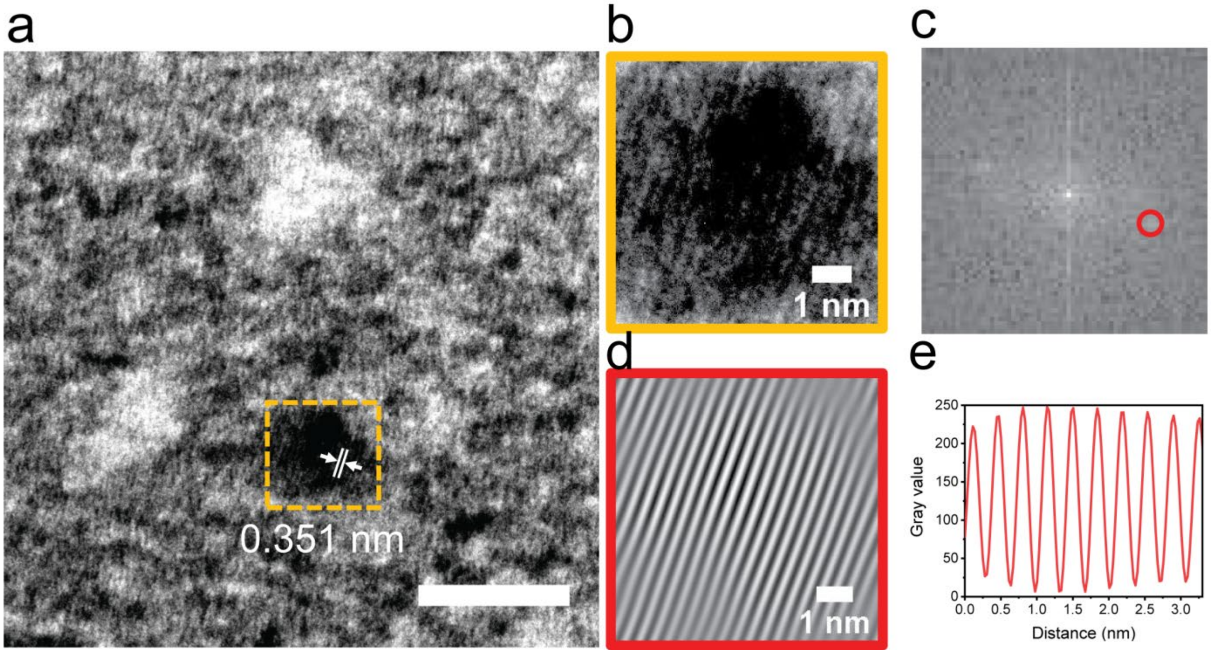

Supplementary Fig. 34. a) TEM of Tet-QD600, selected-area showed the QD600 lattice fringes (scale bar: 10 nm) and b) zoom selected-area, c) FFT of the selected region, d) inverse QD lattice FTT of red and green circle, and e) plot of inverse FFT.

D-space measuring for Pep-QD660 assemblies

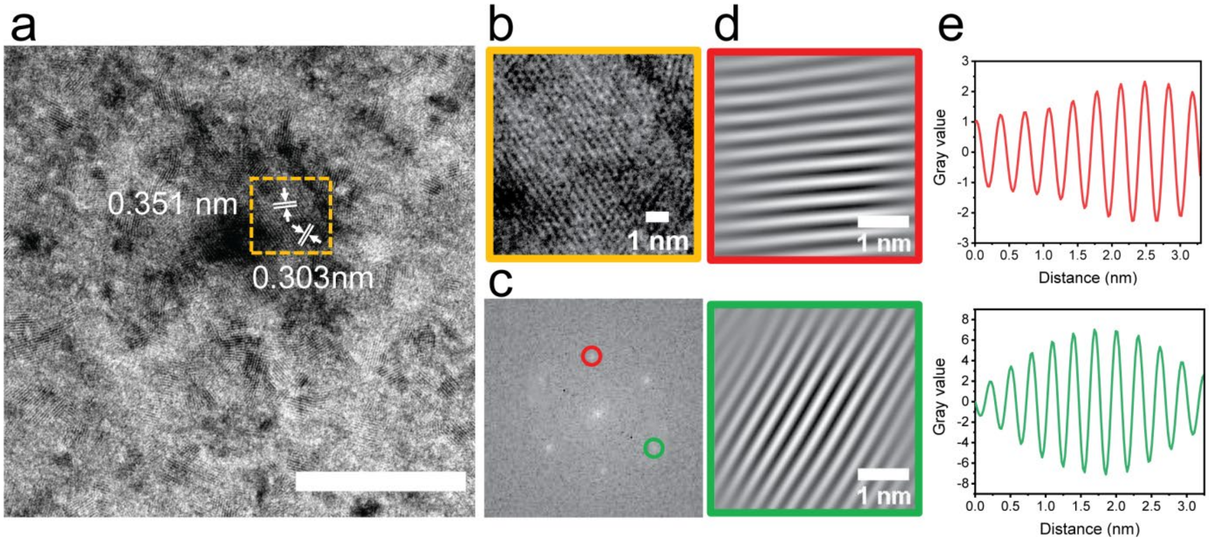

Supplementary Fig. 35. a) HRTEM of pep-QD, selected-area showed the QD660 lattice fringes (scale bar: 20 nm) and b) zoom selected-area (orange box), c) FFT of the selected region, d) inverse FTT of red and green circle, and e) plot of inverse FFT.

Incubation molar ratio effect

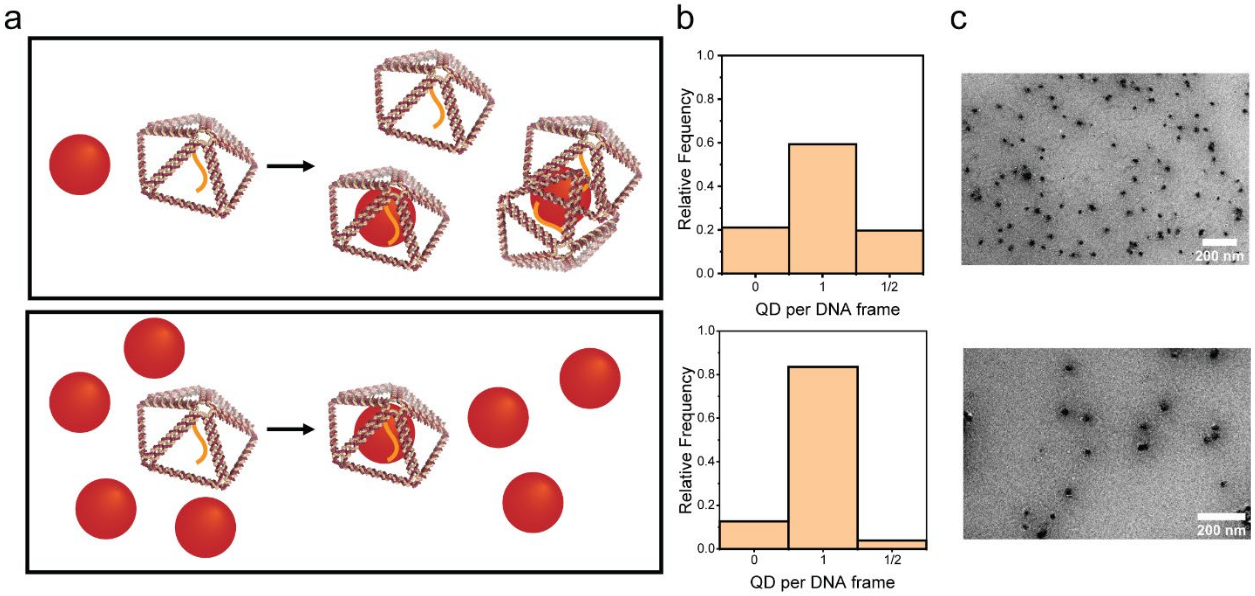

Supplementary Fig. 36. a) Schematic for preparation of Pep-QD660 assemblies using 1:1 and 4:1 initial molar ratio incubation. b) The yield of Pep-QD660 assemblies calculated from TEM images (376 NPs for 1:1 (top), 261 NPs for 4: 1 (bottom). Large clusters were not counted because they were likely due to co-localization or aggregation of clusters during TEM sample preparation[5]. c) Representative TEM images of Pep-QD660 assemblies with 1:1 (top) and 4:1(bottom) initial molar ratio. Source data are provided as Source Data file.

Schematic of DNA complex-QD-based FRET network and colloidal molecules

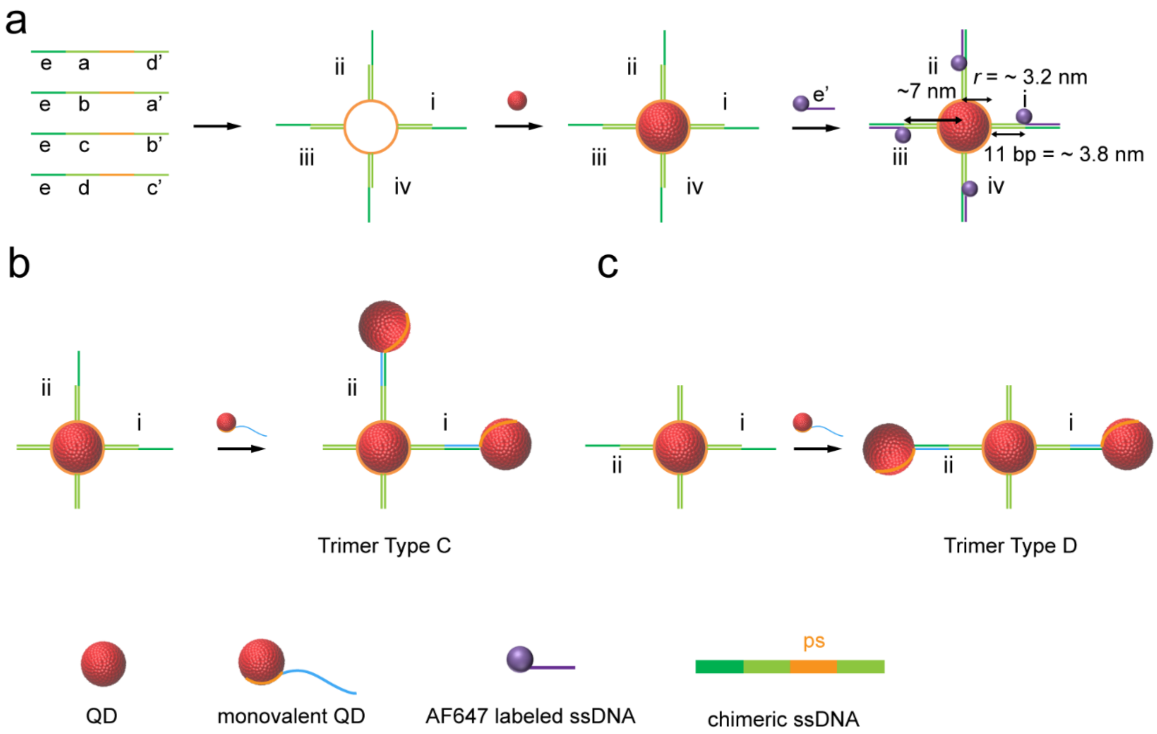

Supplementary Fig. 37. Schematic for preparation of a) DNA complex-QD-based FRET network, b) trimer Type C and c) trimer Type D. The distance between QD and dye was calculated using the duplex length (~3.8 nm) and QD radius (~3.2 nm) (DNA domains are denoted with lowercase letters, and domains x and x' are complementary to each other).

Formation of chimeric DNA complex

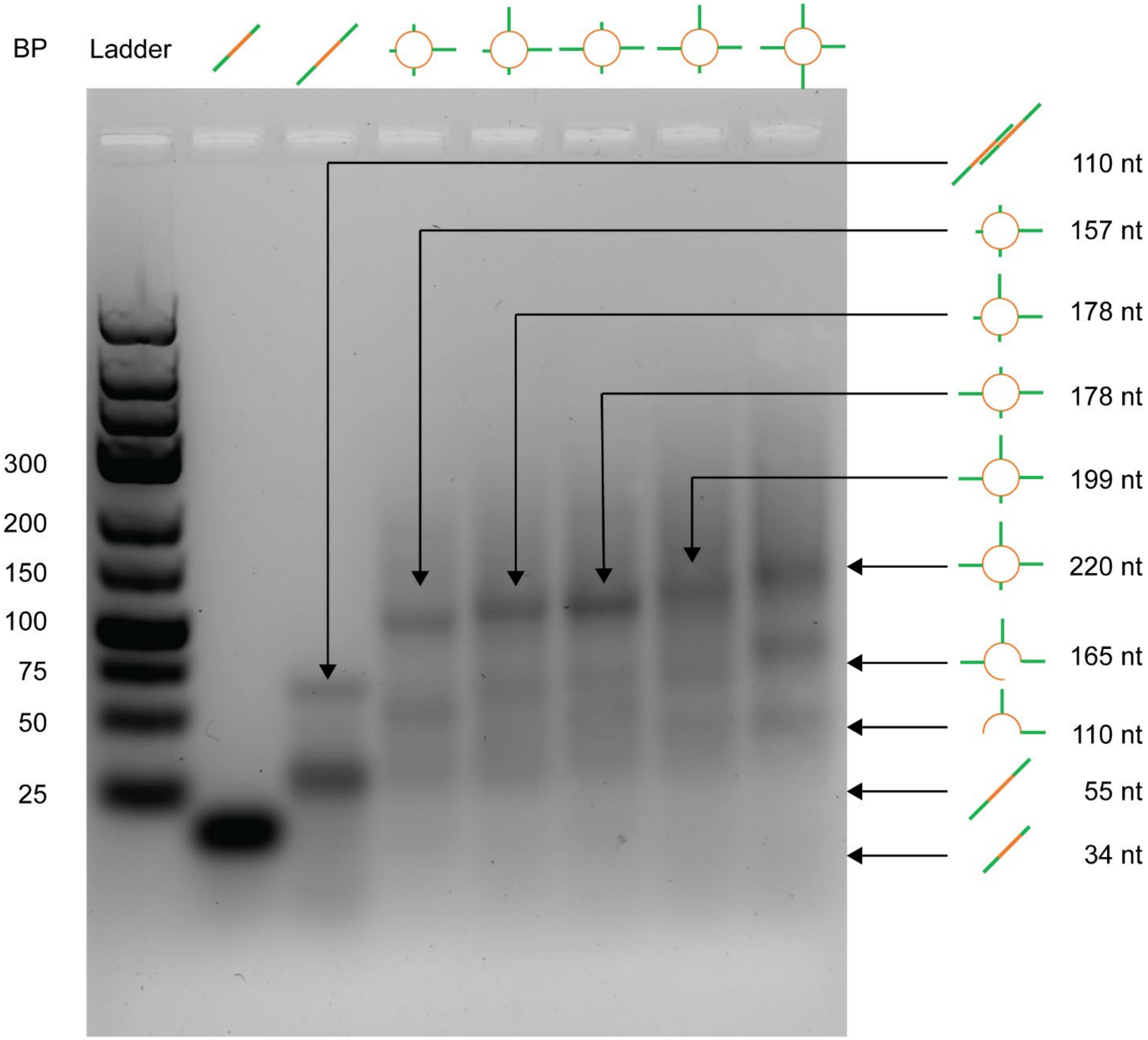

Supplementary Fig. 38. AGE (3%) image of DNA1, po1-DNA1, DNA complex with valence (I), (II) (Type C), (II) (Type D), (III), and (IV) (from left to right). All DNA sequences are listed in Supplementary Table 10.

Formation of DNA complex-QD constructs

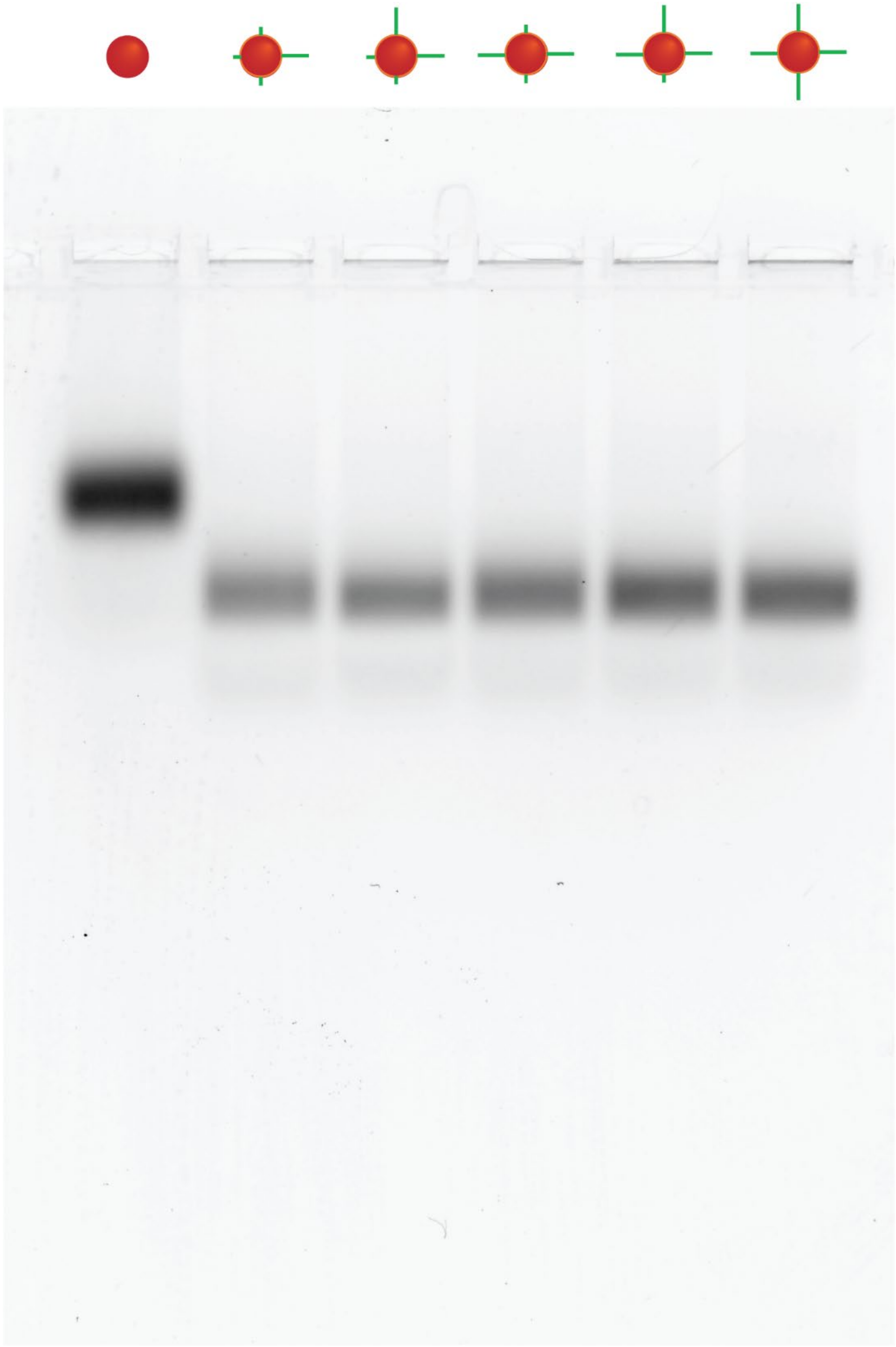

Supplementary Fig. 39. AGE (0.8%) image of QD, DNA complex-QD with valence (I), (II) (Type C), (II) (Type D), (III), and (IV) (from left to right).

Spectroscopic characterization of Tet-QD-dye and DNA complex-QD-dye-based concentric FRET network

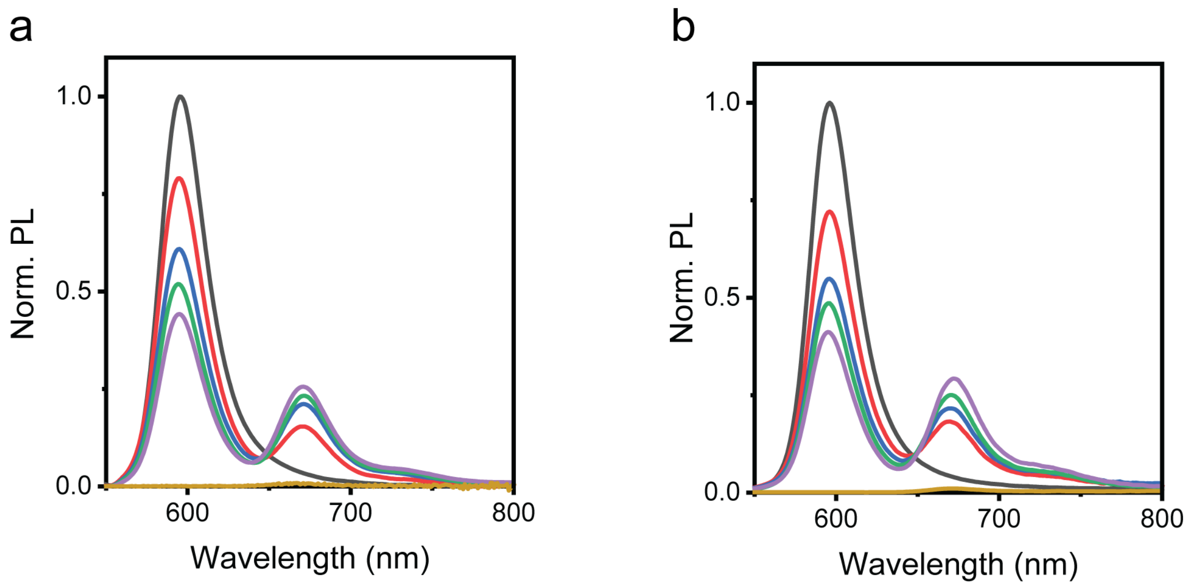

Supplementary Fig. 40. Representative PL spectra of a) Tet-QD-dye and b) DNA complex-QD-dye-based FRET network: QD donor alone (black), QD labelled with 1 (red), 2 (blue), 3 (green), and 4 (purple) AF647, and AF647 alone (orange).

Spatial addressable dye labeled Pep wireframe DNA origami objects

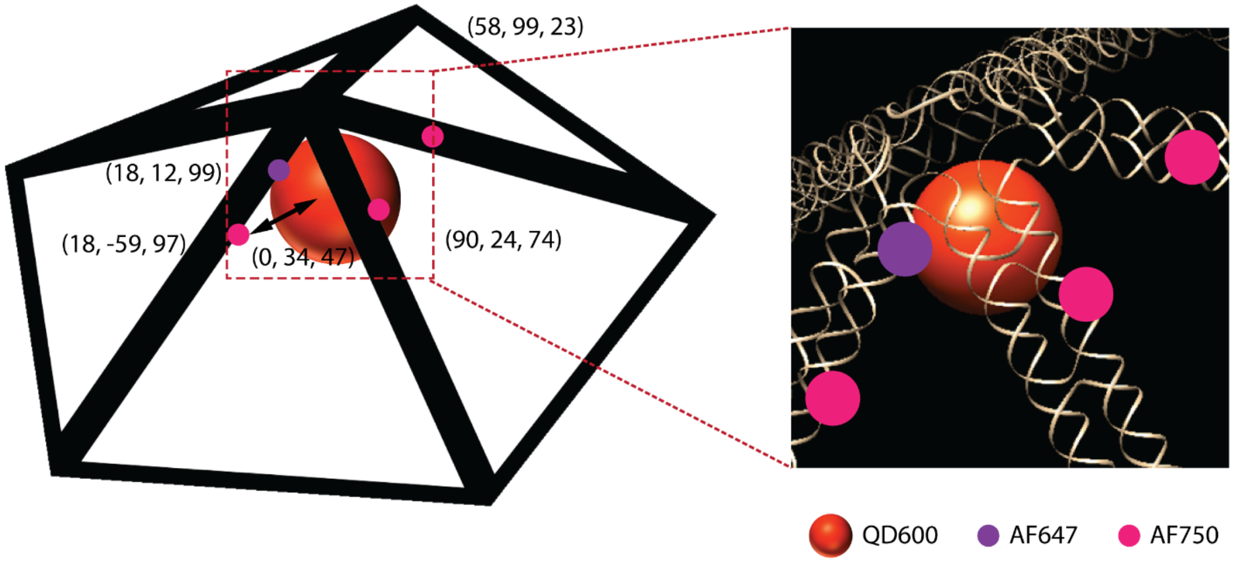

Supplementary Fig. 41. Determining the donor-relay-acceptor distance of Pep wireframe DNA origami objects based multi-step FRET networks. The coordinate of each dye was obtained using Chimera UCSF, and the coordinate of QD estimated by FRET efficiency calculated from Pep-30 nt A*-QD600-AF647, Pep-30 nt A*-QD600-AF750 (Supplementary Fig. 45).

Distance between QD and dye on Pep wireframe objects

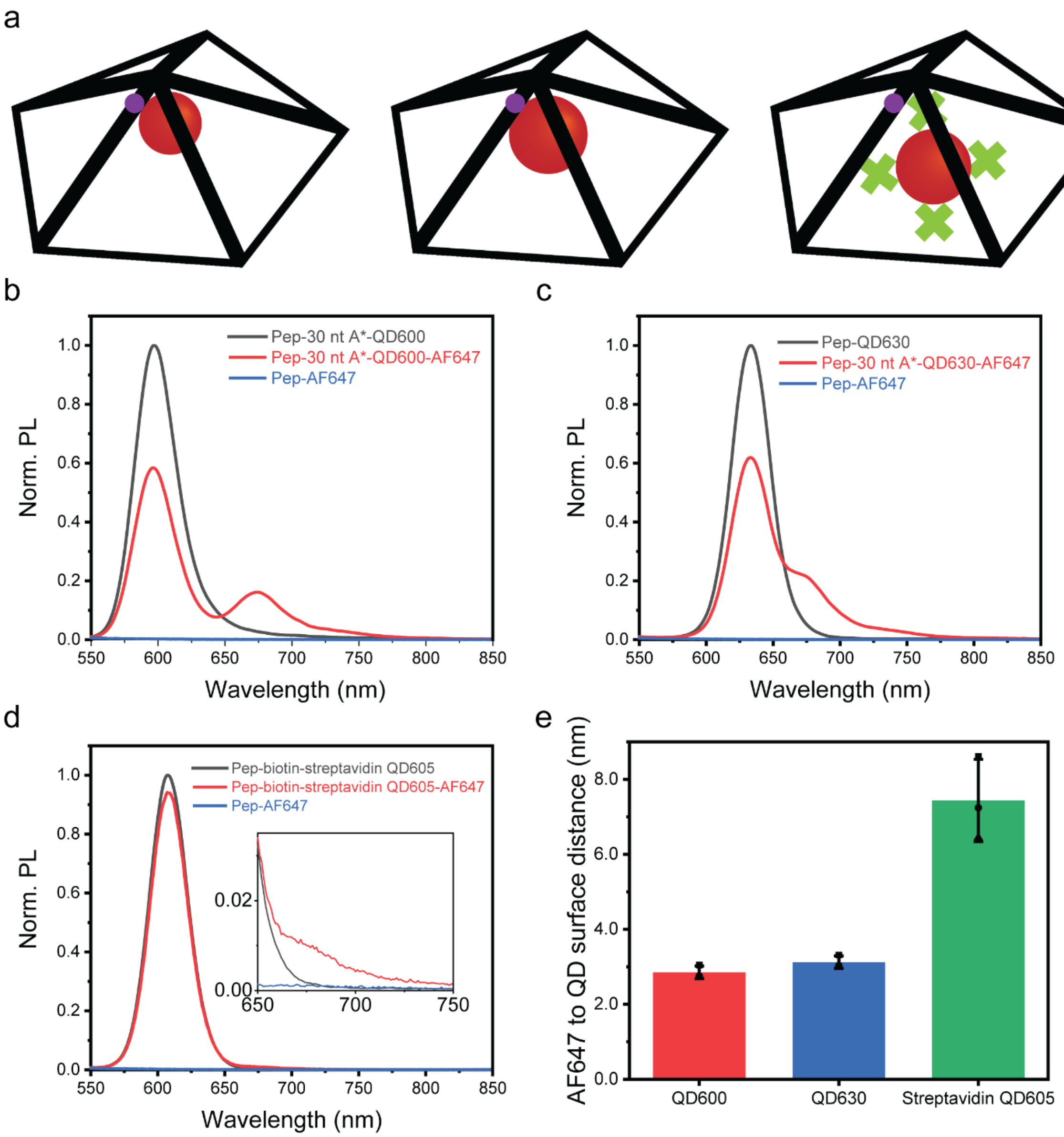

Supplementary Fig. 42. a) Schematic for Pep-30 nt A* QD600-AF647 (left), Pep-30 nt A* QD630-AF647 (middle), and Pep-biotin-streptavidin QD605-AF647 (right). Representative PL spectra of b) Pep-30 nt A* QD600-AF647, c) Pep-30 nt A* QD630-AF647, and d) Pep-biotin-streptavidin QD605-AF647. e) AF647 to QD surface distances in Pep-30 nt A* QD600-AF647 (red), Pep-30 nt A* QD630-AF647 (blue), and Pep-biotin-streptavidin QD605-AF647 (green). The distances were determined by donor-acceptor distance (Eq. (5)) and radius of QD600, QD630, and streptavidin QD605 (~3.2 nm, ~4.0 nm, and ~3.8 nm, Supplementary Figs. 2 and 16). Error bars represent standard deviation of the mean ($n = 3$ replicates per group). Source data are provided as Source Data file.

AGE images of Pep wireframe DNA origami objects-based FRET network

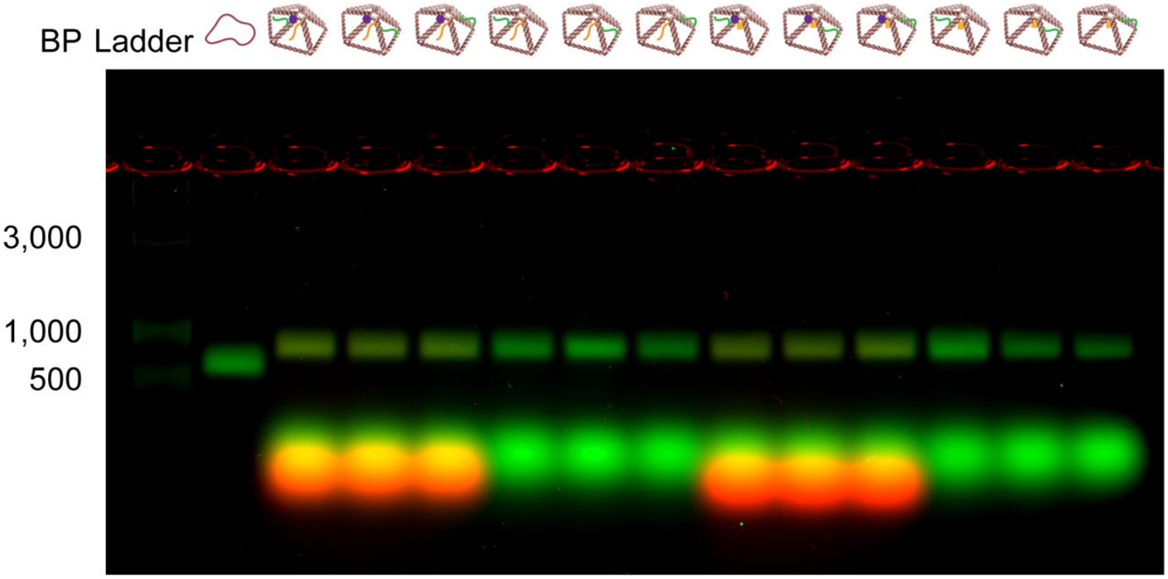

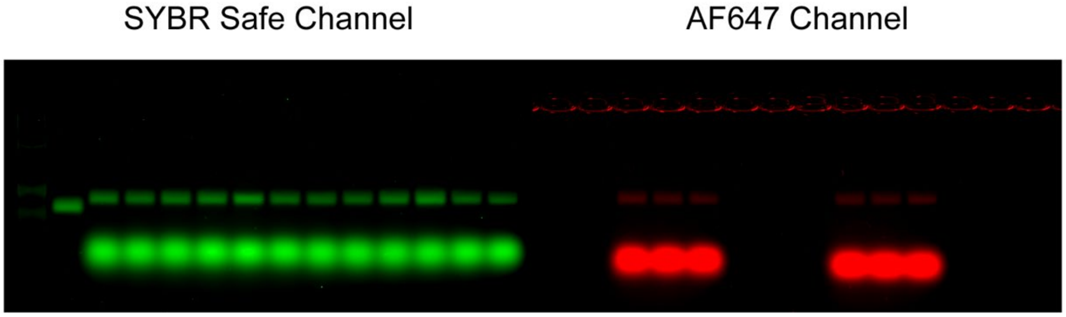

Supplementary Fig. 43. AEG (1.5%) image for Pep wireframe DNA origami objects-based FRET networks. From left to right: DNA ladder, scaffold, AF647-labeled Pep (DNA wrapping domain) with a spatial addressable overhang (close, medium, and far distance to dye), Pep (DNA wrapping) with the same spatial addressable overhang, AF647-labeled Pep (biotin domain) with a spatial addressable overhang (close, medium, and far distance to dye), and Pep (biotin domain) with the same spatial addressable overhang. Green: SYBR safe channel, Red: AF647 channel. The very bright bands visible below 500 bp are the excess of staple strands visible before purification.

Pep wireframe DNA origami objects-based AF647-AF750 FRET

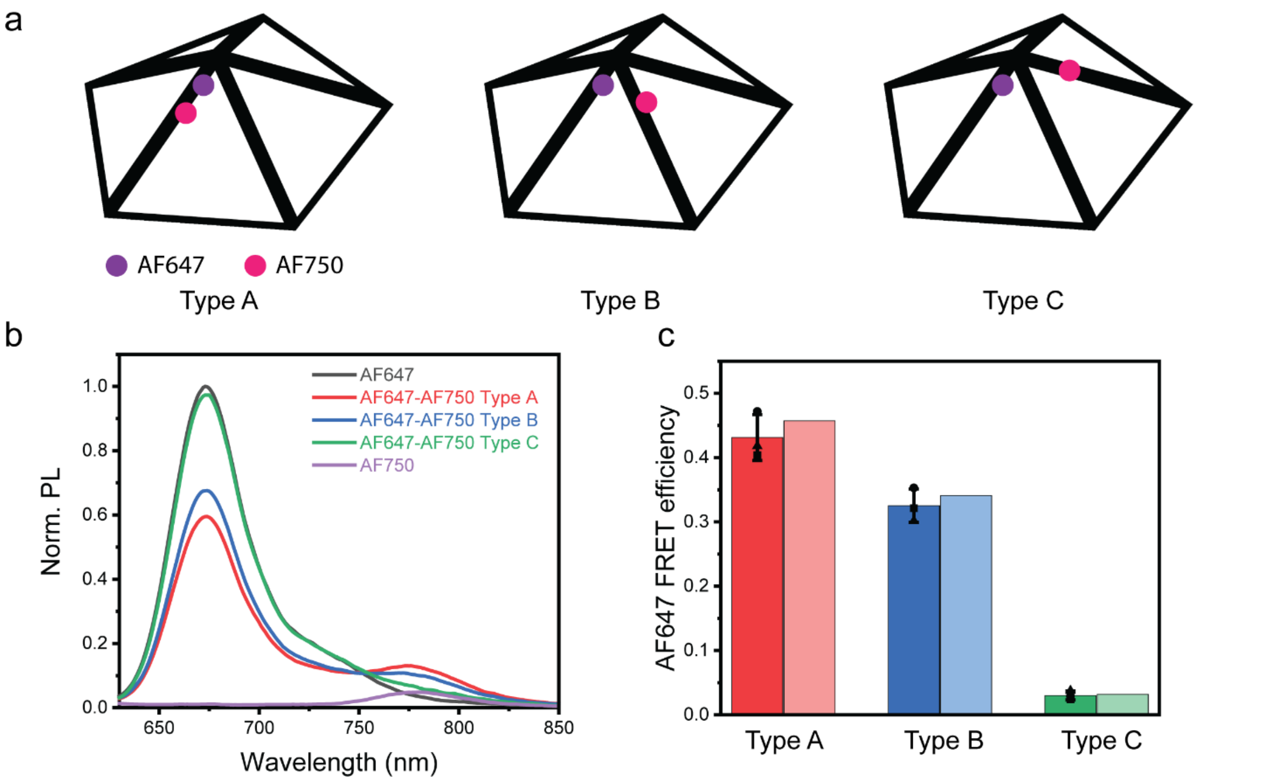

Supplementary Fig. 44. a) Schematics of three types of Pep-based AF647-AF750 FRET networks. b) Representative PL spectra of three types of Pep-based AF647-AF750 FRET networks. c) AF647 FRET efficiencies calculated from Pep-based AF647-AF750 FRET networks (darker bar), and Förster theory (lighter bar). Error bars represent standard deviation of the mean (n = 3 replicates per group). Source data are provided as Source Data file.

Spectroscopic characterization of Pep-30 nt A*-QD600-AF647-AF750 and Pep-biotin-streptavidin QD605-AF647-AF750-based multi-step FRET networks

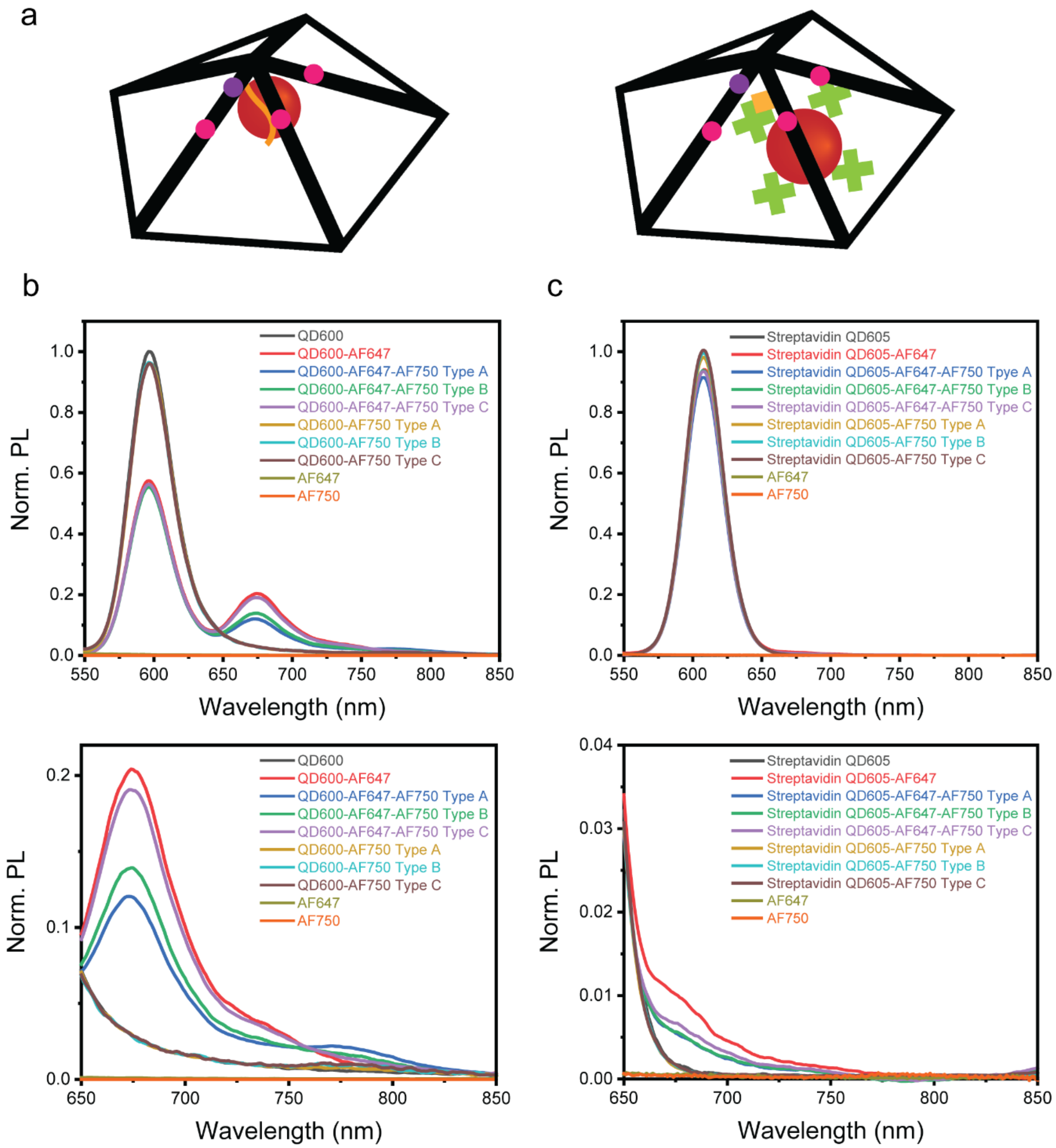

Supplementary Fig. 45. a) Schematic of Pep-30 nt A*-QD600-AF647-AF750 and Pep-biotin-streptavidin QD605-AF647-AF750-based multi-step FRET networks. Representative PL spectra (top) and zoomed area of b) Pep-30 nt A*-QD600-AF647-AF750-based multi-step FRET networks and c) Pep-biotin-streptavidin QD605-AF647-AF750-based multi-step FRET networks.

TEM images of QD trimer type-A

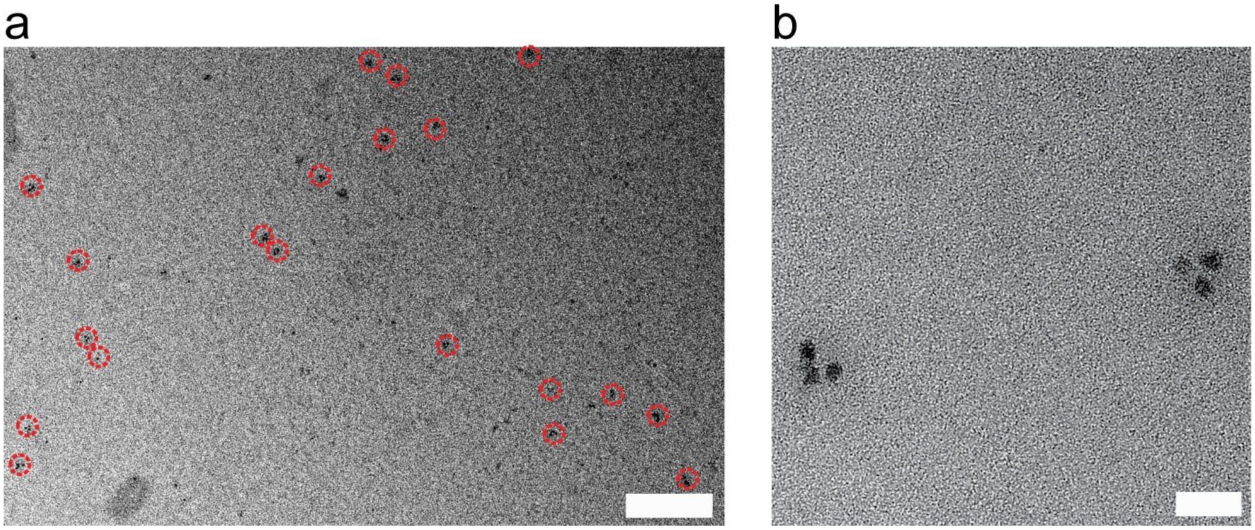

Supplementary Fig. 46. TEM images of QD trimer type-A at two magnifications. Red circles indicate correctly formed constructs. Scale bars: a) 200 nm, b) 20 nm)

TEM images of QD trimer type-B

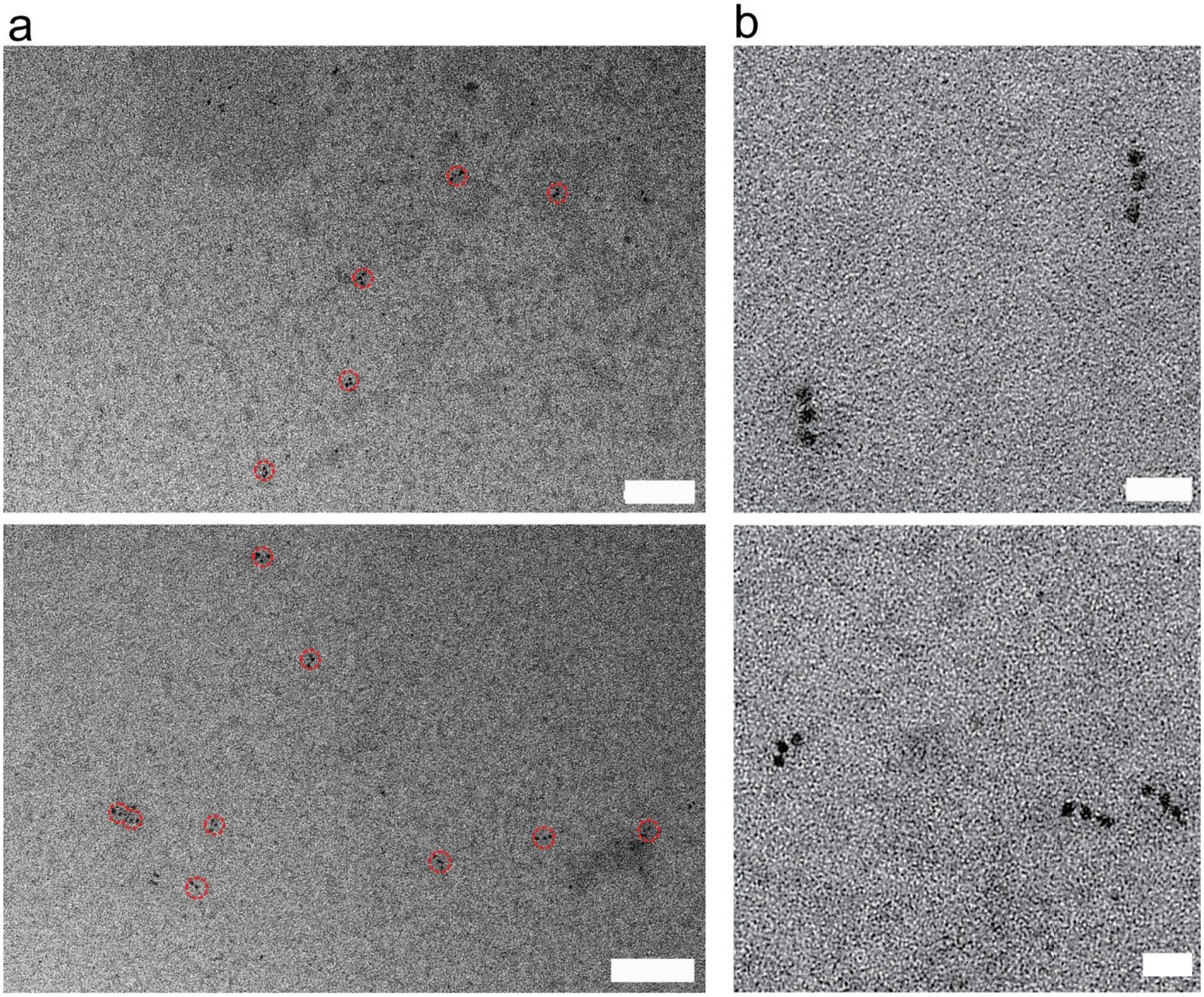

Supplementary Fig. 47. TEM images of QD trimer type-B at different magnifications. Red circles indicate correctly formed constructs. Scale bars: a) 100 nm, b) 20 nm.

TEM images of QD tetramer

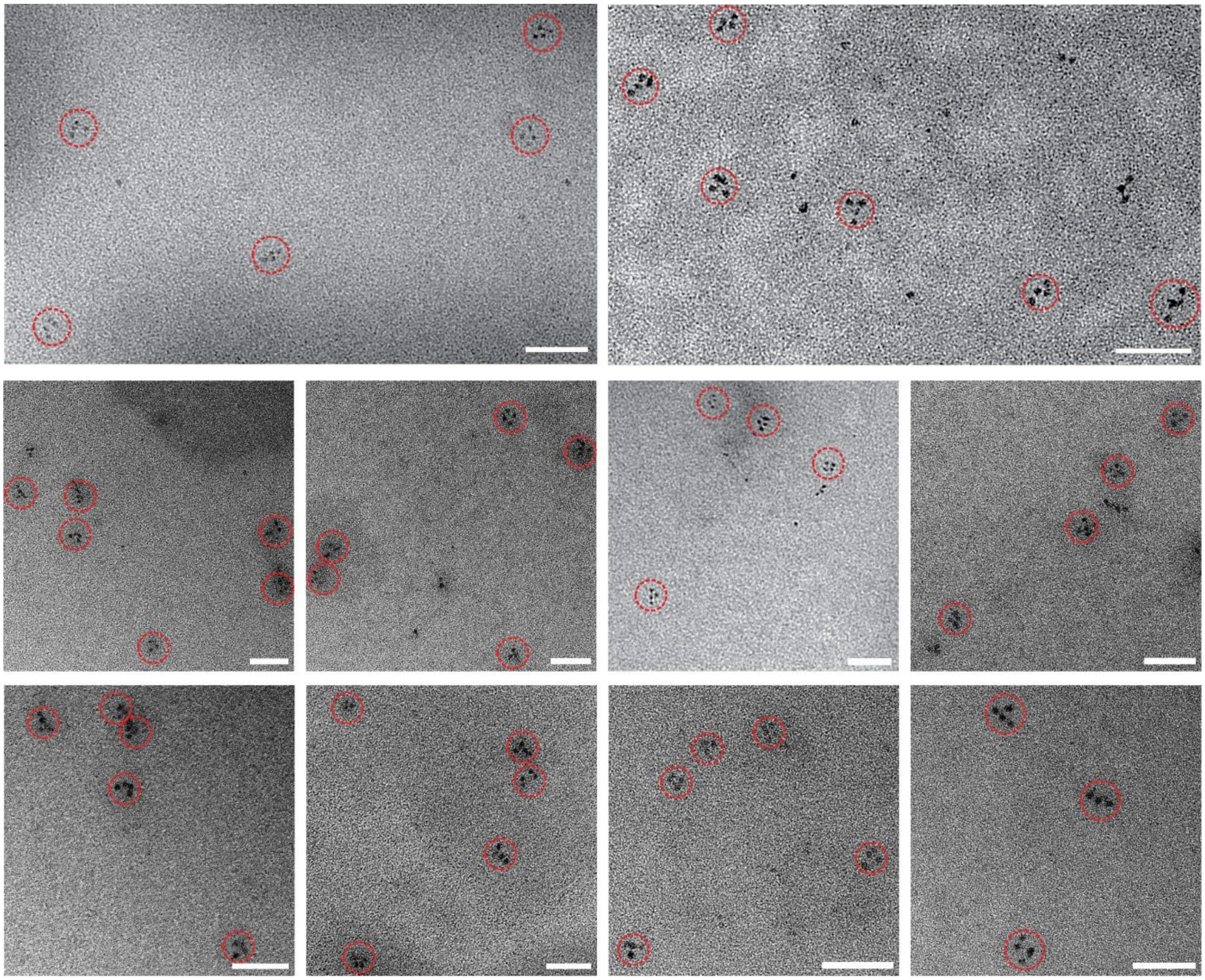

Supplementary Fig. 48. TEM images of QD tetramer at different magnifications. Red circles indicate correctly formed constructs. Scale bar: 50 nm.

TEM images of QD pentamer

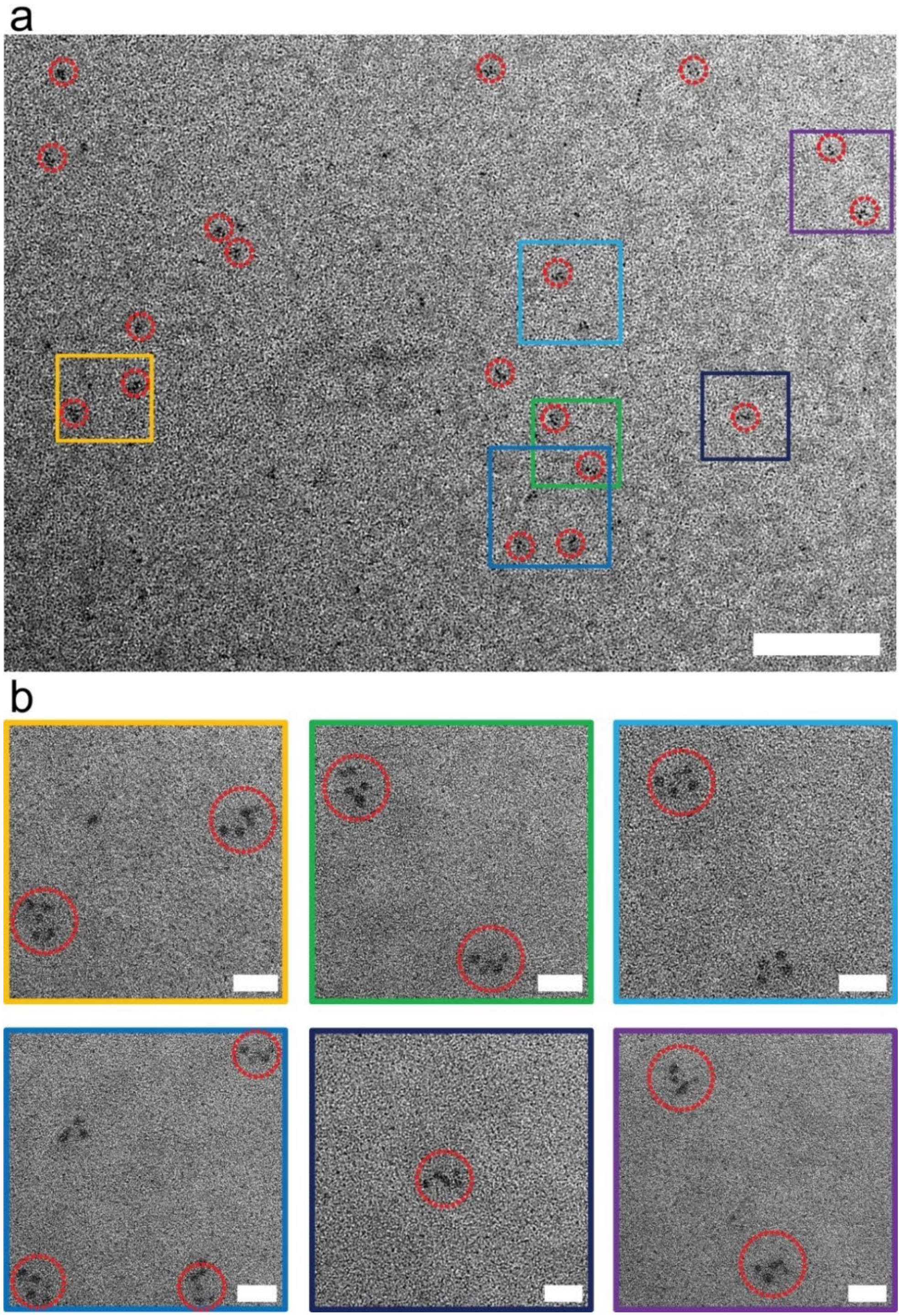

Supplementary Fig. 49. a) Wide field and b) randomly selected zoomed areas (colored borders) from TEM images of QD pentamer. Red circles indicate correctly formed constructs. Scale bars: a) 200 nm, b) 20 nm.

TEM images of QD hexamer

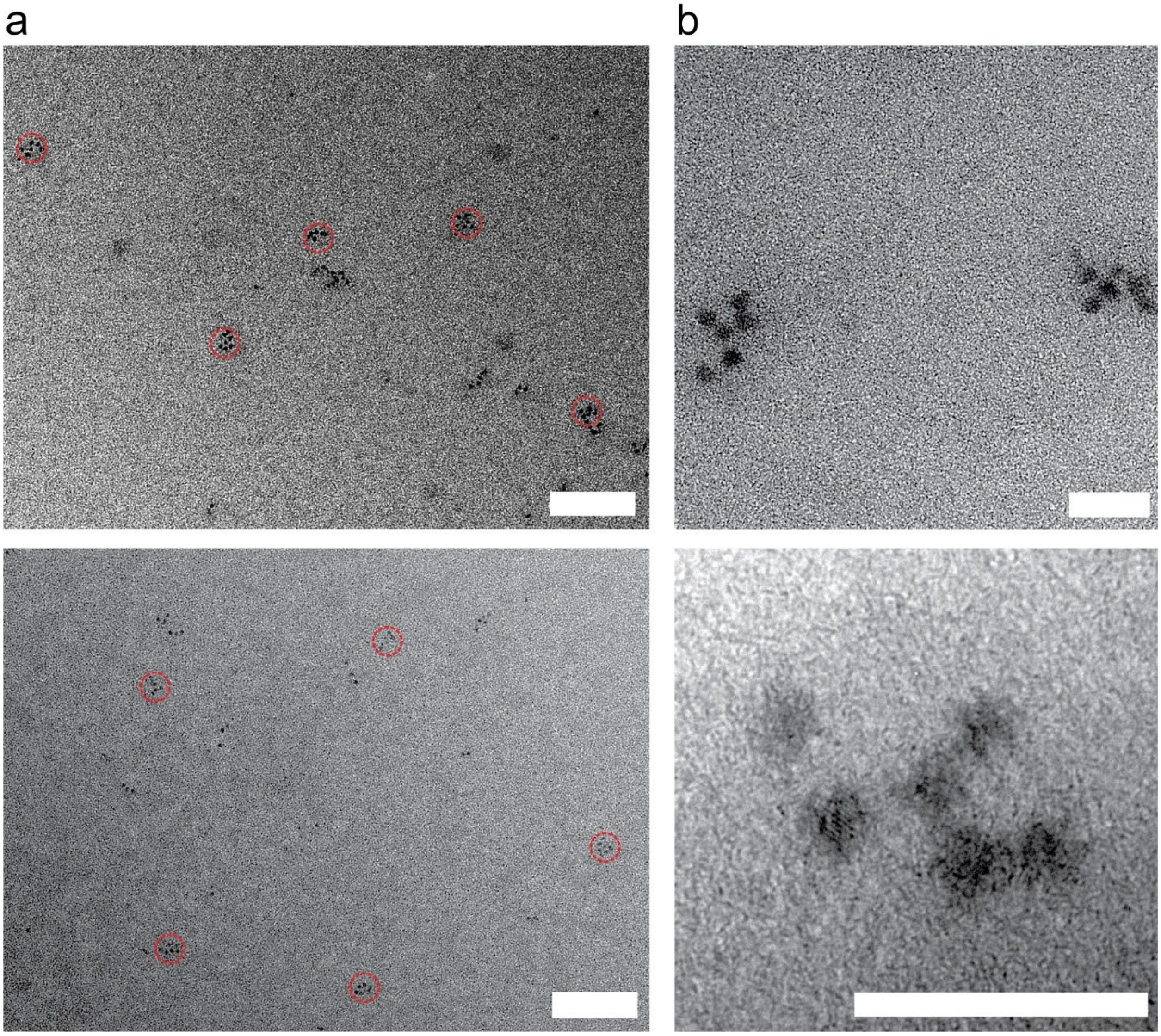

Supplementary Fig. 50. TEM images of QD hexamer at different magnifications. Red circles indicate correctly formed constructs. Scale bars: a) 100 nm, b) 20 nm.

Statistical distributions of different QD-based colloidal molecules

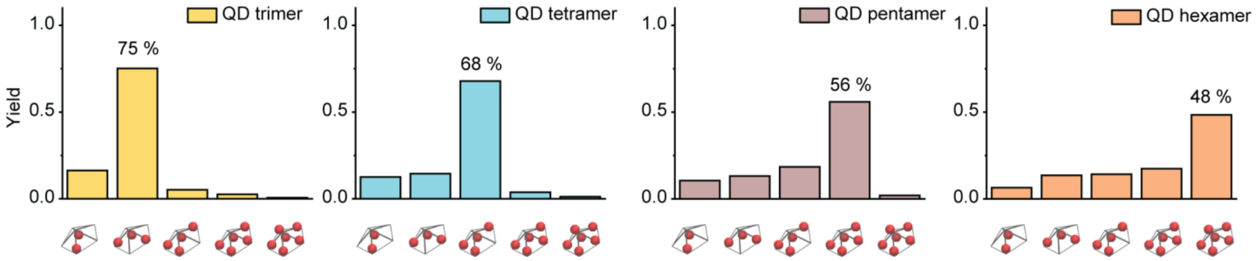

Supplementary Fig. 51. Statistical distributions of different QD-based colloidal molecules observed in TEM experiments. Large clusters were not counted because they were likely due to co-localization or aggregation of clusters during TEM sample preparation[5]. Each statistical distribution was generated using >150 individual QD-based colloidal molecules. Source data are provided as Source Data file.

MD simulation model with atom labels and partial charges

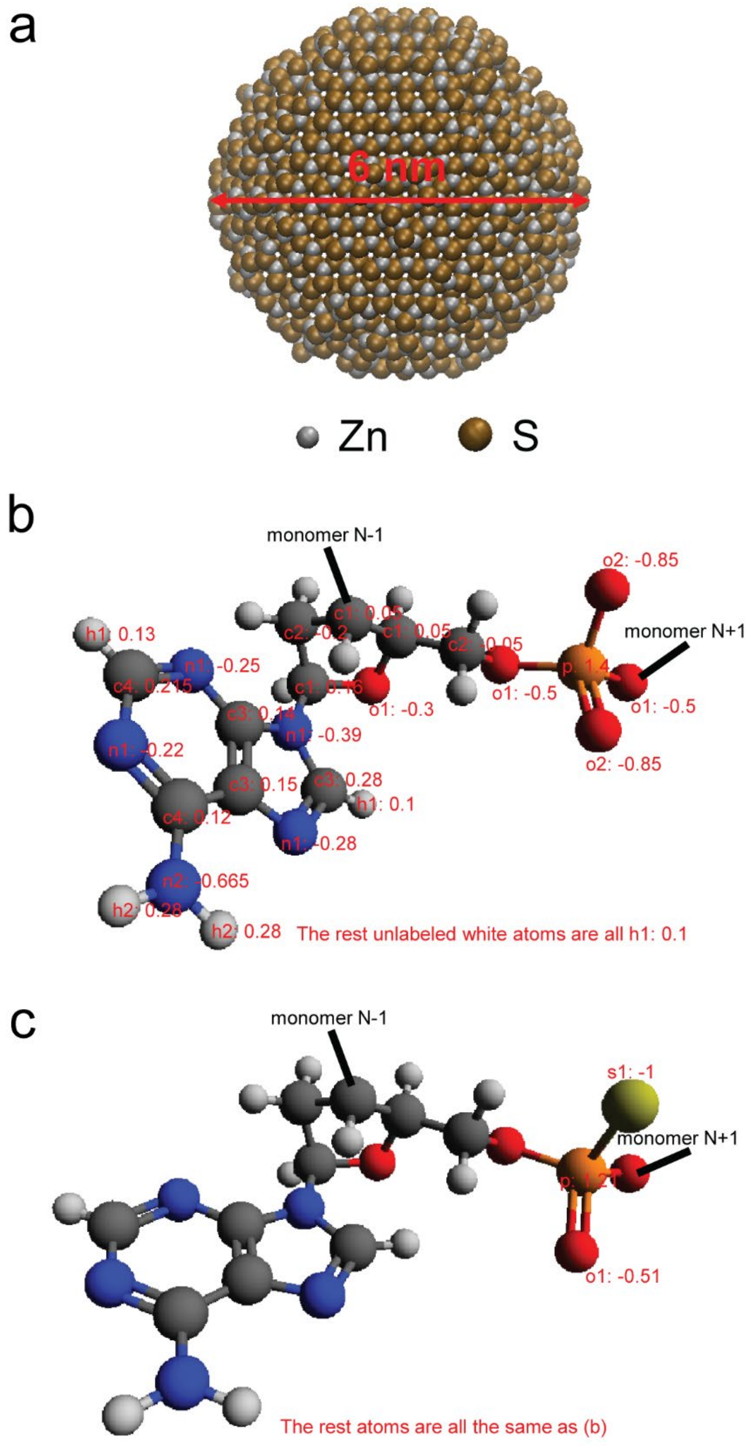

Supplementary Fig. 52. The MD simulation models for a) 6 nm diameter ZnS NP, b) deoxyadenosine monophosphate, and c) deoxyadenosine phosphorothioate. The atom labels and partial charges are signed on each atom in b) and c).

Supplementary Tables

Supplementary Table 1. Comparison of various DNA-based strategies to engineer valences on QDs.

Reference [citation number in main MS]	Method	Controllable valence number	Controllable valence position
Nat. Nanotechnol. 2011[16][6]	one pot synthesis (ps+po backbone)	1-5	no
Nat. Methods 2013[17][7]	ssDNA wrapping (ps+po backbone)	1	N/A
Angew. Chem. 2017[18][2]	ssDNA wrapping (programmable DNA complexes)	1-4	no
This work	ssDNA wrapping and folding (QD-wireframe DNA origami objects assemblies)	1 to >5 (limited only by the number of overhangs on the wireframe DNA origami)	yes (valence-geocoded)

Supplementary Table 2. Photophysical Properties of the FRET Compounds within the DNA wrapping experiment[a]

**Fluorophore**	**ε (M^{-1} cm^{-1}) [λ]**	**Φ**
AF647	2.39×10^5 [650 nm]	0.33
AF750	2.40×10^5 [751 nm]	N/A
QD600	2.09×10^5 [581 nm]	0.20 ± 0.05
QD630	4.79×10^5 [614 nm]	0.20 ± 0.05
QD660	7.48×10^5 [630 nm]	0.15 ± 0.05
Streptavidin QD605	4.40×10^6 [350 nm]	0.60 ± 0.10
**FRET pair (D → A)**	**J (M^{-1}. Cm^{-1}. Nm^4)**	**R_0 (nm)**
QD600 → AF647	1.0×10^{16}	5.7 ± 0.2
QD630 → AF647	2.1×10^{16}	6.4 ± 0.2
QD660 → AF750	8.8×10^{16}	5.3 ± 0.3
Streptavidin QD605-AF647	1.1×10^{16}	7.0 ± 0.2
AF647 → AF750	1.9×10^{16}	6.9 ± 0.2
QD600 → AF750	3.9×10^{15}	4.9 ± 0.2
Streptavidin QD605-AF750	2.4×10^{15}	5.4 ± 0.2

a) ε and Φ values of AF647, AF750, and Streptavidin QD605 were provided by the commercial vendors. E and Φ values of QD were calculated using empirical formula[8] and relative quantum yield determination method[9], respectively. J was calculated using Eq. (1). R_0 was calculated using Eq. (2).

Supplementary Table 3. P-values, t statistic, and degree of freedom for Fig. 5d.

Tet-QD	DNA-complexes-QD	*P*-values	t statistic	DF
1AF647	1AF647	0.02853	-3.35142	4
2AF647	2AF647	0.01850	-3.83735	4
3AF647	3AF647	0.07680	-2.37028	4
4AF647	4AF647	0.10978	-2.04930	4

Supplementary Table 4. DNA sequences for QD wrapping and QD-dye FRET pairs.

Orange text denotes ps tract for DNA wrapping while green text indicates the po tract for the DNA hybridization domain, 1 nt of A or T was added to the 3' end since it cannot be phosphorothioated.

Name	Length (nt)	Sequences (5'-3')
po1-(TT)-5A*	29	TTGTGACAGCTGGATCGTTACTTA*A*A*A*A*A
po1-(TT)-10A*	34	TTGTGACAGCTGGATCGTTACTTA*A*A*A*A*A*A*A*A*A*A
po1-(TT)-30A*	54	TTGTGACAGCTGGATCGTTACTTA*A*A*A*A*A*A*A*A*A*A*A*A*A*A*A*A*A*A*A*A*A*A*A*A*A*A*A*A*A*A
po(c1)-TT-30A*	54	GTAACGATCCAGCTGTCACAATTA*A*A*A*A*A*A*A*A*A*A*A*A*A*A*A*A*A*A*A*A*A*A*A*A*A*A*A*A*A*A
po1-(TT)-50A*	74	TTGTGACAGCTGGATCGTTACTTA*A*A*A*A*A*A*A*A*A*A*A*A*A*A*A*A*A*A*A*A*A*A*A*A*A*A*A*A*A*A*A*A*A*A*A*A*A*A*A*A*A*A*A*A*A*A*A*A*A*T
po(c1)-30A*	54	GTAACGATCCAGCTGTCACAATTA*A*A*A*A*A*A*A*A*A*A*A*A*A*A*A*A*A*A*A*A*A*A*A*A*A*A*A*A*A*A
po1-(TTTTT)-SH	26	TTGTGACAGCTGGATCGTTACTTTTT/3Thio/
po1-(TT)-biotin	23	TTGTGACAGCTGGATCGTTAC TT/3Bio/
AF647-po(c1)	21	/5Alex647N/GTAACGATCCAGCTGTCACAA
AF750-po(c1)	21	/5Alex750N/GTAACGATCCAGCTGTCACAA

Supplementary Table 5. DNA sequences for ps tract length effect.

(Orange text indicates ps tract for the DNA wrapping domain)

Name	Length (nt)	Sequences (5'-3')
30A-random (po)	51	AAAAAAAAAAAAAAAAAAAAAAAAAAAAAATTGTGACAGCTGGATCGTTAC
5A*-random (po)	51	A*A*A*A*A*TTCGACGACACGATAAGCCATAGCCTTGTGACAGCTGGATCGTTAC
10A*-random (po)	51	A*A*A*A*A*A*A*A*A*A*TTACACGATAAGCCATAGCCTTGTGACAGCTGGATCGTTAC
20A*-random (po)	51	A*A*A*A*A*A*A*A*A*A*A*A*A*A*A*A*A*A*A*A*TTCCATAGCCTTGTGACAGCTGGATCGTTAC
30A*-random (po)	51	A*A*A*A*A*A*A*A*A*A*A*A*A*A*A*A*A*A*A*A*A*A*A*A*A*A*A*A*A*A*TTGTGACAGCTGGATCGTTAC
40A*-random (po)	51	A*A*A*A*A*A*A*A*A*A*A*A*A*A*A*A*A*A*A*A*A*A*A*A*A*A*A*A*A*A*A*A*A*A*A*A*A*A*A*A*TTG ATCGTTAC
SH-random (po)	51	/5Thio/AAAAAAAAAAAAAAAAAAAAAAAAAAAAAATTGTGACAGCTGGATCGTTAC

Supplementary Table 6. Scaffold sequences for wireframe DNA origami objects.

(phPB84 for tetrahedron and pF1A for pentagonal pyramid)

Scaffold	Length (nt)	Sequences (5’-3’)
phPB84	2520	GAGCGCAACGCAATTAATGTGCGCCCTGTAGCGGCGCATTAAGCGCGGCGGGTGTGGTGGTTACGCGCAGC<br>GTGACCGCTACACTTGCCAGCGCCCTAGCGCCCGCTCCTTTCGCTTTCTTCCCTTCCTTTCTCGCCACGTTCGC<br>CGGCTTTCCCCGTCAAGCTCTAAATCGGGGGCTCCCTTTAGGGTTCCGATTTAGTGCTTTACGGCACCTCGAC<br>CCCAAAAAACTTGATTAGGGTGATGGTTCACGTAGTGGGCCATCGCCCTGATAGACGGTTTTTCGCCCTTTG<br>ACGTTGGAGTCCACGTTCTTTAATAGTGGACTCTTGTTCCAAACTGGAACAACACTCAACCCTATCTCGGTCT<br>ATTCTTTTGATTTATAAGGGATTTTGCCGATTTCGGCCTATTGGTTAAAAAATGAGCTGATTTAACAAAAATT<br>TAACGCGAATTACAACCGGGGTACATATGATTGGGGTCTGACGCTCAGTGGAACGAAAACTCACGTTAAGG<br>GATTTTGGTCATGAGATTATCAAAAAGGATCTTCACCTAGATCCTTTTAAATTAAAAATGAAGTTTTAAATCA<br>ATCTAAAGTATATATGAGTAAACTTGGTCTGACAGTTACCAATGCTTAATCAGTGAGGCACCTATCTCAGCG<br>ATCTGTCTATTTCGTTCATCCATAGTTGCCTGACTCCCCGTCGTGTAGATAACTACGATACGGGAGGGCTTAC<br>CATCTGGCCCCAGTGCTGCAATGATACCGCGAGACCCACGCTCACCGGCTCCAGATTTATCAGCAATAAACC<br>AGCCAGCCGGAAGGGCCGAGCGCATAAGTGGTCCTGCAACTTTATCCGCCTCCATCCAGTCTATTAATTGTT<br>GCCGGGAAGCTAGAGTAAGTAGTTCGCCAGTTAATAGTTTGCGCAACGTTGTTGCCATTGCTACAGGCATCG<br>TGGTGTCACGCTCGTCGTTTGGTATGGCTTCATTCAGCTCCGGTTCCCAACGATCAAGGCGAGTTACATGATC<br>CCCCATGTTGTGCAAAAAAGCGGTTAGCTCCTTCGGTCCTCCGATCGTTGTCAGAAGTAAGTTGGCCGCAGT<br>GTTATCACTCATGGTTATGGCAGCACTGCATAATTCTCTTACTGTCATGCCATCCGTAAGATGCTTTTCTGTG<br>ACTGGTGAGTACTCAACCAAGTCATTCTGAGAATAGTGTATGCGGCGACCGAGTTGCTCTTGCCCGGCGTCA<br>ATACGGGATAATACCGCGCCACATAGCAGAACTTTAAAAGTGCTCATCATTGGAAAACGTTCTTCGGGGCGA<br>AAACTCTCAAGGATCTTACCGCTGTTGAGATCCAGTTCGATGTAACCCACTCGTGCACCCAACTGATCTTCAG<br>CATCTTTTACTTTCACCAGCGTTTCTGGGTGAGCAAAAACAGGAAGGCAAAATGCCGCAAAAAAGGGAATA<br>AGGGCGACACGGAAATGTTGAATACTCATACTCTTCCTTTTTCAATATTATTGAAGCATTTATCAGGGTTATT<br>GTCTCATGAGCGGATACATATTTGAATGTATTTAGAAAAATAAACAAATAGGGGTTCCGCGCACATTTCCCC<br>GAAAAGTGCCACCTGACGTCTAAGAAACCATTATTATCATGACATTAACCTATAAAAATAGGCGTATCACGA<br>GGCCCTTTCGTCGAATTCGTCGTCGTCCCCTCAAACTCTTGGGTGGAGAGGCTATTCGTTTAAGGTCACATCG<br>CATGTAATTTACTTATTCTCTGTTGTTGAGCCACCCGGGCGCCAGATTTTGTTTAAAGCTTTGTCTCTTAGTTT<br>GTATAGACAGATTCAGAGTGCAAGGTTTCGTTCGCTCGTACCTGGTTTTCCCTGGTTCTTCACAGATAGGATT<br>TGACTTTCTACAACACTTATGCGGCTTCCTACCCGTTTGAAGGCCGATACAGGTGCTGCGCAAAATGCGGGC<br>GAACATAGAGTATCAAAACAACGCCTTCTAATCTAGGAATATAGGGAAGATACGTATTTGCTACCATGCTTT<br>CTTGGGTCATTAACGACCAACCTCTTTTCTTTTAAAGTAGGATTGCACAATGAATGAATACACGTGGTCCGAT<br>AACTGACCAAGTAACATGGTTATCACTaGATGTCCGCCAGACGTGTGCAAACCAACCCGGGAGTTACGTCAC<br>TAATCCTTCGCTACGTCGTGAAGATATTTACTTGTGAATATCGAGGGTAATAAGATAATAGACTGTGACTAG<br>TATTGCCAGACTGTCGCTACCTGCAACACATAACTATCCTGAGGTTACTGCATAGTACTGATTACACCCGAGT<br>CAAAATTTCTAACTTCTAACATGTACCTAGTAACCAGCTCAATAATTATGTCAGAATATAGCTCTGGGAACC<br>CTCGGACAATTATGATACACGGTATTAATATCTTGCTTGCGTTAGCCACTTCTCATCTTTGGATACCGATTCT<br>ATTTTGCATAGCAGTTCCTTTTACACATATAAGAATTTCGCCATAGGTATGCTGCAG
pF1A	1676	GAGCGCAACGCAATTAATGTGCGCCCTGTAGCGGCGCATTAAGCGCGGCGGGTGTGGTGGTTACGCGCAGC<br>GTGACCGCTACACTTGCCAGCGCCCTAGCGCCCGCTCCTTTCGCTTTCTTCCCTTCCTTTCTCGCCACGTTCGC<br>CGGCTTTCCCCGTCAAGCTCTAAATCGGGGGCTCCCTTTAGGGTTCCGATTTAGTGCTTTACGGCACCTCGAC<br>CCCAAAAAACTTGATTAGGGTGATGGTTCACGTAGTGGGCCATCGCCCTGATAGACGGTTTTTCGCCCTTTG<br>ACGTTGGAGTCCACGTTCTTTAATAGTGGACTCTTGTTCCAAACTGGAACAACACTCAACCCTATCTCGGTCT<br>ATTCTTTTGATTTATAAGGGATTTTGCCGATTTCGGCCTATTGGTTAAAAAATGAGCTGATTTAACAAAAATT<br>TAACGCGAATTACAACCGGGGTACATATGATTGGGGTCTGACGCTCAGTGGAACGAAAACTCACGTTAAGG<br>GATTTTGGTCATGAGATTATCAAAAAGGATCTTCACCTAGATCCTTTTAAATTAAAAATGAAGTTTTAAATCA<br>ATCTAAAGTATATATGAGTAAACTTGGTCTGACAGTTACCAATGCTTAATCAGTGAGGCACCTATCTCAGCG<br>ATCTGTCTATTTCGTTCATCCATAGTTGCCTGACTCCCCGTCGTGTAGATAACTACGATACGGGAGGGCTTAC<br>CATCTGGCCCCAGTGCTGCAATGATACCGCGAGACCCACGCTCACCGGCTCCAGATTTATCAGCAATAAACC<br>AGCCAGCCGGAAGGGCCGAGCGCAGAAGTGGTCCTGCAACTTTATCCGCCTCCATCCAGTCTATTAATTGTT<br>GCCGGGAAGCTAGAGTAAGTAGTTCGCCAGTTAATAGTTTGCGCAACGTTGTTGCCATTGCTACAGGCATCG<br>TGGTGTCACGCTCGTCGTTTGGTATGGCTTCATTCAGCTCCGGTTCCCAACGATCAAGGCGAGTTACATGATC<br>CCCCATGTTGTGCAAAAAAGCGGTTAGCTCCTTCGGTCCTCCGATCGTTGTCAGAAGTAAGTTGGCCGCAGT<br>GTTATCACTCATGGTTATGGCAGCACTGCATAATTCTCTTACTGTCATGCCATCCGTAAGATGCTTTTCTGTG<br>ACTGGTGAGTACTCAACCAAGTCATTCTGAGAATAGTGTATGCGGCGACCGAGTTGCTCTTGCCCGGCGTCA<br>ATACGGGATAATACCGCGCCACATAGCAGAACTTTAAAAGTGCTCATCATTGGAAAACGTTCTTCGGGGCGA<br>AAACTCTCAAGGATCTTACCGCTGTTGAGATCCAGTTCGATGTAACCCACTCGTGCACCCAACTGATCTTCAG<br>CATCTTTTACTTTCACCAGCGTTTCTGGGTGAGCAAAAACAGGAAGGCAAAATGCCGCAAAAAAGGGAATA<br>AGGGCGACACGGAAATGTTGAATACTCATACTCTTCCTTTTTCAATATTATTGAAGCATTTATCAGGGTTATT<br>GTCTCATGAGCGGATACATATTTGAATGTATTTAGAAAAATAAACAAATAGGGGTTCCGCGCACATTTCCCC<br>GAAAAGTGCCACCTGACGTCTAAGAAACCATTATTATCATGACATTAACCTATAAAAATAGGCGTATCACGA<br>GGCCCTTTCGTC

Supplementary Table 7. Staple sequences for the tetrahedron of 52-bp edge length and the same tetrahedron with overhangs.

(Orange text indicates ps tract for the DNA wrapping domain while green text denotes overhangs for the DNA hybridization domain)

Staple ID	Length (nt)	Staple sequences (5'-3')
1	34	GGAAACAGAGCTATTTTTTTTATTCTGACCGGGT
2	44	CGCTCATGACTTTTTTAGAAACGCTGGTGAAAGTATGGTCGCCC
3	32	TTATTCCGACAATAACAGAGAATTTTTCTAAA
4	56	GCGATGTGACTTTTTAAACGAATAGCCGCCCGGGTTTTTTTTGGCTCAACAACCCT
5	29	TTTGCGAAATCTGGCTCTCCACAGTACTC
6	55	ACCAGTCTTGCACAACATGCTTATGCGCTTGGTTGCCAAGAGTTTGAGGCCGCAT
7	42	TGTTCGCGGACGACATTCTCAGAATGACTCGGCCCTAAAGTT
8	48	ACTCGGTCGCCGCTTTATTGAATAGACTGGATGGCAAACGGTACTCTA
9	54	CCTATAGAAGGCGTTTTTTTTTGTTTTGAGTAGGAAGCCTTTTTTTGCATAAGT
10	42	TTGAGCTTAATAATAATGATGAGCACTTAGGGCGATGTTACT
11	56	TTCCCTATTTATCGGGACTCCAACGTCAATTAAAGTGGTTAATGTCATGAGGTTAC
12	49	TAGGTATCCTAGATTTTTTATATCTGCTATGTGGCGGAACGTGACCACG
12'	72	TTGTGACAGCTGGATCGTTACTTTAGGTATCCTAGATTTTTTATATCTGCTATGTGGCGGAACGTGACCACG
13	48	CGCCCCGAAGAACGGGCGATGCGGACATCTAGTGTTTGACTATAATTA
14	43	TGTATTTTTTTTCATTGTGCAAAGCAAATATTTTTTTCGTATC
15	45	ATCTGTGCGCAAACGCCCTCCCGTATCGAAAATCCTATTATCTTA
15'	68	TTGTGACAGCTGGATCGTTACTTATCTGTGCGCAAACGCCCTCCCGTATCGAAAATCCTATTATCTTA
16	42	TGGCGAAGAAAGTCCAAGAAAGCATGGTTCCTACTCCGAGAT
17	69	GGCCGAAATCGGCTAGTTATTGGCAACAACGTTGAAGAACCTAGTCACAGTCGTTAATGACCAAATCCT
18	57	TTACCCTCGATTTTTTCACAAGTAAATACGACAGTCTTTTTTTTGGCAATACAGGGA
19	42	AGCGGGCGATGAGATGTGTTGCAGGTAGTCTTCACGTAATTC
20	42	CCAAAATCCCGGTTGACGTAGCGAAGGAATAGTTAAGTGGCT
21	42	CCTCAGGTTAGTGACCAATCATATGTACCCCTTAACGAAAGG
22	40	TCGTTCCACTGACGAGAAAGGAAGGGGCAAGATGCAGTAA
23	46	GTAATTTTTTTTCAGTACTATATTAATACCGTTTTTTTTGTATCAT
24	48	CGCTGAATTGCGTTTTTTTTTGCGCTCATCCAAAGCTAGGGCTCATGA
25	36	GCCGCTTGCACTCTGAATCTGAATGAACTGATTAAG
25'	59	TTGTGACAGCTGGATCGTTACTTGCCGCTTGCACTCTGAATCTGAATGAACTGATTAAG
26	42	ATATATATGCCTCAGCCATACCAAACGAGAAACCTACAGGGC
27	41	GCACATTCGCGTAATTGATTTAAAACTTTAGACAGGACACC
28	48	CATTGGTAACTTTGGGAACCGGAGCTGTCTATATAATATTGATAATGC
29	46	GATAATTTTTTTATGCTTCAACAAACTAAGATTTTTTTGACAAAGC
30	44	AATTGTTCCTTGACGGGGAAAGCCGTTTAACGTGGGGTGCCGTA
31	59	ATTTGTTTATAAGATCAGTTGGGTGCCTCACCCGCGGCATTTTGCCTCCAATATGTATC
32	44	TACATTCAGAGGGTTCCTGTGCGCGGTTACATCGAACTCCCGAT
32'	67	TTGTGACAGCTGGATCGTTACTTTACATTCAGAGGGTTCCTGTGCGCGGTTACATCGAACTCCCGAT
33	44	TTTAAACAGGGGATCATGTAACTCGTTTTGATCGTCTGACAACG
34	42	GCAGGACCACAGCACCTGTATCGGCCTTAGGCGGATTCCGGC
35	42	TGGTCAGATCATGTTAGAAGTTAGAAATATAACCAAAAACCG
36	44	GTTGTACTACTTAAGCACTGGGGCCAGGAATAGATTAAAAGAAA
37	40	AAACCTTTTTTTAGGTACGAGCGAACCGAGCGTATCGCTG
38	23	AACGCAAAAGAAAGCGTGAGTTT
38'	57	A*A*A*A*A*A*A*A*A*A*A*A*A*A*A*A*A*A*A*A*A*A*A*A*A*A*A*A*A*A*TTTTAACGCAAAAGAAAGCGTGAGTTT
39	53	TGAGTATTCATTTTTTTCCGTCAGTGCTGCCATAACCATTTTTAACACTGCGG
40	45	AGGGTTGTCATTGCCTCTAGCTTCCCTTTAACAATTCTGATAAAT
41	53	GGTTGGTTTGTTTTACGTCTGGGCCCACTACGTGAACCATTTTAATCAAGTTT
42	39	AGAGGTTGGTCCTTATAAATCAAAAATGGTAATATTAAC
43	45	ACGATGTTTGTAGCAACTACACGACGGGGAGTCATTCTATGGATG
44	37	GCGTTAATGATAATCGCTGGCAAGTGTTTTGCGGTCA
45	31	TTAGAGCTGTTTTTGACGAGTGGGAACCCCT
46	47	TGGCTGGATACACTGACGAATTCGACGAAAGGGCCTTTTTTGATACG
47	47	TCTATCAGTTTTCCGGTTTCTTAGACGTCAGGTGGTTTTTTTTTCGG
48	42	AGATAGGCTTTAGACCACCACACCCGCCGCGCTAATTTACTC
49	32	AGAGAATTAAAAGATGCTGAAGTAAATTACAT
50	32	TTTGGGGTCGAGCGTCAGACCCGTAACTCCCG
51	32	CCAACTTACTGTCAGACCAAGAAGGAAGAGTA
52	57	AAGCACTAATTAACCCTAAAGGGAGCCGGATCTCAACAGCGGTTTCTTGAGAGTTTT
53	53	CTGGAGCCGTTCGTGGGTCTCGCGGTAAGTGTTGTTCCAGTTTTAGAGTCCAC
54	37	TATTAAACGGTATTATCCCGTATTTCGGGCAAGAGCA
55	56	ATCGGAGGATTGGAGCTAACCGCTTTTACAGAAAAGCATCTTATTGCATGACAGTA
56	48	AACGAAACATTTTTAATTTAAAATAGGTGAAGATCCTTTTATTTTTGT
57	22	TAAATCAGTTTTTTAACCAATA

Supplementary Table 8. Staple sequences for the pentagonal pyramid of 63-bp edge length and the same pentagonal pyramid with DNA wrapping domain.

(Orange text indicates ps tract for the DNA wrapping domain with orange whereas green text indicates overhangs for the DNA hybridization domain)

Staple ID	Length (nt)	Staple sequences (5’-3’)
1	57	GCACGAGTGTTACATCGAACTGGATCTATGAGCACTTTTTTAAGTTCTGCTAGCAAG
2	48	AACGTTTTCCAATGCAACAGCGGTAAGATCCTTGAGAGTTGAACAAGA
3	36	GTCCACTAGTGTTGTTCCAGTTTGTTCGCCCCGAAG
3’	51	GCCATGAAAGAGAATATCCGTGTCAGTCCACTAGTGTTGTTCCAGTTTGTTCGCCCCGAAG
4	31	TAGACCTTTTTGATAGGGTTGATTAAAGAAC
4’	65	A*A*A*A*A*A*A*A*A*A*A*A*A*A*A*A*A*A*A*A*A*A*A*A*A*A*A*A*A*A*TTTTTAGACCTTTTTGATAGGGTTGATT AAAGAAC
5	57	GTGGACTCCTGTCAAAGGGCGAAAAACTTCGCGTTAATTTTTTTGTTAAATCAAGAA
6	48	CCCTTATAAATCAAAGCTCATTTTTTAACCAATAGGCCGATACTTTAG
7	36	ATTGATTTCAAGTTTACTCATATAAATCGGCAAAAT
7’	36	AGCTGGATCGTTACATTGATTTCAAGTTTACTCATATAAATCGGCAAAAT
8	52	TGGTATTTTTACTGTCAGACAAAACTTCATTTTTTTTTTTAATTTAACGGCTG
AF647-8	52	/5Alex647N/TGGTATTTTTACTGTCAGACAAAACTTCATTTTTTTTTTTAATTTAACGGCTG
9	25	GACGGTTTTGGAGTCAGGCAAGCAT
9’	59	GACGGTTTTGGAGTCAGGCAAGCATTTTA*A*A*A*A*A*A*A*A*A*A*A*A*A*A*A*A*A*A*A*A*A*A*A*A*A*A*A*A*A *A
9-biotin	29	GACGGTTTTGGAGTCAGGCAAGCAT TTTT /3Bio/
10	48	GTGCCTCACTGATTAACTATGGATGAACGAAATAGACAGACCCGTATT
10’	62	AGCTGGATCGTTACGTGCCTCACTGATTAACTATGGATGAACGAAATAGACAGACCCGTATT
11	36	GACGCCGGTGTGGCGCGGTATTATTCGCTGAGATAG
12	42	CCCGGTTGTAACGTCTATCAGGGCGATGGCCCTCATATGTAC
13	42	TCAGACCCCAAACTACGTGAACCATCACCCTACCACTGAGCG
14	31	CTTAACTTTTTGAGTTTTCGTTATCAAGTTT
14’	65	A*A*A*A*A*A*A*A*A*A*A*A*A*A*A*A*A*A*A*A*A*A*A*A*A*A*A*A*A*A*TTTTCTTAACTTTTTGAGTTTTCGTTATC AAGTTT
15	57	TTTGGGGTCTGTGCCGTAAAGCACTAAGGCAACAATTTTTTAGACTGGATGGAATCC
16	42	CTCATGACCAAAGGCGGATAAAGTTGCAGGACTTTTGATAAT
17	42	GTGAAGATCCTCACTTCTGCGCTCGGCCCTTCAAGGATCTAG
17’	95	A*A*A*A*A*A*A*A*A*A*A*A*A*A*A*A*A*A*A*A*A*A*A*A*A*A*A*A*A*A*A*A*A*A*A*A*A*A*A*A*A*A*A*A*A* A*A*A*A*A*TTTGTGAAGATCCTCACTTCTGCGCTCGGCCCTTCAAGGATCTAG
17”	56	AGCTGGATCGTTACGAAGATCCTCACTTCTGCGCTCGGCCCTTCAAGGATCTAGGT
18	42	TCTAGCTTCCCATCGGAACCCTAAAGGGAGCCAACTACTTAC
19	42	ATTAACTGGCGCCCGATTTAGAGCTTGACGGGTGCGCAAACT
20	31	GTAGCATTTTTGGCAACAACGTGAAAGCCGG
20’	65	A*A*A*A*A*A*A*A*A*A*A*A*A*A*A*A*A*A*A*A*A*A*A*A*A*A*A*A*A*A*TTTTGTAGCATTTTTGGCAACAACGTGA AAGCCGG
21	57	CGAACGTGGTGAAAGGAAGGGAAGAAAGATCATGTAATTTTGCCTTGATCGTTGCCT
22	48	GCGTGACACCACGATGGGAACCGGAGCTGAATGAAGCCATTGAGCGTG
23	36	GGTCTCGCATAAATCTGGAGCCGGACCAAACGACGA
24	52	GCTGGTTTTTTTTATTGCTGGGTATCATTGCTTTTTAGCACTGGGGCTACAC
25	42	ACAACATGGGGGCGAAAGGAGCGGGCGCTAGGGCTTTTTTGC
26	42	AGGAGCTAACCGCGCTGGCAAGTGTAGCGGTCGGAGGACCGA
27	31	CTTACTTTTTTTGACAACGATCACGCTGCGC
27’	65	A*A*A*A*A*A*A*A*A*A*A*A*A*A*A*A*A*A*A*A*A*A*A*A*A*A*A*A*A*A*TTTTCTTACTTTTTTTGACAACGATCAC GCTGCGC
28	57	GTAACCACCTCCCGCCGCGCTTAATGCAGAAAAGCATTTTTACGGATGGCATGCCAA
29	48	GTGATAACACTGCGGACAGTAAGAGAATTATGCAGTGCTGCCCGTATC
30	36	GTAGTTATCCAGATGGTAAGCCCTCCATAACCATGA
31	42	TCACCAGTCACGCCGCTACAGGGCGCACATTAGGTTGAGTAC
32	42	ATGACTTATTGCGTTGCGCTCTGAAGATACACTATTCTCAGA
33	31	AGCAACTTTTTGGTCGCCGCATCAGTTGGGT
33’	65	A*A*A*A*A*A*A*A*A*A*A*A*A*A*A*A*A*A*A*A*A*A*A*A*A*A*A*A*A*A*TTTTAGCAACTTTTTGGTCGCCGCATCA GTTGGGT
33-biotin	35	/5Bio/ TTTTAGCAACTTTTTGGTCGCCGCATCAGTTGGGT

Supplementary Table 9. DNA sequences for magnetic capture and toehold mediated strand displacement.

(Red text indicates the DNA hybridization domain, violet text denotes the toehold sequence and green text is used for the staple sequences. X means the length of staple sequence)

Name	Length (nt)	Sequences (5'-3')
Overhang staple	25 + X	GCCATGAAAGAGAATATCCGTGTCA + Staple sequence
Bead strand	35	TGACACGGATATTCTCTTTCATGGCTCTCATTTTT/3Bio/
Bead invader	30	TGAGAGCCATGAAAGAGAATATCCGTGTCA

Supplementary Table 10. DNA sequences for QD-DNA complex constructs.

(Hybridization domains are indicated with green text. DNA wrapping domains are indicated as orange text. Valence domains are denoted with red text)

Name	Length (nt)	Sequences (5'-3')
po1-DNA1	55	TTGTGACAGCTGGATCGTTACCGGGACCGGCGA*A*A*A*A*A*A*A*A*A*A*GCGACGGGCGC
po1-DNA2	55	TTGTGACAGCTGGATCGTTACCGCGTGGAGCGA*A*A*A*A*A*A*A*A*A*A*CGCCG GTCCCG
po1-DNA3	55	TTGTGACAGCTGGATCGTTACCGTTGCCACGCA*A*A*A*A*A*A*A*A*A*A* CGCTCCACGCG
po1-DNA4	55	TTGTGACAGCTGGATCGTTACGCGCCCGTCGCA*A*A*A*A*A*A*A*A*A*A* GCGTGGCAACG
DNA2	34	CGCGTGGAGCGA*A*A*A*A*A*A*A*A*A*A*CGCCG GTCCCG
DNA3	34	CGTTGCCACGCA*A*A*A*A*A*A*A*A*A*A* CGCTCCACGCG
DNA4	34	GCGCCCGTCGCA*A*A*A*A*A*A*A*A*A*A* GCGTGGCAACG

Supplementary Table 11. Atomic mass and general LJ force field parameters.

(Labels are shown in Supplementary Fig. 52)

Atom label	Compound	Mass	LJ-ε (kcal/mol)	LJ-σ (Å)
Na	Na	22.99	0.160714286	1.897438886
c3	C	12.01115	0.0148	3.6170488
c4	C	12.01115	0.0148	3.6170488
n2	N	14.0067	0.016699997	3.501232007
n1	N	14.0067	0.016699997	3.501232007
h1	H	1.00797	0.0038	2.449971454
h2	H	1.00797	0	0
c1	C	12.01115	0.0039	3.875409464
c2	C	12.01115	0.0039	3.875409464
o1	O	15.9994	0.022800001	2.859784872
p	P	30.9738	0.019999768	3.741778233
o2	O	15.9994	0.022800001	2.859784872
s1	S	32.06	0.004297591	3.3677544
Zn	Zn	65.38	35.7426	0.02
S	S	32.06	0.0327669	4.9

Supplementary Table 12. LJ force field parameters for interfacial interactions.

(Labels are shown in Supplementary Fig. 52)

Interfacial Interaction			
Atom label	ZnS NP	LJ-ε (kcal/mol)	LJ-σ (Å)
Na	Zn	1.774823935	0.954969443
c3	Zn	0.53859075	1.8147744
c4	Zn	0.53859075	1.8147744
n2	Zn	0.572118825	1.756866003
n1	Zn	0.572118825	1.756866003
h1	Zn	0.272910246	1.231235727
h2	Zn	0	0.00625
c1	Zn	0.27647783	1.943954732
c2	Zn	0.27647783	1.943954732
o1	Zn	0.668490856	1.436142436
p	Zn	0.626095407	1.877139117
o2	Zn	0.668490856	1.436142436
s1	Zn	10	1.6901272

Supplementary Table 13. Harmonic bond force field.

(Labels are shown in Supplementary Fig. 52)

Harmonic Bond		
Bonding atom label	K_b (kcal/mol/Å^2)	r_0 (Å)
c3-c3	280	1.39
c3-c4	280	1.34
c3-n1	320	1.38
c3-h1	363.4164	1.08
c4-n2	388	1.32
c4-n1	560	1.26
c4-h1	363.4164	1.08
n2-h2	457.4592	1.026
n1-c1	336.8	1.475
c1-c1	322.7158	1.526
c1-o1	273.2	1.425
c1-c2	322.7158	1.526
h1-c1	340.6175	1.105
h1-c2	340.6175	1.105
c2-o1	273.2	1.425
o1-p	245.2	1.61
p-o2	480	1.53
p-s1	147.12	2.05275

Supplementary Table 14. Harmonic angle force field.

(Labels are shown in Supplementary Fig. 52)

Harmonic Angle		
Angle atom label	K_a (kcal/mol/radian^2)	θ_0 (degree)
c3-c3-c4	90	120
c3-c3-n1	90	120
c4-c3-n1	90	120
n1-c3-n1	102	134
n1-c3-h1	40	120
c3-c4-n2	102	120
c3-c4-n1	90	120
n2-c4-n1	102	120
n2-c4-n2	102	134
n1-c4-h1	50	120
c4-n2-h2	37.5	115
h2-n2-h2	33	125
c4-n1-c4	75.1	114
c3-n1-c4	75.1	114
c3-n1-c3	75.1	114
c3-n1-c1	70	128.8
c1-c1-o1	70	109.5
c1-c1-c2	46.6	110.5
h1-c1-c1	44.4	110
c2-c1-o1	70	109.5
h1-c1-o1	57	109.5
h1-c1-c2	44.4	110
c1-c2-c1	46.6	110.5
h1-c2-c1	44.4	110
h1-c2-h1	39.5	106.4
n1-c1-c2	80	109.5
n1-c1-o1	80	109.5
n1-c1-h1	80	109.5
c1-o1-c1	60	109.5
c1-c2-o1	70	109.5
h1-c2-o1	57	109.5
c2-o1-p	72	120
o2-p-o1	110	109.5
o1-p-o1	110	109.5
o2-p-o2	110	109.5
c1-o1-p	72	120
o1-p-s1	110	109.5

Supplementary Table 15. Harmonic dihedral force field.

(Labels are shown in Supplementary Fig. 52)

Harmonic Dihedral			
Dihedral atom label	K_d (kcal/mol)	*d*	*n*
c4-c3-c3-n1	3	-1	2
n1-c3-c3-n1	3	-1	2
c3-c3-c4-n2	3	-1	2
c3-c3-c4-n1	3	-1	2
n1-c3-c4-n2	3	-1	2
n1-c3-c4-n1	3	-1	2
c3-c3-n1-c3	2	-1	2
c4-c3-n1-c3	2	-1	2
c3-c3-n1-c4	2	-1	2
n1-c3-n1-c4	2	-1	2
c3-c3-n1-c1	2	-1	2
n1-c3-n1-c3	2	-1	2
n1-c3-n1-c1	2	-1	2
h1-c3-n1-c3	2	-1	2
h1-c3-n1-c1	2	-1	2
c1-c4-n2-h2	2.5	-1	2
n1-c4-n2-h2	2.5	-1	2
c3-c4-n1-c4	2	-1	2
n2-c4-n1-c4	2	-1	2
n1-c4-n1-c4	2	-1	2
h1-c4-n1-c4	2	-1	2
n1-c4-n1-c3	2	-1	2
h1-c4-n1-c3	2	-1	2
c3-n1-c1-c2	0.1	1	3
c3-n1-c1-o2	0.1	1	3
c3-n1-c1-h1	0.1	1	3
c2-c1-c1-o2	0.1581	1	3
h1-c1-c1-o2	0.1581	1	3
c2-c1-c1-c2	0.1581	1	3
h1-c1-c1-c2	0.1581	1	3
h1-c1-c1-h1	0.1581	1	3
c1-c1-o1-c1	0.13	1	3
c2-c1-o1-c1	0.13	1	3
h1-c1-o1-c1	0.13	1	3
c1-c1-c2-o1	0.1581	1	3
c1-c1-c2-h1	0.1581	1	3
o1-c1-c2-o1	0.1581	1	3
o1-c1-c2-h1	0.1581	1	3
h1-c1-c2-o1	0.1581	1	3
h1-c1-c2-h1	0.1581	1	3
c1-c1-c2-c1	0.1581	1	3
h1-c1-c2-c1	0.1581	1	3
n1-c1-c2-c1	0.1581	1	3
o1-c1-c2-c1	0.1581	1	3
n1-c1-c2-h1	0.1581	1	3
n1-c1-o1-c1	0.13	1	3
c1-c2-o1-p	0.13	1	3
h1-c2-o1-p	0.13	1	3
c2-o1-p-o2	0.25	1	3
c2-o1-p-o1	0.25	1	3
c1-o1-p-o1	0.25	1	3
c1-o1-p-o2	0.25	1	3
c2-c1-o1-p	0.13	1	3
c1-c1-o1-p	0.13	1	3
h1-c1-o1-p	0.13	1	3
o1-c1-c1-o1	0.1581	1	3
c2-o1-p-s1	0.25	1	3
c1-o1-p-s1	0.25	1	3

Supplementary Table 16. CVFF improper force field.

(Labels are shown in Supplementary Fig. 52)

CVFF Improper			
Improper atom label	K_i (kcal/mol)	*d*	*n*
c3-c3-c4-n1	0.37	-1	2
c3-c3-n1-n1	0.37	-1	2
h1-c3-n1-n1	0.37	-1	2
n1-c4-c3-n2	0.37	-1	2
h1-c4-n1-n1	0.37	-1	2
c4-n2-h2-h2	0.37	-1	2
c1-n1-c3-c3	0.37	-1	2

Supplementary References

1. Zhang, C. Y., Yeh, H. C., Kuroki, M. T. & Wang, T. H. Single-quantum-dot-based DNA nanosensor. *Nat. Mater.* **4**, 826–831 (2005).

2. Shen, J. *et al.* Valence-engineering of quantum dots using programmable DNA scaffolds. *Angew. Chem. Int. Ed.* **56**, 16077–16081 (2017).

3. BIOVIA, Dassault Systèmes, Materials Studio, Version 8, San Diego: Dassault Systèmes, (2014).

4. Plimpton, S., Crozier, P. & Thompson, A. *LAMMPS-large-scale Atomic/Molecular Massively Parallel Simulator*. (Sandia National Laboratories, 2007).

5. Li, Y., Liu, Z., Yu, G., Jiang, W. & Mao, C. Self-assembly of molecule-like nanoparticle clusters directed by DNA nanocages. *J. Am. Chem. Soc.* **137**, 4320–4323 (2015).

6. Tikhomirov, G. *et al.* DNA-based programming of quantum dot valency, self-assembly and luminescence. *Nat. Nanotechnol.* **6**, 485–490 (2011).

7. Farlow, J. *et al.* Formation of targeted monovalent quantum dots by steric exclusion. *Nat. Methods* **10**, 1203–1205 (2013).

8. Yu, W. W., Qu, L., Guo, W. & Peng, X. Experimental determination of the extinction coefficient of CdTe, CdSe, and CdS nanocrystals. *Chem. Mater.* **15**, 2854–2860 (2003).

9. Würth, C., Grabolle, M., Pauli, J., Spieles, M. & Resch-Genger, U. Relative and absolute determination of fluorescence quantum yields of transparent samples. *Nat. Protoc.* **8**, 1535–1550 (2013).
