# Supplementary material for: Nanoscale 3D spatial addressing and valence control of quantum dots using wireframe DNA origami
Source: Nat Commun. 2022 Aug 23;13:4935. doi: 10.1038/s41467-022-32662-w (PMC9399249; doi:10.1038/s41467-022-32662-w)
Supplement: Supplementary file 3 — Description of Additional Supplementary Files [file 41467_2022_32662_MOESM3_ESM.docx]

**Description of Additional Supplementary Files**

**File Name: Supplementary Movie 1
Description:** Structures of ssDNAwrapped 6 nm diameter QD. From left to right: chimeric ssDNA containing 5, 10, and 30 nt A* and fixed po domain (23 nt).

**File Name: Supplementary Movie 2
Description:** The process of chimeric ssDNA containing 30 nt A* and fixed po domain (23 nt) wrapped on 6 nm diameter QD.
